# Estimating cis and trans contributions to differences in gene regulation

**DOI:** 10.1093/genetics/iyag228

**Published:** 2026-09-11

**Authors:** Ingileif B Hallgrímsdóttir, Maria Carilli, Lior Pachter

**Affiliations:** Division of Biology and Biological Engineering, California Institute of Technology, Pasadena, CA 91125, United States; Division of Biology and Biological Engineering, California Institute of Technology, Pasadena, CA 91125, United States; Division of Biology and Biological Engineering, California Institute of Technology, Pasadena, CA 91125, United States; Department of Computing and Mathematical Sciences, California Institute of Technology, Pasadena, CA 91125, United States

**Keywords:** gene regulation, statistical method, RNA-seq

## Abstract

We describe a coordinate system and associated hypothesis-testing framework for determining whether *cis* or *trans* regulation is responsible for differences in gene expression between two homozygous strains or species. We apply our framework to data from single replicate studies on yeast strains and human-chimpanzee hybrid cells, as well as to data from a mouse study with replicates, showing marked differences between our gene regulatory assignments and those previously reported. We also show how our multisample framework can determine the context dependency of *cis* and *trans* effects as well as explicitly model different hypotheses regarding the underlying mechanism of *trans* regulation.

## Introduction

In 1961, Jacob and Monod developed a theory of gene regulation in which they distinguished local effects (*cis*) from distal regulation (*trans*) (Jacob and Monod 1961). Their work immediately raised the question of the relative contributions of these two regulation modalities (Phillips 1966; Kovach et al. 1973). One approach for assessing whether *cis* or *trans* regulation is responsible for differences in gene expression between strains or species is to compare differences in expression of genes in parents to allele-specific differences in $F_{1}$ hybrids. This approach was explored in Cowles et al. (2002), who used crosses of C57BL/6J and CAST/Ei mice to study regulatory mechanisms that could explain differences in gene expression between parental strains. Their approach was developed in Wittkopp et al. (2004) and Landry et al. (2005), who used pyrosequencing to study the regulation differences between *D. melanogaster* and *D. simulans*. With the advent of RNA-seq, genome-wide scans were possible, and Goncalves et al. (2012) examined RNA-seq from $F_{1}$ crosses of C57BL/6J and CAST/EiJ to tease apart *cis* and *trans* contributions to gene expression differences between the parental strains. Similarly, Massouras et al. (2012) performed such an RNA-seq analysis using *Drosophila* lines.

Formally, the idea of using crosses to study *cis* and *trans* contributions to differences in gene expression between strains or species is as follows: consider a gene with expression $X_{P1}$ in a homozygous strain 1, $X_{P2}$ in a homozygous strain 2, expression $X_{H1}$ for the haplotype from strain 1 in the $F_{1}$ cross of strains 1 and 2, and expression $X_{H2}$ for the haplotype from strain 2 in the $F_{1}$ cross of strains 1 and 2. Let $R_{P}=\log_{2}\;(X_{P1}/X_{P2})$ and $R_{H}=\log_{2}\;(X_{H1}/X_{H2})$. That is, $R_{P}$ corresponds to the log2-fold-change difference in expression between the two parental strains, and $R_{H}$ to the log2-fold-change difference between the expression of the hybrid haplotypes in the $F_{1}$ offspring. The connection between $R_{P}$, $R_{H}$, and regulation is as follows: consider that a gene can be regulated via *cis*, *trans*, or both (Fig. 1a). Gene expression measurements in the parents and hybrid (Fig. 1b) can be used to infer the nature of regulation underlying the difference in expression in the parental strains (Fig. 1c). Specifically, amending the classification of Landry et al. (2005), we have:

- *conserved:* No change in gene expression indicating there has been no change in regulation, i.e. $R_{P}=0$ and $R_{H}=0$, which implies $R_{P}-R_{H}=0$.
- *cis:* The relative difference in gene expression between the parents is the same as between the haplotypes in the hybrid indicating that the difference in parents is due to local *cis* effects arising from changes only in cis-regulatory elements, i.e. $R_{H}\neq 0$, $R_{P}\neq 0$, and $R_{P}=R_{H}$ which implies $R_{P}-R_{H}=0$.
- *trans:* Gene expression from the two haplotypes in the hybrid is the same, indicating that differences between the parents resulted from nonlocal *trans* regulation arising from a change only in *trans*-regulatory elements, i.e. $R_{H}=0$ and $R_{P}\neq 0$, which implies $R_{P}-R_{H}\neq 0$.
- *cis + trans:*  $R_{H}\neq 0$ and $R_{P}\neq R_{H}$ with $\text{sgn}(R_{H})=\text{sgn}(R_{P}-R_{H})$ arises as a result of change in both *cis*- and *trans*-regulatory elements with changes in *cis* and *trans* contributing to changes in gene expression between strains in the same direction.
- *cis* × *trans:*  $R_{H}\neq 0$ and $R_{P}\neq R_{H}$ with $\text{sgn}(R_{H})\neq \text{sgn}(R_{P}-R_{H})$ arises as a result of compensatory change in both *cis*- and *trans*-regulatory elements with changes in *cis* and *trans* contributing to changes in gene expression between strains in the opposite direction.

**Fig. 1.**
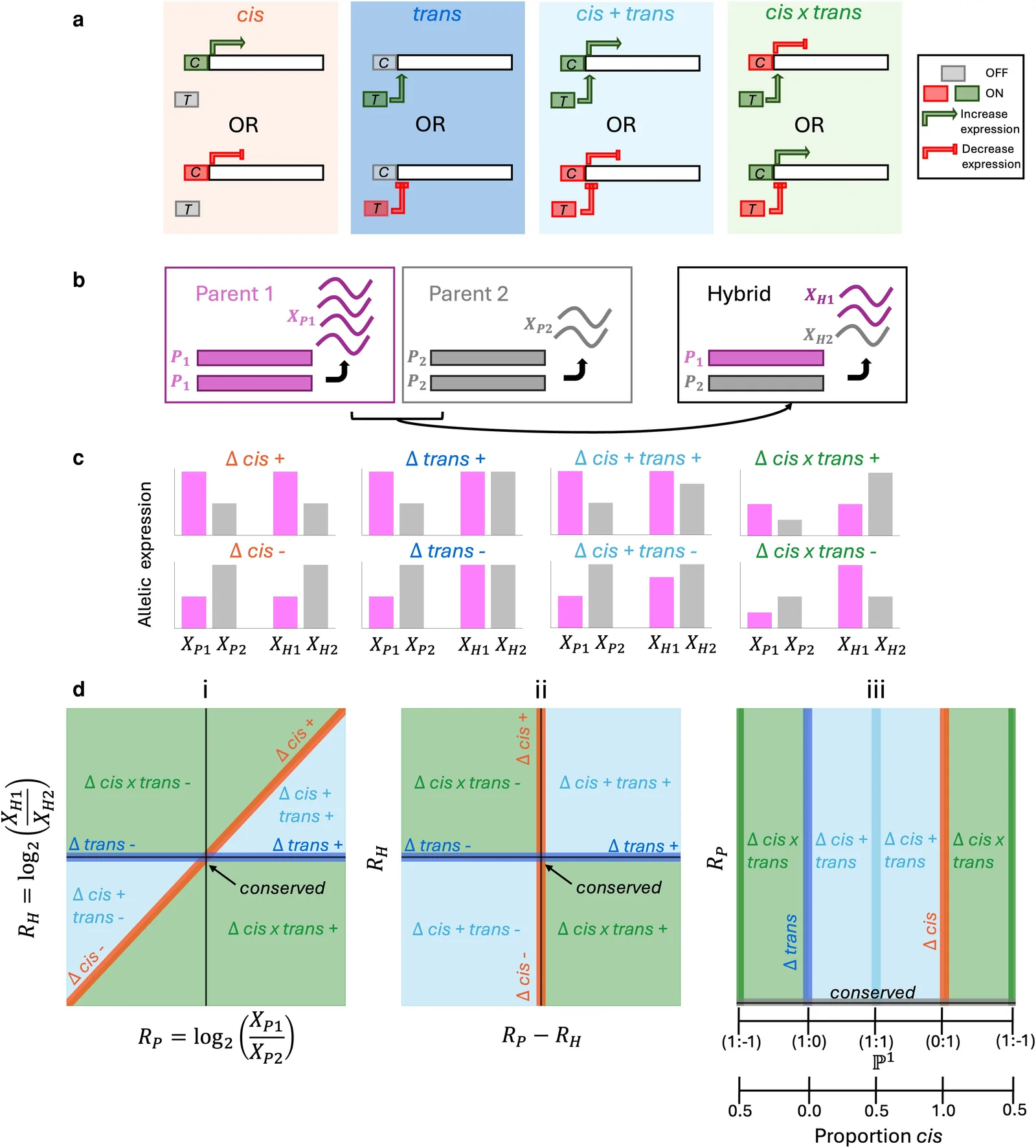
Geometry of parental and hybrid expression ratios can be used to assess regulatory differences between homozygous crosses. a) Diagram of the *cis*, *trans*, *cis + trans*, and cis × trans types of regulatory differences. b) Homozygous parents with haplotypes $P_{1}$ and $P_{2}$, counts of RNA molecules for a gene ($X_{P1}$ and $X_{P2}$, respectively), and the haplotypes in an $F_{1}$ hybrid along with counts for the gene ($X_{H1}$ and $X_{H2}$). c) Differences in regulation between the parents are reflected in distinct ratios between counts $X_{P1},X_{P2}$ and $X_{H1},X_{H2}$. D. i) Illustration of how regulation differences emerge in log-fold changes $R_{P}$ and $R_{H}$, D. ii) Linear transformation of $R_{P}=\log_{2}\;(X_{P1}/X_{P2})$ and $R_{H}=\log_{2}\;(X_{H1}/X_{H2})$ to yield orthogonal *cis* and *trans* coordinates, D. iii) Representation of proportion *cis* in real projective space $P^{1}$.

## Materials and methods

### Acquisition and preprocessing of data from yeast crosses

Processed RNA-seq data from Tsouris et al. (2024) were obtained from http://1002genomes.u-strasbg.fr/files/Diallel_RNAseq/ASE in the file “Datafile2_ase_sum_20230609.tab.” This file included reported values for allelic expression in parents and expression per parental allele in hybrids for 179 unique parent-hybrid trios and 285,777 unique gene-trio combinations. Details on the estimation of allelic expression are described in the original study (Tsouris et al. 2024). As integer value counts are required for the binomial and the two sample binomial ratio statistical tests, reported values were rounded to the nearest integer before statistical tests were performed.

In Tsouris et al. (2024), seven categories were considered (reverse, attenuating, reinforcing, compensatory, cis only, trans only, and *null*). In order to compare their assignments with our categories, we mapped *cis only*  →  *cis*, trans only →  *trans*, and *null*  → conserved. For mixed categories (*reverse, attenuating, reinforcing*, and *compensatory*), we assigned *cis + trans* when $\text{sgn}(R_{P}-R_{H})=\text{sgn}(R_{H})$ and *cis × trans* otherwise.

### Acquisition of data from human–chimpanzee crosses

The reported log2-fold changes between human and chimpanzee parental gene expression ($R_{P}$) and hybrid allelic log2-fold changes for human-chimpanzee hybrid cell lines ($R_{H}$) were obtained from Barr et al. (2023) for 72 cell types and 14,487 genes per cell type. The reported log2-fold changes were used without modification to recalculate proportion *cis* as


(1) $$\text{proportion}\;cis=\frac{2}{\pi }\text{atan2}\left(|R_{H}|,|R_{P}-R_{H}|\right).$$

The provided data also included reported proportion *cis* per gene per cell type, defined as


(2) $$\text{proportion}\;cis\;(\text{Barr et al}.\;2023)=\frac{\left|R_{H}\right|}{\left|R_{H}|+|R_{P}-R_{H}\right|},$$

which were directly compared to Equation (1).

The maximum difference between $x\;:\;=\left|R_{H}\right|/\left(|R_{H}|+|R_{P}-R_{H}|\right)$ and $\left(2/\pi )\text{atan2}(|R_{H}|,|R_{P}-R_{H}|\right)$ is 0.045 at x=0.239 and x=0.761. The maximum of the ratio is 1.57 and occurs at x≈0.00006 (Fig. 4b).

### Acquisition of data from mouse crosses

Ballinger et al. (2023) performed a study to assess regulatory strategies driving environmental adaptations in house mice from two different climates. To do so, they created two inbred lines derived from wild house mice from Saratoga Springs, New York, USA (cold climate strain, referred to as NY or SARA mice) and Manaus, Amazonas, Brazil (warm climate strain, referred to as BZ or MANA mice) and crossed them. Mice from both inbred lines and their hybrid crosses were housed in warm (21 ^∘^ C) and cold (5 ^∘^ C) conditions, then liver and brown adipose tissue (BAT) samples from male and female mice were collected. We refer to the original publication (Ballinger et al. 2023) for a description of how RNA sequencing, alignment, and allele-specific expression (ASE) quantification for parental and hybrid tissue samples was performed.

In line with the standard in the field, they used the parental counts and ASE in the hybrids and performed three statistical tests to assign genes’ regulatory categories: first, they tested for differential expression between lines using only parental counts; second, they tested for differential expression between alleles in the hybrids using only hybrid ASE; and third, they tested for ratio differences between alleles in the parents and alleles in the hybrids using parental counts and ASE (they used *DESeq2*; Love et al. 2014; to perform the tests, for further details see the Methods of Ballinger et al. 2023). Genes were assigned as *cis, trans, cis & trans*, or *conserved or ambiguous* based on the outcomes of the three tests, as described in Ballinger et al. (2023). For consistent comparison, we collapse the categories *cis + trans* and *cis* × *trans* to *cis & trans*.

We obtained metadata, parental counts, and regulatory assignments for genes in the four separate environment (warm/cold) and organ (liver/BAT) combinations from the GitHub repository https://github.com/malballinger/BallingerMack_PNAS_2023/. We obtained ASE for hybrids from the authors directly. We applied our regulatory assignment framework (see the subsection *Generalized linear models (GLMs) for multiple samples*) to male mice (for each of the four environment–organ combinations there were 6 SARA mice, 6 MANA mice, and 6 hybrids, for a total of 18 mice and 24 rows in the design matrix, as each hybrid contributes expression levels for two alleles) for each environment/organ condition separately (liver/warm, liver/cold, BAT/warm, BAT/cold); and for all male samples together (total of 72 mice and 96 rows of the design matrix). We used the log-additive *trans* hypothesis model for testing all samples and condition-specific effects and for (liver/warm, liver/cold, and BAT/warm) male samples. For all samples from mice in the warm environment, there were 5,897 fit genes, and 5,970 genes for samples in the cold environment. We fit the log-additive, dominant, and free models to the BAT/cold male samples, with comparisons of fit weights, significance values, and gene regulatory assignments shown in Supplementary Figs. 4–6. All fits were implemented using our new XgeneR package with DESeq2 version 1.42.1 (Love et al. 2014).

### Statistical tests

For a given gene, the cis–trans analysis can be motivated by a genetic model for the expected normalized log-expression levels in the two parental strains and the two alleles of the F1 hybrid as


(3) $$\log_{2}\;(X_{P1})=\mu +c_{1}+t_{1},$$

(4) $$\log_{2}\;(X_{P2})=\mu +c_{2}+t_{2},$$

(5) $$\log_{2}\;(X_{H1})=\mu +c_{1}+\alpha \cdot (t_{1}+t_{2}),$$

(6) $$\log_{2}\;(X_{H2})=\mu +c_{2}+\alpha \cdot (t_{1}+t_{2}).$$

Here, *μ* is a baseline log-expression level, $c_{i}$ is the cis-regulatory contribution associated with strain *i*, and $t_{i}$ is similarly the trans-regulatory effect associated with strain *i*. The parameter *α* is set to either 1 or 1/2. Setting α=1/2 corresponds to an additive average of the two parental trans effects and α=1 corresponds to a co-dominant model of the trans effects. This full model is not identifiable from a single cross because four gene expression values ($X_{P1},X_{P2},X_{H1},X_{H2}$) are modeled with five parameters ($\mu ,c_{1},c_{2},t_{1},t_{2}$). However, we can reparameterize this model by defining $\beta =\mu +(c_{1}+c_{2})/2+\alpha (t_{1}+t_{2})$, the average gene expression level of the parental strains, and $\beta_{C}=c_{2}-c_{1}$ and $\beta_{T}=t_{2}-t_{1}$, the differences in cis and trans-regulatory contribution associated with each parental strain, respectively. In the case when α=1/2, we get


(7) $$\log_{2}\;(X_{P1})=\beta -\beta_{C}/2-\beta_{T}/2,$$

(8) $$\log_{2}\;(X_{P2})=\beta +\beta_{C}/2+\beta_{T}/2,$$

(9) $$\log_{2}\;(X_{H1})=\beta -\beta_{C}/2,$$

(10) $$\log_{2}\;(X_{H2})=\beta +\beta_{C}/2.$$

Although strain-specific cis and trans-regulatory effects are not identifiable, the differences between strains denoted by $\beta_{C}$ and $\beta_{T}$ are identifiable and they correspond to the log-ratio contrasts used in the field:


(11) $$R_{P}=\log_{2}\;\left(\frac{X_{P1}}{X_{P2}}\right)=(c_{1}-c_{2})+(t_{1}-t_{2}),$$

(12) $$R_{H}=\log_{2}\;\left(\frac{X_{H1}}{X_{H2}}\right)=c_{1}-c_{2},$$

(13) $$R_{P}-R_{H}=t_{1}-t_{2}.$$

Thus, the hybrid ASE contrast $R_{H}$ measures cis-regulatory divergence, while the difference between the parental and hybrid log-ratios, $R_{P}-R_{H}$, measures trans-regulatory divergence. In the traditional three-test framework, evidence for cis divergence is obtained by testing whether the two hybrid alleles differ, evidence for trans divergence is obtained by testing whether $R_{P}=R_{H}$, and differential expression between the two parental strains, equivalently $R_{P}=0$, is used as evidence for overall regulatory divergence.

#### GLMs for multiple samples

We propose a GLM framework for testing the null hypotheses of *no cis* and *no trans* regulation, both overall and condition-specific, when multiple samples per condition are available. Our framework differs from previous applications of GLMs for *cis/trans* distinction (Ballinger et al. 2023) by virtue of using one GLM for all data (parental counts, hybrid ASE values, with all conditions represented), rather than fitting three separate models to different subsets of the data (e.g. only parental counts for one test or only hybrid ASE values for another).

We do this by developing a model with weights for overall and condition-specific *cis* and *trans* regulatory effects, as well as including condition intercepts to properly attribute observed allele expression patterns to regulation or condition. In addition, our approach allows us to propose different hypotheses about the nature of the *trans* regulatory effect: if it is log-additive, dominant, or free (described below). To properly account for the overdispersion and discreteness of RNA-seq counts, we fit a GLM with a negative-binomial likelihood function and a log link function. For raw counts, the GLM includes a size-factor offset, so that $\log\;E(Y_{gj})=\log\;s_{j}+\eta_{gj}$, where $\eta_{gj}$ is specified by the design matrices below. Hybrid allele-specific count columns are treated as haplotype-specific pseudo-samples with their own DESeq2 size factors, so the two alleles of a hybrid sample are normalized by their respective haplotype-specific count totals rather than by duplicating the full hybrid sample size factor.

*One condition:* In the simplest case, we set the geometric mean of parental expression to be the intercept *β* and include weights for the *cis*-regulatory difference ($\beta_{C}$) and *trans* regulatory difference ($\beta_{T}$) between parental strains. This allows the normalized expression from the parental alleles ($X_{P1},X_{P2}$) and normalized allelic expression from the hybrids ($X_{H1},X_{H2}$) to be modeled under three different hypotheses of *trans* regulation.

The first hypothesis (*log-additive*) assumes that the *trans* expression difference between parental strains at each allele is influenced by both sets of parental chromosomes multiplicatively, and is thus reduced to the square root of the full parental effect in the hybrids. The design matrix is accordingly


(14) $$\log\;E\left[\begin{array}{c} X_{P1}\\ X_{P2}\\ X_{H1}\\ X_{H2} \end{array}\right]=\left[\begin{array}{ccc} 1 & -0.5 & -0.5\\ 1 & 0.5 & 0.5\\ 1 & -0.5 & 0\\ 1 & 0.5 & 0 \end{array}\right]\left[\begin{array}{c} \beta \\ \beta_{C}\\ \beta_{T} \end{array}\right].$$

A second hypothesis (*dominant*) assumes that the *trans* expression difference between parental strains is the same regardless of if both sets or one set of parental chromosomes are present: it is the same in the parents and hybrids, and the design matrix is


(15) $$\log\;E\left[\begin{array}{c} X_{P1}\\ X_{P2}\\ X_{H1}\\ X_{H2} \end{array}\right]=\left[\begin{array}{ccc} 1 & -0.5 & -0.5\\ 1 & 0.5 & 0.5\\ 1 & -0.5 & 0.5\\ 1 & 0.5 & 0.5 \end{array}\right]\left[\begin{array}{c} \beta \\ \beta_{C}\\ \beta_{T} \end{array}\right].$$

Finally, and most analogous to previous hypothesis-testing methods (Ballinger et al. 2023), *trans* expression differences between parental strains can have an unconstrained relationship (*free*) with their effects in the hybrids by allowing each hybrid sample to have a sample-specific effect shared by both hybrid alleles (denoted $\gamma_{i}$ below, with *i* indexing hybrid samples). The resulting design matrix is


(16) $$\log\;E\left[\begin{array}{c} X_{P1}\\ X_{P2}\\ X_{H1,i}\\ X_{H2,i} \end{array}\right]=\left[\begin{array}{cccc} 1 & -0.5 & -0.5 & 0\\ 1 & 0.5 & 0.5 & 0\\ 1 & -0.5 & 0 & 1\\ 1 & 0.5 & 0 & 1 \end{array}\right]\left[\begin{array}{c} \beta \\ \beta_{C}\\ \beta_{T}\\ \gamma_{i} \end{array}\right].$$

For all models, we can test the null hypothesis of *no cis* differences between parental strains by testing if $\beta_{C}=0$ and the null hypothesis of *no trans* differences by testing if $\beta_{T}=0$. We also report $\hat{\beta }$, $\hat{\beta_{C}}$, and $\hat{\beta_{T}}$ with standard error which allows us to consider the estimated fold change associated with cis and trans-regulatory divergence between the two parental strains. Note that testing $\beta_{C}=0$ is equivalent to testing $R_{H}=0$, and testing $\beta_{T}=0$ is equivalent to testing $R_{P}=R_{H}$. Testing $\beta_{C}=\beta_{T}=0$ is equivalent to simultaneously testing for no *cis* and no *trans* divergence; it implies $R_{P}=0$ but is stronger than testing parental equality alone. A test of parental differential expression corresponds to testing $\beta_{C}+\beta_{T}=0$, since $R_{P}=-(\beta_{C}+\beta_{T})$.

*Multiple conditions:* As more conditions are added to the experimental design, they can be naturally incorporated into the GLM framework, with condition-specific *cis* and *trans* weights as well as intercepts and interaction terms. For example, we fit data for liver and BAT samples for mice grown in warm and cold environments (four separate organ/environment) using the following model under the log-additive *trans* hypothesis:


(17) $$\begin{array}{rl} \log\;E[X] & ∼\beta +\beta_{C}+\beta_{T}+\beta_{CO}+\beta_{TO}+\\ & \;+\beta_{CE}+\beta_{TE}+\beta_{COE}+\beta_{TOE}+\beta_{O}+\beta_{E}+\beta_{OE}. \end{array}$$

The subscripts C,T,O, and *E* refer, respectively, to differential *cis* regulation between strains, differential *trans* regulation between strains, organ, and environment effects, with combinations denoting their interactions (e.g. $\beta_{CO}$ models the organ-specific *cis* effect). This is also compatible with any of the three previously described *trans* hypotheses (log-additive, dominant, and free).

*Implementations:* We implemented GLMs for the one condition in edgeR version 4.0.16 (Robinson et al. 2010) and DESeq2 version 1.42.1 (Love et al. 2014), showing differences in estimated gene variance, data likelihoods, and hypothesis test significance values in Supplementary Fig. 12. In particular, we tested significance using a likelihood ratio test (LRT) between the full and reduced models. Differences in significance values lead to differences in regulatory assignments, especially pertinent in our case in which both *no cis* and *no trans* hypothesis testing results are important for regulatory categorization. For the results reported here, we used DESeq2 for full model fits and regulatory assignments. We corrected LRT *p*-values for each hypothesis test for the number of genes tested using the Benjamini–Hochberg correction (Benjamini and Hochberg 1995) to obtain false discovery rates (FDRs), and rejected null hypotheses with FDR less than 0.05. These methods were implemented in a new R package XgeneR, and a Python package, XgenePy (see Data and code availability).

#### Classifying genes based on statistical test results

The results of the two tests for single sample experiments or experiments with replicates are used to categorize genes as outlined in Table 1.

**Table 1.** Statistical assignments of gene regulatory differences.

Single sample: binomial test	Single sample: binomial ratio test	
Replicates: $\beta_{C}=0$	Replicates: $\beta_{T}=0$	
**(null: *no cis*)**	**(null: *no trans*)**	**Classification**
Fail to reject	Fail to reject	Conserved
Reject	Fail to reject	*cis*
Fail to reject	Reject	*trans*
Reject	Reject	*cis & trans* (*cis + trans* or *cis* × *trans* by geometry)

Genes assigned to *cis, trans, cis + trans* or *cis × trans* regulation based on two statistical tests, described above for single sample or replicate experiments. If both statistical tests are rejected, regulatory assignment can be made by locating the point $\left(R_{P}-R_{H},R_{H}\right)$ (see Results section, Geometry) in the transformed space and following the geometric assignments listed in Supplementary Table 1.

Note that some care must be taken when interpreting a *cis* or *trans* assignment within this hypothesis-testing framework. For example, a *cis* assignment is made when “no *cis*” can be rejected but “no *trans*” cannot be rejected. Failure to reject “no *trans*” does not imply that one can accept the null hypothesis, but we utilize the assignment of *cis* in this case for simplicity for users. However, this caveat should be taken into account when interpreting the assignments of *cis*, *trans*, and *cis* and *trans* assignments.

This classification corrects (Landry et al. 2005), which fails to properly assign regulation differences to *cis + trans* when both $R_{H}<0$ and $R_{P}<0$. The relationships between $R_{P}$ and $R_{H}$, in general, can be visualized as lines and regions in a 2D plot (Wittkopp et al. 2004), as illustrated in Fig. 1d(i). While this direct representation of $R_{P}$ and $R_{H}$ is useful, a quantitative assessment of the gene regulatory modalities reflected in $R_{P}$ and $R_{H}$ requires a biologically meaningful notion of distance between points in Fig. 1d(i). Consider, for example, the situation where $X_{P1}=2X_{P2}$ and $X_{H1}=2X_{H2}$, i.e. a 2-fold change in gene expression between the parents due solely to *cis* regulation which corresponds to the point (1,1) in Fig. 1d(i). The distance from this point to the origin (conserved) is $\sqrt{2}$, whereas the same 2-fold difference in gene expression in the parents due solely to *trans* regulation with $R_{P}=1$ and $R_{H}=0$ is distance 1 from the origin. This imbalance can be corrected via a linear transformation.

#### Binomial test

To test the null hypothesis that there is no difference between expression of parental alleles in hybrids (or that the difference in regulation is purely *trans*), a binomial test was performed on rounded integer counts from Tsouris et al. (2024) using the function scipy.stats.binomtest (Virtanen et al. 2020). This tests for the probability of having observed a value at least as extreme as *k* successes given probability *p* of success and a total of *N* trials by summing over binomial probabilities:


(18) $$P(k;N,p)=\left(\begin{array}{c} N\\ k \end{array}\right)p^{k}(1-p{)}^{N-k}.$$

If $X_{H1}$ is the allelic expression in the hybrid of one parental allele and $X_{H2}$ is the allelic expression in the hybrid of the other parental allele, the binomial test was performed with $k=X_{H1},N=X_{H1}+X_{H2}$, p=0.5, and a two-sided alternative hypothesis.

Differential expression levels between the two hybrid alleles can also be compared using a GLM.

#### Two-sample binomial ratio test

To test the null hypothesis that there is no difference in the ratio of expression of alleles in parents and allelic expression in hybrids (or that the difference in regulation between parents is purely cis), we performed a two-sample binomial ratio test in which we assumed both $X_{P1}$ and $X_{H1}$ are sampled from a binomial distribution with the same probability of success $p_{s}$. Using


(19) $$p_{s}=\frac{X_{P1}+X_{H1}}{X_{P1}+X_{P2}+X_{H1}+X_{H2}},$$

we calculated the grid of probabilities over possible $P_{1}$ and $H_{1}$ values:


(20) $$\begin{array}{rl} P(X_{P1} & =x_{P1},X_{H1}=x_{H1};N_{P},N_{H},p_{s})\\ & =\left(\begin{array}{c} N_{P}\\ x_{P1} \end{array}\right)p_{s}^{x_{P1}}(1-p_{s}{)}^{N_{P}-x_{P1}}\\ & \;\times \left(\begin{array}{c} N_{H}\\ x_{H1} \end{array}\right)p_{s}^{x_{H1}}(1-p_{s}{)}^{N_{H}-x_{H1}}, \end{array}$$

where $N_{P}=X_{P1}+X_{P2}$ is the total number of counts from parents and $N_{H}=X_{H1}+X_{H2}$ is the total number of counts from the hybrid. We then calculated the probability of having observed values at least as extreme as the observed $X_{P1}$ and $X_{H1}$.

To account for multiple testing, we corrected both binomial and two sample binomial significance values using the Benjamini–Hochberg correction for FDRs (Benjamini and Hochberg 1995). We rejected the null hypothesis for tests with FDRs less than 0.05.

## Simulation study

We performed genome-wide simulations of parental and F1 hybrid allele-specific RNA-seq count data. The parameters used in the simulation mimic a mouse cross but the results are applicable to other model organisms. We generated a total of 6,000 genes per simulated dataset. Genes were randomly assigned to low, medium, or high-expression classes with mean counts of 20, 100, or 500. We simulated 45 different parameter scenarios, varying biological replication per group (4, 6, 8, 16, and 32), negative-binomial dispersion (alpha = 0.02, 0.20, 0.50), ASE-informative read fraction (0.05, 0.20, 0.40), and absolute log2-fold cis and trans effect sizes (0.125, 0.25, 0.5, 1, 1.5, 2, and 3). For each of the 45 scenarios, we performed a full analysis on each of the 10 datasets using XgeneR and GLM-based tests for cis and trans effects and also an analysis using parent, ASE, and parent-vs-hybrid ratio tests. Performance was evaluated using power, type I error, receiver operating characteristic (ROC)/area under the curve (AUC), parameter recovery, and regulatory-class assignment accuracy. Full details of the data-generating model, parameter grid, and diagnostic results are provided in the Supplementary Simulation Figures and Simulation Methods.

## Results

### Geometry

Because cis–trans assignments are often interpreted visually in the $\left(R_{P},R_{H}\right)$ plane, it is important that the visualization place the cis and trans axes on comparable footing. To decouple the effects of *cis* and *trans* regulation on $R_{P}$ and $R_{H}$, we begin by noting that if the difference in parental expression is solely due to *cis* regulation, then $R_{P}=R_{H}$, or equivalently $R_{P}-R_{H}=0$ (orange vertical line in Fig. 1d(ii)). If the difference in parental expression is solely due to *trans* regulation, then $R_{H}=0$ (blue horizontal line in Fig. 1d(ii)). Therefore, the transformation from Fig. 1d(ii) to Fig. 1d(i) is obtained by


(21) $$\left(\begin{array}{c} R_{P}\\ R_{H} \end{array}\right)=\left(\begin{array}{cc} 1 & 1\\ 0 & 1 \end{array}\right)\left(\begin{array}{c} R_{P}-R_{H}\\ R_{H} \end{array}\right).$$

The inverse transformation from the coordinate system in Fig. 1d(i) to the coordinate system in Fig. 1d(ii) is given by


(22) $${\left[\begin{array}{cc} 1 & 1\\ 0 & 1 \end{array}\right]}^{-1}\;=\;\left[\begin{array}{cc} 1 & -1\\ 0 & 1 \end{array}\right],$$

i.e. the transformation inverts the mapping of the vertical axis to the diagonal *cis* line.

The determination of whether a difference in parental gene expression is due to *cis* or *trans* can now be understood to be an assessment of whether the line passing through the origin and a point $\left(R_{P}-R_{H},R_{H}\right)$ is a perturbation (due to noise in gene expression measurement) of the line *Δ trans*, the line *Δ* cis or sufficiently far away from the axes in Fig. 1d(ii) to merit a designation of *Δ cis + trans* or *Δ* cis × trans (the designations are enumerated in Supplementary Table 1). In other words, the sufficient statistic is a point in real projective space $P^{1}$ (Fig. 1d(iii)), and the proportion of the difference in gene expression between parents that can be attributed to *cis* can be understood to be a scaling of the angle of the line through the origin corresponding to the point in $P^{1}$, i.e.


(23) $$\text{proportion}\;cis=\frac{2}{\pi }\text{atan2}\left(|R_{H}|,|R_{P}-R_{H}|\right).$$

### Statistics

The determination of whether a measurement $\left(R_{P},R_{H}\right)$ reflects a difference in gene expression between parents due to *cis* or *trans* regulation, or both, requires a statistical assessment (McManus et al. 2010). Specifically, hypothesis tests can be used to reject a null hypothesis of a difference in gene expression being due solely to *trans* or solely to *cis*. Geometrically, as evident from the linear transformation on log-fold changes, these two tests correspond to testing whether one can reject the null hypotheses that $\left(R_{P}-R_{H},R_{H}\right)$ is located on the *x*- and *y*- axes, respectively. As explained in the Materials and methods, the failure to reject the null hypotheses “no *cis*” or “no *trans*” do not imply the acceptance of those nulls; our classifications reflect the statistical power of the experiment to reject the absence of *cis* or *trans* regulation, which decreases with smaller sample size and weaker effect sizes. We explore this with ground-truth simulations across a range of sample and effect sizes (see Supplementary Simulation Figures), but discuss discrete classifications for consistency with previous publications.

We first developed a framework for hypothesis testing in this case where there are no replicates of the gene expression measurements, as in Tsouris et al. (2024), which consists of bulk RNA-seq performed on hybrid and parental strains of genetically divergent *Saccharomyces cerevisiae* (see Methods, Supplementary Figs. 1 and 2). Briefly, Tsouris et al. (2024) generated hybrid crosses from 26 parental yeast isolates derived from diverse environmental conditions. In Tsouris et al. (2024), genes were classified according to the representation shown in Fig. 1d(i). First, both $R_{P}$ and $R_{H}$ were tested for statistically significant differences from 0 and assigned “null” (our *conserved*) if both tests failed to reject the null hypothesis (one-sample ASE tests); then, changes between parental and hybrid allelic ratios were tested for significance (two-sample ASE tests). For genes that passed significance thresholds, regulatory assignments were made by dividing the untransformed 2D plane into cones (Fig. 2a). We reassigned genes based on our hypothesis tests as derived from the transformed coordinate system (Fig. 2c), with one test with the null hypothesis of *cis* regulation and one with the null hypothesis of *trans* regulation, thereby putting the two regulation strategies on equal footing. Compared to the original study, we found major differences in assignment (Fig. 2b and Supplementary Fig. 3).

**Fig. 2.**
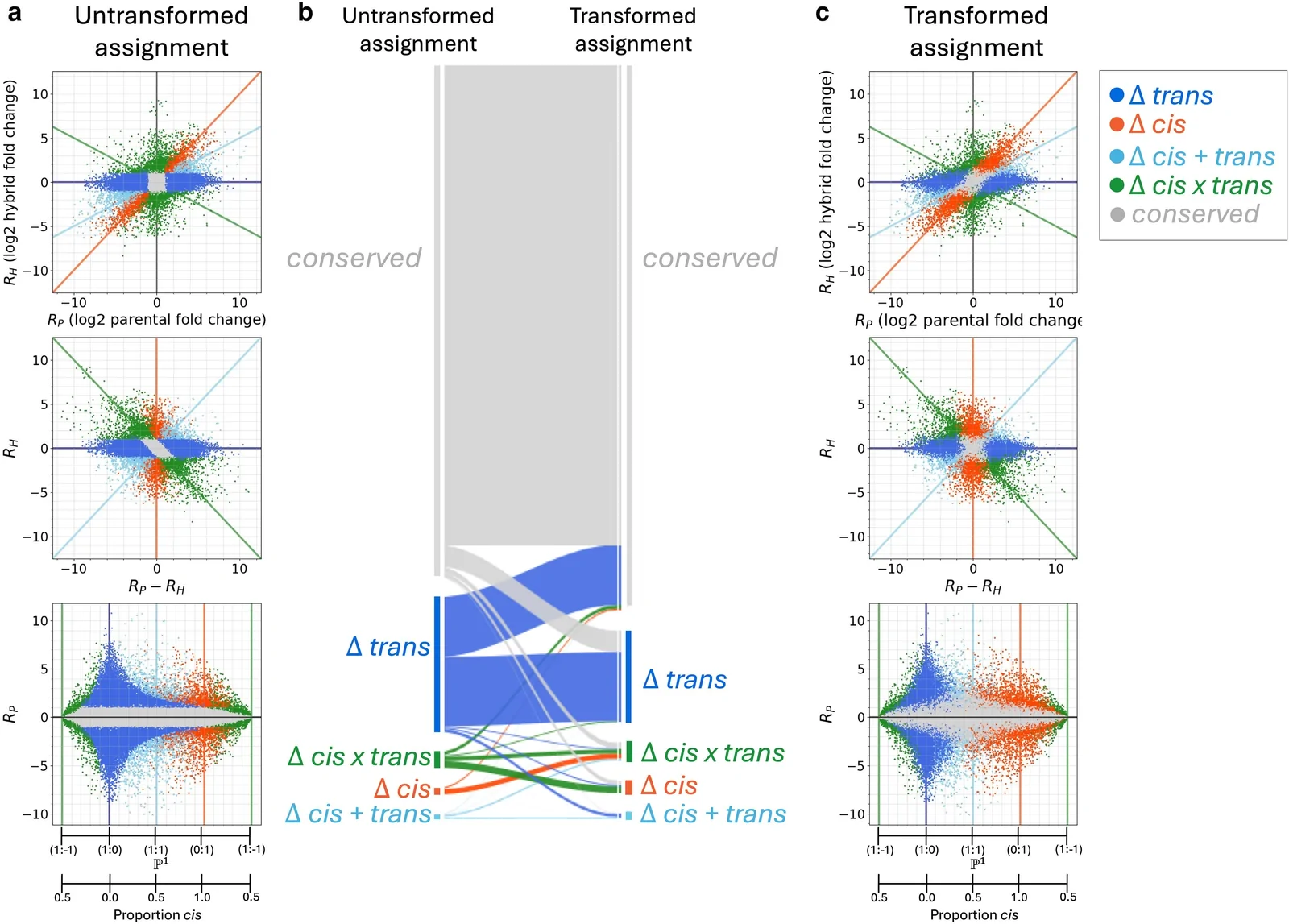
Contributions of *cis* and *trans* regulation to differences in gene expression between yeast strains, with data from Tsouris et al. (2024) (*n* = 285,777 gene-cross combinations from 179 unique yeast parent crosses). a) Untransformed ratios, transformed ratios, and log2 of parental fold change versus proportion *cis*, colored by regulatory assignments from Tsouris et al. (2024). b) Comparison of the results of Tsouris et al. (2024) to the assignment of genes based on the proposed hypothesis testing and geometric assignment procedure. c) Reanalysis of the 285,777 gene-cross expression data from Tsouris et al. (2024) using the proposed hypothesis testing and geometric assignment procedure.

Interestingly, whereas Tsouris et al. (2024) conclude that “the transcriptome is globally buffered at the genetic level mainly due to trans-regulatory variation in the population,” we find that a considerable amount of difference in gene expression can be attributed to *cis* (Supplementary Fig. 3c), with the difference due to Tsouris et al. (2024) deriving assignments in the untransformed coordinate system using a statistical testing procedure that treats *cis* and *trans* regulation asymmetrically. Specifically, Tsouris et al. (2024) report 57,253 cases where gene expression difference is due to *trans* regulation (Supplementary Fig. 3a). We find 39,063 cases that can be assigned to trans (Supplementary Fig. 3b). These numbers are similar; however, Tsouris et al. (2024) report 2,804 cases assigned to *cis* (Supplementary Fig. 3a), whereas we find *7,441* (Supplementary Fig. 3b). Furthermore, we find 2,313 cases assigned to *cis + trans* (Supplementary Fig. 3b) versus 1,727 in Tsouris et al. (2024) (Supplementary Fig. 3a). While these discrete classifications based on the outcomes of statistical tests can be compared, there is a range of proportions *cis* for genes within a classification (Fig. 2a and 2c). Notably, the *trans* classifications of Tsouris et al. (2024) extend up to a calculated proportion *cis* of almost 1.0 (Fig. 2a), while our assignments are restricted to a region closer to the *trans* axis, explaining the possible over-assignment of *trans* regulation in the original study. While it has been suggested that *trans* regulation accounts for more variation in expression within species and *cis* regulation accounts for interspecific divergence (Signor and Nuzhdin 2018), our results indicate that *cis* regulation, with still fewer assigned instances than *trans*, may play a larger role than previously assessed. Overall, there is a marked difference between our results and those of Tsouris et al. (2024) (Supplementary Fig. 3c).

In the case where replicates have been obtained, a GLM, as is commonly used for differential expression in bulk RNA-seq (Robinson et al. 2010; Love et al. 2014), can be adapted to utilize gene expression variance estimates within parents and hybrids. We tested our approach (see Methods) on data from Ballinger et al. (2023), in which two wild-derived mouse strains and their F1 hybrids were used to study the effect of genotype and environment (temperature) on gene expression divergence in rapidly evolving strains. Two inbred lines of house mice were derived from mice from a cold environment (Saratoga Springs, New York, USA) and a warm environment (Manaus, Amazonas, Brazil) (Ballinger et al. 2023), and two tissues, BAT and liver, were evaluated in each of the strains and their F1 hybrids, also raised at the two different temperatures. The experiment was performed in six replicates each of male and female parents, as well as six replicates each of male and female F1 hybrids (Ballinger et al. 2023) (Fig. 3a). The GLM framework allows the joint analysis of all samples (parents and hybrids) rather than requiring that separate tests be run on separate subsets of the data (e.g. differential expression between parents, differential expression between hybrid allelic expression, and a ratio test as in Ballinger et al. 2023). It further enables explicit modeling of specific biological hypotheses of *trans* regulatory action (see Methods and Supplementary Figs. 4–6).

**Fig. 3.**
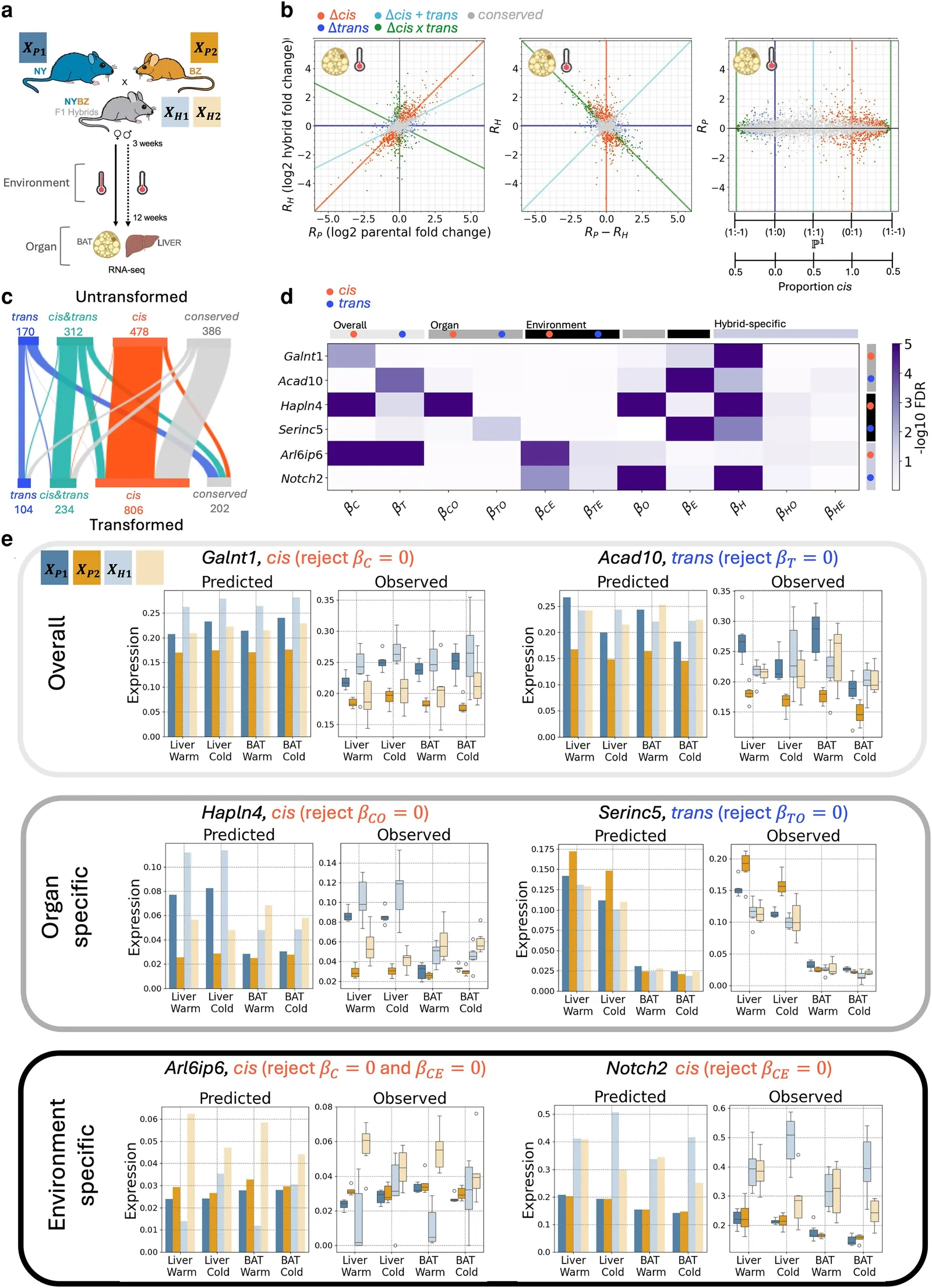
Multisample regulatory assignment can be determined using GLMs and condition-specific hypothesis tests. a) Cartoon of experimental design set up from original study (adapted from Fig. 1c of Ballinger et al. 2023). b) Model predicted ratios colored by GLM determined regulatory assignments as described in Methods. The free model was fit to BAT from male mice reared in the cold (n=6 NY mice, n=6 BZ mice, n=6 hybrid crosses, and n=5,970 genes). c) Untransformed (original) regulatory assignments (reported in Ballinger et al. 2023) derived from three independent model fits and statistical tests differ from transformed regulatory assignments derived using the unified GLM framework (genes and assignments from B, excepting genes that were categorized as conserved by both the original and our testing procedure, which are not shown). d) The free model was fit to samples from all four environment/organ pairs (BAT/cold, BAT/warm, liver/cold, liver/warm, n=6 NY/BZ/hybrid samples per condition) with weights for environment and organ-specific regulatory effects. The negative log10 of FDRs highlights several genes that exhibit significant *cis* or *trans* behavior overall or only in an organ or environment-specific manner. e) For the same genes, GLM predicted (bar plots) and observed gene expression (box plots with counts normalized by the total count per sample for parents and per haplotype for hybrids) for parental and hybrid alleles (n=6 samples per box, with the box showing values’ interquartile ranges and a line at the mean and whiskers extending to min/max values).

Restricting our analysis to a single tissue, temperature, and sex (BAT collected from male mice reared in the cold temperature), we fit a GLM with the free hypothesis and classified genes based on the reduced models of no *cis* or no *trans* regulation (see Methods, Fig. 3b). Consistent with our single sample analyses, we noted an increase in the number of *cis* assigned genes from 478 in the original study (Ballinger et al. 2023) to 806 (Fig. 3c). This trend held in different tissue and temperatures (Supplementary Fig. 7). We further fit the free model to all male samples, including environment (cold/warm) and organ (liver/BAT) specific *cis* and *trans* weights (Fig. 3d). We identified genes that exhibited significant overall *cis* and *trans* regulatory changes (Fig. 3e, top panel), as well as those that were only significantly different in one organ (Fig. 3e, middle panel). For environment-specific effects, no *trans* genes reached the significance level (FDR of 0.05), so we displayed genes with interesting patterns of significant environment-specific *cis* effects: one with both overall and environment-specific *cis* regulatory differences, and another with only significant regulatory differences in the patterns in a specific environment (Fig. 3e, bottom panel). Among these genes were several with functions related to body fat and temperature. For example, *Acad10*, which was identified as having an overall significant *trans* regulatory difference between the strains (Fig. 3e, top panel), codes for Acyl-coA dehydrogenase family member 10. This protein plays a role in lipid metabolism and fatty acid oxidation, and deficiency has been shown to lead to abnormal weight gain and deficiency in glucose tolerance in mice (Bloom et al. 2020). *Serinc5*, which was shown to have organ-specific regulatory differences between parental strains, codes for serine incorporator 5, a protein that incorporates serine into cell membranes to facilitate the synthesis of sphingolipids (Inuzuka et al. 2005), which themselves have been implicated in thermal adaptation and response to heat across eukaryotic organisms (Jenkins 2003; Fabri et al. 2020). Notably, *Notch2* was found to have only environment (temperature) specific and not overall *cis* regulatory differences between parental strains, concordant with Nomura et al. (2022) reporting temperature sensitivity of the Notch signaling pathway underlying species’ level differences in development and plasticity in mice. These genes are plausible candidates for differential regulation between mouse strains adapting to life in cold and warm environments. The flexibility of the GLM framework facilitates identification of genes with unique regulatory patterns, such as the tissue and organ-specific effects, a key improvement over post-hoc comparisons of multiple model fits that would be otherwise required.

### Proportion *cis*

In addition to naturally revealing the appropriate hypothesis tests to conduct for attribution of gene expression difference in parents to *cis* or *trans*, the linear transformation we propose leads directly to a meaningful measure of the proportion of difference in gene expression that can be attributed to *cis* regulation (or *trans*) (Equation (23)). To illustrate this, we re-analyzed a dataset of gene expression from human–chimpanzee cell line hybrids (Barr et al. 2023), calculating the proportion *cis* according to Equation (23). In Barr et al. (2023), the proportion *cis* was calculated using slope, as is natural to do when working in the untransformed coordinate framework, whereas the correct calculation (Equation (1)) uses angle in the transformed coordinate system. While the absolute differences are small (Fig. 4a), with a maximum difference of 0.045 (see Methods), the relative difference is large when the proportion *cis* is small (Fig. 4b), and can be as high as 57% (see Methods). While uncertainty in fold-change estimates due to low sample or effect sizes, experimental or technical noise in RNA capture, or reference mapping bias of allele-specific reads may limit the resolution at which the correct angle can be calculated, our computation provides a biologically interpretable measure of proportion *cis*. We found several genes in Barr et al. (2023) exhibiting high variance in proportion *cis* (Fig. 4c), and identified interesting differences between cell types (Supplementary Figs. 8–11), perhaps relating to cell cycle status or developmental stage of different cell types. For example, *Mageh1* codes for a member of a family of proteins that mediate apoptosis, cell growth, and cell cycle (Lee and Potts 2017; Shin et al. 2021): the difference in proportion *cis* across the assayed cell types could indicate different regulatory strategies at different stages of cell cycle, implicating it as an interesting candidate for follow-up experimental validation to explore this question. *Dnmt1* encodes DNA methyltransferase 1, a protein that copies the methylation patterns from template DNA strands onto newly synthesized strands during cell replication (Kikuchi et al. 2022): its variation in proportion *cis* across cell types may be evidence of varying regulation between actively proliferating versus quiescent cells, or cell types at different stages of development. These examples show that an analysis of the variation in proportion *cis* across cell types allows the identification of patterns (and the genes displaying them) beyond considering classifications into discrete groups (e.g. *cis* or *trans*).

**Fig. 4.**
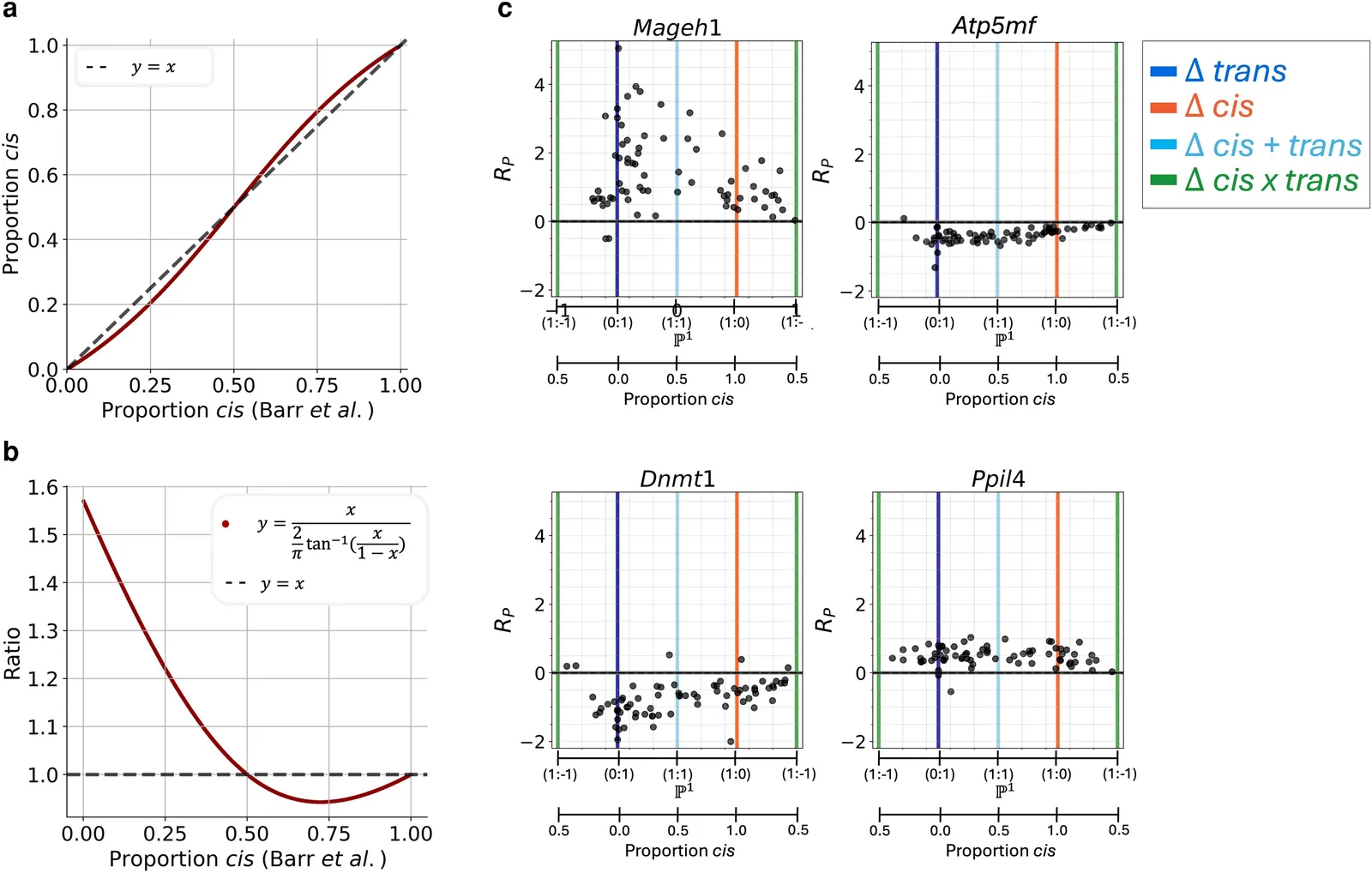
In addition to broad regulatory categories, genes can display subtle differences in their proportion of *cis* regulation. a) Comparison of slope (as previously defined in Barr et al. 2023, *x*-axis) and angle (*y*-axis) for determining proportion *cis*. b) Ratio of slope and angle determination of proportion *cis* (dark red line, dashed line displays equality). Note that at small values of proportion *cis*, the slope can be over 50% the determined angle. c) Gene expression data from Barr et al. (2023) (human–chimpanzee crossed cell lines) were used to determine cell type-specific proportion *cis*. These four genes display high variance in proportion *cis* across cell types (black points are determined proportion *cis* in n=72 cell types).

## Discussion

The use of crosses between homozygous strains to identify the nature of differential regulation is a powerful tool for genetics studies that is particularly relevant now that single-cell RNA-seq can be used for cell type resolution. Moreover, while original studies were limited to a handful of genes, genome-wide single-cell RNA-seq assays can complement genome-wide expression Quantitative Trait Locus (eQTL) studies.

Geometric considerations show that parental and hybrid allele ratios are most naturally interpreted after applying a linear transformation which separates cis and trans contributions onto independent axes. In this transformed space, the corresponding hypothesis tests arise directly from the geometry and provide a principled basis for classifying the regulatory sources of parental expression differences. While we have focused on explaining differences between parents that fall into five categories (conserved, *cis*, trans, *cis + trans*, *cis × trans*), our approach can be extended to finer classifications such as in Tsouris et al. (2024).

We note that regulatory assignments in this hypothesis-testing framework should be interpreted as statistical classifications rather than definitive causal decompositions. For example, a gene assigned to the *cis* category has evidence for a difference in cis regulation between the parental strains and lacks sufficient evidence to reject the null hypothesis of no trans effect; this does not prove that there is no difference in trans regulation, and similarly for *trans* assignment.

We also note that in the hypothesis-testing framework, referring to a gene as having differences in expression explained by *cis* or *trans* is technically incorrect. This is because the rejection of the *cis* null hypothesis only shows that the difference in gene expression is not due solely to *cis* regulation. This does not mean that the difference in gene expression can or should be attributed solely to *trans*. The same is the case for rejecting the *trans* hypothesis. In Fig. 2, our coloring of genes as cis or *trans* is therefore not precise, but we have done so to facilitate comparisons to previous work.

One strength of the GLM framework is that it allows F1 hybrid expression to be modeled under alternative assumptions about how parental trans environments combine in the hybrid. Although we recommend the log-additive model as the primary genome-wide screen, because we expect that model to be appropriate for most genes, there are many examples of hybrid expression that are nonadditive, including dominant, overdominant, or transgressive (Shahzad et al. 2020; Wang et al. 2022; Cui et al. 2023), thus as a complementary analyses one can fit alternative models to identify genes that may be missed under the log-additive assumption to determine whether some genes are better explained by a nonadditive model of F1 trans regulation.

Our single sample statistical tests depend on the assumption that read counts are binomially distributed, which is standard in the absence of biological replicates. However, when replicate samples are available, we can better assess the extent of technical variation and use GLMs. Although our results are shown using commonly applied estimation techniques for negative binomially distributed counts (Love et al. 2014), we also measure the extent to which differing estimates of variation change the results (Supplementary Material) (Robinson et al. 2010; Love et al. 2014).

Simulations indicate that reliable cis/trans inference generally requires at least six biological replicates per group, with eight replicates providing a more stable practical minimum across expression and dispersion settings (see Supplementary Simulation Figures). Effects of roughly two-fold or larger were detected with good power in many moderate- to high-expression settings, whereas smaller effects, low expression, high dispersion, or low ASE-informative read fractions required substantially more replication and remained difficult, especially for trans effects. Compared with the traditional three-test ratio-based framework, XgeneR showed essentially equivalent power and ROC/AUC for detecting cis and trans components, modestly improved overall regulatory-class assignment, and the additional advantage of estimating cis and trans effect sizes directly within a unified GLM framework. These results suggest that replication and read informativeness are critical design considerations for cis/trans studies, and that weak regulatory effects should be interpreted cautiously in small experiments.

Finally, we note that our framework is general and can be applied to more complex experimental designs, regulatory hypotheses, and phenotypes other than gene expression. For example, the versatility of our GLM framework allows modeling of *cis* or *trans* differences in specific conditions and in interactions between specific conditions. In terms of additional phenotypes, with single-cell RNA sequence data, our approach could be used in conjunction with methods such as Gorin et al. (2023) and Carilli et al. (2024) to assess the regulation mechanisms underlying differences in biophysical aspects of gene transcription, splicing, and degradation. Such extensions will be particularly interesting to explore in conjunction with complementary modalities (Irastorza-Azcarate et al. 2025; Ma et al. 2026).

## Supplementary Material

iyag228_Supplementary_Data

iyag228_Peer_Review_History

## Data Availability

All analyzed data can be downloaded via scripts and notebooks available at https://github.com/pachterlab/HCP_2024.

## Code availability

All code to download and analyze data, as well as generate the main and supplementary figures is available at https://github.com/pachterlab/HCP_2024, as well as an R package (*XgeneR*) and a Python package (*XgenePy*) to implement the multisample tests which are available at https://github.com/pachterlab/XgeneR and https://github.com/pachterlab/XgenePy, respectively.

Supplemental material available at GENETICS online.
