# Supplementary material for: Estimating cis and trans contributions to differences in gene regulation
Source: Genetics. 2026 Sep 11;234(3):iyag228. doi: 10.1093/genetics/iyag228 (PMC13732733; doi:10.1093/genetics/iyag228)
Supplement: Supplementary file 1 [file iyag228_peer_review_history.pdf]

GENETICS

Estimating cis and trans contributions to differences in gene regulation

Ingileif Hallgrimsdottir, Maria Carilli, and Lior Pachter

NOTE: The reviews and decision letters are unedited and appear as submitted by the reviewers.
In extremely rare instances and as determined by a Senior Editor or the EIC, portions of a review may be redacted. If a review is signed, the reviewer has agreed to no longer remain anonymous.
The review history appears in chronological order.

Review Timeline:

Submission Date:	2026-03-24
Editorial Decision:	2026-04-18
Revision Received:	2026-06-11
Accepted:	2026-06-27

April 18, 2026
RE: GENETICS-2026-309262

Dear Dr. Hallgrimsdottir:

I am pleased to accept your manuscript titled "Estimating cis and trans contributions to differences in gene regulation" for publication in GENETICS, pending minor revision.

Please submit your revision along with a brief description of how you modified the manuscript in response to the reviewers' concerns and suggestions (which can be viewed at the bottom of this email. I expect you should be able to submit a revised manuscript within 30 days. A suitably revised manuscript will be acceptable for publication; I don't expect to send it out for review.

Prior to uploading your revised manuscript please format it according to GENETICS style and ensure you have all required elements. Author Guidelines are at https://academic.oup.com/genetics/pages/author-guidelines#section-12. These guidelines have been updated with new requirements and your careful attention is required to avoid delays at the source file stage post-acceptance.

Please include a Data Availability Statement in your article under the heading "Data availability," before the Acknowledgements section. See https://academic.oup.com/genetics/pages/author-guidelines#section-12-11 for details. The DAS should include the accession numbers or DOIs of any data you have placed in public repositories, describe supplemental material, include applicable IRB numbers, and may include specifications for how to properly acknowledge or cite the data.

When revising the ms., please make an effort to shorten it, because that almost always improves a manuscript. We urge authors to heed the advice of Strunk and White: "omit needless words"1. Follow this link to submit the revised manuscript: Link Not Available

Thank you for submitting this story to Genetics.

Sincerely,

Yun Li
Associate Editor
GENETICS

Approved by:
Hongyu Zhao
Senior Editor
GENETICS

Reviewer comments:
Reviewer #1 :

A detailed review report is provided in the attached document. In summary, while the manuscript presents a mathematically rigorous and solid framework, I have outlined several key recommendations regarding statistical validation via simulations, biological interpretation, and software availability. Please refer to the attachment for the comprehensive comments.

Reviewer #2 :

Prior methods for classifying cis and trans regulatory differences have treated the cis and trans-regulation asymmetrically, either by working in the untransformed coordinate space (Tsouris et al.) or by fitting data across separate statistical tests (Ballinger et al.). This paper addresses both problems, and its reanalysis of previously published datasets identified substantially more cis-

regulated genes and fewer trans-only genes and has shifted conclusions consistently towards larger roles for cis regulation than previously concluded.

For multi-replicate data sets, they introduce a unified negative binomial GLM that jointly models parents and hybrids across multiple conditions. By incorporating organs and temperature as additional factors to the experimental design, this framework helped identified condition-specific regulatory differences that separate models would otherwise miss, proving the framework's added values to previous methods.

The human-chimpanzee and yeast single-replicate analyses read more as methodological comparisons than new gene-level discoveries. Some discussion of the implications of the results in Figs 2 and 4 (beyond the numbers of genes per category), similar to the authors' discussion of results in Fig 3, would be helpful. In addition, can the authors provide any insight into why the four genes highlighted in Fig 4c show variability in their cis proportion? If they are expressed at very low levels in some cell types, would that lead to greater measurement error and a bias in the cis/trans classification? In particular, Bloc1s1 has an odd pattern with many points along the vertical line at zero cis proportion.

The Barr et al. dataset is only analyzed with binomial-based framework from linear transformation of published R_H and R_P log2 fold changes, without fitting the GLM to per-replicate raw counts provided in GSE201516. Given that the dataset includes 3 replicates per parent and per hybrid across 72 cell types, fitting the GLM on the dataset from Barr et al. with cell type, germ layer or functional lineage as additional factor (analogous to the reanalysis of Ballinger data) could potentially reveal cell type, germ layer or functional-specific regulatory divergence between the species in addition to generally picturing the cross-cell-type variance of such potential candidates like Mageh1 and Bloc1s1 in Figure 4C.

In addition, the paper recommends experimentalists to include enough samples (for example, 6 replicates per group in Ballinger data) in the design for heuristic-free variance estimation. It could be helpful if the paper could offer a power analysis or a recommendation of sample sizes for which their GLM framework would be most reliable.

Finally, the three trans models (log-additive, dominant and free) could be applicable to previously published datasets where the model choice has a direct biological meaning. For example, a likelihood ratio test comparing dominance versus log-additive fits to known imprinted gene dataset could potentially serve as a validation of the model's ability to distinguish between trans-regulatory mechanisms. Similarly, comparing the free versus dominant model fits to hybrid datasets involving reciprocal crosses could potentially help quantify how much of the trans effect is dependent on the direction of the cross.

Minor:
1. typo line 83: "this our case"
2. Gmnn in is Fig 3d but Hapln4 is in the matching position of Fig 3e-is this a mistake?
3. Why is Map3k14 given as an example of environment-specific, when signal is much stronger for organ-env interaction?
4. Likewise why is Mus81 given as an example of environment-specific, when signal is much stronger for organ and for organ-env interaction?

Reviewer #3 :

This manuscript addresses a central question in regulatory genetics: how to determine whether differences in gene expression between two homozygous strains or species are driven by cis-regulatory effects, trans-regulatory effects, or combinations of the two. The study presents a coordinate-system-based and hypothesis-testing framework for classifying regulatory divergence from parental and hybrid expression data, providing a clearer geometric interpretation of the problem and an explicit classification of genes based on statistical test results.

One of the major strengths of the study is its geometric framework, which offers an intuitive structure for understanding regulatory assignments, while the statistical testing approach provides a more rigorous basis for classification than many currently used strategies. The manuscript is further strengthened by its application across multiple biological systems. The analyses in yeast, mouse, and human-chimpanzee datasets demonstrate that the framework is relevant across diverse experimental settings.

Overall, I view this work as a significant methodological improvement over current approaches for cis/trans inference. It offers a more comprehensive, statistically grounded, and extensible framework for studying regulatory divergence, and it should be a valuable resource for the field. I support publication of this manuscript.

Associate Editor comments:

Review of: ”Estimating cis and trans contributions to differences in gene regulation”

Summary of the Work

The manuscript provides a mathematically rigorous framework for distinguishing *cis* and *trans* regulatory modalities. By transforming the log-fold change coordinates into an orthogonal space and employing an associated hypothesis testing framework (including a GLM approach for replicated designs), the authors address long-standing ambiguities in the metric space (e.g., calculating proportion *cis* using angle rather than slope). The methodological foundation is solid, and the reanalysis of multiple published datasets (yeast, human-chimpanzee, and mouse crosses) illustrates the practical application of the approach. To further strengthen the manuscript before publication, I recommend addressing several key aspects regarding statistical validation (via simulations), biological interpretation, practical impact, and software availability.

Review Comments

1. **Necessity of Simulation Studies for Validation:** The proposed statistical framework yields substantially more *cis* assignments than previous approaches in both the single-sample and replicated settings (e.g., 17,112 vs. 2,804 in yeast, and 877 vs. 478 in BAT/cold mouse samples). Because these assignments depend on hypothesis-testing power, and failure to reject the alternative null does not imply acceptance of a purely *cis* or purely *trans* model, drawing absolute conclusions from real-world data lacking a ground truth is challenging. To fully validate this promising method, simulation studies with known ground truths would substantially strengthen the paper. Generating simulated read counts with known *cis* and *trans* effect sizes under varying levels of dispersion would explicitly benchmark the Statistical Power, Receiver Operating Characteristic (ROC), and empirical False Discovery Rate (FDR).

2. **Over-interpretation of Categorical Labels:** As the authors candidly acknowledge in the Discussion, the regulatory labels (e.g., *cis*, *trans*) are based on rejection or failure-to-reject patterns rather than proof of purely *cis* or purely *trans* regulation. However, the manuscript subsequently compares large numbers of genes assigned to these absolute categories, which may lead readers to over-interpret these classifications. Because this interpretive caveat is fundamental to the framework, it should be emphasized earlier and more prominently in the text, particularly where headline differences in the numbers of *cis*- and *trans*-assigned genes are discussed.

3. **Biological Justification for Trans Hypotheses:** The manuscript introduces three *trans* hypotheses within the GLM framework—`log-additive`, `dominant`, and `free`—and compares their fitted weights, significance values, and regulatory assignments empirically (e.g., in Supp. Figs. 4–6). The paper would be greatly strengthened by a clearer biological interpretation of what kinds of molecular mechanisms in F1 hybrids would motivate each mathematical model. Clarifying when one *trans* hypothesis should be preferred over another from a mechanistic standpoint (e.g., transcription factor dynamics or haploinsufficiency) would make the framework much more accessible to experimental geneticists.

4. **Practical Impact of Geometric Correction vs. Technical Noise:** The geometric correction from slope to angle is mathematically well-motivated, and the manuscript notes that the maximum absolute difference is 0.045, while the relative difference can be substantial when proportion *cis* is small. It would be helpful to clarify in what practical regimes this correction materially changes biological interpretation—for example, gene ranking, threshold-based summaries, or comparisons across cell types—relative to typical technical and inferential uncertainty in ASE measurements (such as reference mapping bias).

5. **Code and Software Availability:** The manuscript states that the analyses were implemented using the `XgeneR` package with edgeR, but I did not see an explicit code/data availability statement or a public link to `XgeneR` in the current draft. For transparency and reproducibility, the authors should provide a repository or archived release for `XgeneR` and the custom scripts used to reproduce the reported re-analyses; if these resources are already public, they should be explicitly cited/linked in the manuscript.

Reviewer 1

A detailed review report is provided in the attached document. In summary, while the manuscript presents a mathematically rigorous and solid framework, I have outlined several key recommendations regarding statistical validation via simulations, biological interpretation, and software availability. Please refer to the attachment for the comprehensive comments.

Summary of the Work

The manuscript provides a mathematically rigorous framework for distinguishing *cis* and *trans* regulatory modalities. By transforming the log-fold change coordinates into an orthogonal space and employing an associated hypothesis testing framework (including a GLM approach for replicated designs), the authors address long-standing ambiguities in the metric space (e.g., calculating proportion *cis* using angle rather than slope). The methodological foundation is solid, and the re- analysis of multiple published datasets (yeast, human-chimpanzee, and mouse crosses) illustrates the practical application of the approach. To further strengthen the manuscript before publication, I recommend addressing several key aspects regarding statistical validation (via simulations), biological interpretation, practical impact, and software availability.

Review Comments

1. **Necessity of Simulation Studies for Validation:** The proposed statistical framework yields substantially more *cis* assignments than previous approaches in both the single-sample and replicated settings (e.g., 17,112 vs. 2,804 in yeast, and 877 vs. 478 in BAT/cold mouse samples). Because these assignments depend on hypothesis-testing power, and failure to reject the alternative null does not imply acceptance of a purely *cis* or purely *trans* model, drawing absolute conclusions from real-world data lacking a ground truth is challenging. To fully validate this promising method, simulation studies with known ground truths would substantially strengthen the paper. Generating simulated read counts with known *cis* and *trans* effect sizes under varying levels of dispersion would explicitly benchmark the Statistical Power, Receiver Operating Characteristic (ROC), and empirical False Discovery Rate (FDR).

We thank the reviewer for this request for validation. We have performed an extensive simulation study that greatly strengthens the quality of our paper. In addition, it caused us to reparameterize the *trans* model design matrices for more useful interpretation of learned weights and more stable estimates of effect sizes. We point the reviewer to the added Section "Simulation Study" in Methods (see page 5, line 58 in the tracked manuscript), an added paragraph in Discussion (see page 8 line 46 in the tracked manuscript) and also two new supplements, one with a description of the simulation set up and a supplement with figures

based on the simulation study. The code for running the simulation study has been added to the github repository for the paper.

2. **Over-interpretation of Categorical Labels:** As the authors candidly acknowledge in the Discussion, the regulatory labels (e.g., *cis*, *trans*) are based on rejection or failure-to-reject patterns rather than proof of purely *cis* or purely *trans* regulation. However, the manuscript subsequently compares large numbers of genes assigned to these absolute categories, which may lead readers to over-interpret these classifications. Because this interpretive caveat is fundamental to the framework, it should be emphasized earlier and more prominently in the text, particularly where headline differences in the numbers of *cis*- and *trans*-assigned genes are discussed.

Indeed, as the reviewer points out, one of the strengths of our proposed geometry and hypothesis testing framework is the nuance of failure to reject a hypothesis as evidence of *cis* or *trans* regulation, as well as calculation of proportion *cis* rather than discrete regulatory assignments. We have updated the text to more faithfully reflect these nuances. We added to the text of the Results "Statistics" section (page 6, line 26 in the tracked manuscript) "As explained in the Materials and Methods, the failure to reject the null hypotheses "no *cis*" or "no *trans*" do not imply the acceptance of those nulls; our classifications reflect the statistical power of the experiment to reject the absence of *cis* or *trans* regulation, which decreases with smaller sample size and weaker effect sizes. We explore this with ground-truth simulations across a range of sample and effect sizes (see Simulation Supplement Figures), but discuss discrete classifications for consistency with previous publications." We also added text to help clarify the interpretation of regulatory assignments following the discussion of yeast crosses in the Results "Statistics" section (page 6, line 73 in tracked manuscript): "While these discrete classifications based on the outcomes of statistical tests can be compared, there is a range of proportions *cis* for genes within a classification (Fig. 2A and 2C). Notable, the *trans* classifications of Tsouris *et al.* extend up to a calculated proportion *cis* of almost 1 (Fig. 2A), while our assignments are restricted to a region closer to the *trans* axis, explaining the possible over-assignment of *trans* regulation in the original study."

3. **Biological Justification for Trans Hypotheses:** The manuscript introduces three *trans* hy- potheses within the GLM framework—log-additive, dominant, and free—and com- pares their fitted weights, significance values, and regulatory assignments empirically (e.g., in Supp. Figs. 4–6). The paper would be greatly strengthened by a clearer biological inter- pretation of what kinds of molecular mechanisms in F1 hybrids would motivate each mathe- matical model. Clarifying when one *trans* hypothesis should be preferred over another from a mechanistic standpoint (e.g., transcription factor dynamics or haploinsufficiency) would make the framework much more accessible to experimental geneticists.

Biological interpretation of the three proposed models have been included in the Discussion (page 8, line 20 in tracked manuscript), with cited studies that highlight the relevance of our proposed models. We have further updated the parameterization of the design matrices so that the learned weights are more directly biological interpretable (though mathematically equivalent to our former proposed design matrices): the intercept is at the geometric mean of parental

expression, the weight for *cis* captures the difference between the parents attributable to *cis* regulation, and the weight for *trans* captures the parental expression difference attributable to *trans* regulation (page 2, line 106 and page 3 line 88 in tracked manuscript)

4. **Practical Impact of Geometric Correction vs. Technical Noise:** The geometric correction from slope to angle is mathematically well-motivated, and the manuscript notes that the maximum absolute difference is 0.045, while the relative difference can be substantial when proportion *cis* is small. It would be helpful to clarify in what practical regimes this correction materially changes biological interpretation—for example, gene ranking, threshold-based summaries, or comparisons across cell types—relative to typical technical and inferential uncertainty in ASE measurements (such as reference mapping bias).

We thank the reviewer for asking for clarification on the practical consequences of the slope vs. angle proportion *cis* calculation. We have added a sentence to the main text to clarify for the reader that while this is the geometrically correct way to calculate proportion *cis*, uncertainty in ASE mapping and experimental noise in data collection may be of greater magnitude than this theoretically correct adjustment: "While uncertainty in fold-change estimates due to low sample or effect sizes, experimental or technical noise in RNA capture, or reference mapping bias of allele specific reads may limit the resolution at which the correct angle can be calculated, our computation provides a biologically interpretable measure of proportion *cis*." (page 7, line 73 in tracked manuscript)

5. **Code and Software Availability:** The manuscript states that the analyses were implemented using the XgeneR package with edgeR, but I did not see an explicit code/data availability statement or a public link to XgeneR in the current draft. For transparency and reproducibility, the authors should provide a repository or archived release for XgeneR and the custom scripts used to reproduce the reported re-analyses; if these resources are already public, they should be explicitly cited/linked in the manuscript.

We thank the reviewer for catching this: our preprint includes the Code/Software Availability statement, but our initial submission to GENETICS did not. We have now added the section with links to GitHub repositories for the manuscript computation and figure generation as well as R and Python versions of the GLM software for multi-sample hypothesis testing (XgeneR and XgenePy) (page 8, line 78 in tracked manuscript).

Reviewer 2

Prior methods for classifying cis and trans regulatory differences have treated the cis and trans-regulation asymmetrically, either by working in the untransformed coordinate space (Tsouris et al.) or by fitting data across separate statistical tests (Ballinger et al.).

This paper addresses both problems, and its reanalysis of previously published datasets identified substantially more cis-regulated genes and fewer trans-only genes and has shifted conclusions consistently towards larger roles for cis regulation than previously concluded.

For multi-replicate data sets, they introduce a unified negative binomial GLM that jointly models parents and hybrids across multiple conditions. By incorporating organs and temperature as additional factors to the experimental design, this framework helped identified condition-specific regulatory differences that separate models would otherwise miss, proving the framework's added values to previous methods.

The human-chimpanzee and yeast single-replicate analyses read more as methodological comparisons than new gene-level discoveries. Some discussion of the implications of the results in Figs 2 and 4 (beyond the numbers of genes per category), similar to the authors' discussion of results in Fig 3, would be helpful.

The reviewer highlights an important point that biological interpretation of the differences we report would strengthen our presentation of the method. For the single-replicate yeast samples, we have thus added the following text to the Results: ”While it has been suggested that *trans* regulation accounts for more variation in expression within species and *cis* regulation accounts for interspecific divergence (Signor and Nuzhdin 2018), our results indicate that *cis* regulation, with still fewer assigned instances than *trans*, may play a larger role than previously assessed.” (page 6, line 80 in tracked manuscript).

For the human-chimpanzee crosses, we have added the following discussion of our method’s biological relevance (page 7, line 78 in tracked manuscript): ”We found several genes in (Barr *et al.* 2023) exhibiting high variance in proportion *cis* (Fig.~4C), and identified interesting differences between cell types (Supp. Fig.~8 -- 11), perhaps relating to cell cycle status or developmental stage of different cell types. For example, *Mageh1* codes for a member a family of proteins that mediate apoptosis, cell growth, and cell cycle (Shin *et al.* 2021, Lee and Potts 2017): the difference in proportion *cis* across the assayed cell types could indicate different regulatory strategies at different stages of cell cycle, implicating it as an interesting candidate for follow-up experimental validation to explore this question. *Dnmt1* encodes DNA methyltransferase 1, a protein that copies the methylation patterns from template DNA strands onto newly synthesized strands during cell replication (Kikuchi *et al.* 2022): its variation in proportion *cis* across cell types may be evidence of varying regulation between actively proliferating versus quiescent cells, or cell types at different stages of development. These examples show that an analysis of the variation in proportion *cis* across cell types allows the

identification of patterns (and the genes displaying them) beyond considering classifications into discrete groups (e.g., *cis* or *trans*).

In addition, can the authors provide any insight into why the four genes highlighted in Fig 4c show variability in their cis proportion? If they are expressed at very low levels in some cell types, would that lead to greater measurement error and a bias in the cis/trans classification? In particular, Bloc1s1 has an odd pattern with many points along the vertical line at zero cis proportion.

We thank the reviewer for highlighting the importance of interpreting the variability in *cis* proportion. While it is true that technical artifacts may lead to different calculations of *cis* proportion, we now added biological interpretation (copied from the manuscript in response to the previous request). As for *Bloc1s1,* we agree that the points along the vertical line of *trans* regulation may be indication of technical rather than biological noise (no hybrid expression in those cell types, for example), so we have replaced it with the gene *Atpfm5* which does not display this pattern. See Figure 4c.

The Barr et al. dataset is only analyzed with binomial-based framework from linear transformation of published R_H and R_P log2 fold changes, without fitting the GLM to per-replicate raw counts provided in GSE201516. Given that the dataset includes 3 replicates per parent and per hybrid across 72 cell types, fitting the GLM on the dataset from Barr et al. with cell type, germ layer or functional lineage as additional factor (analogous to the reanalysis of Ballinger data) could potentially reveal cell type, germ layer or functional-specific regulatory divergence between the species in addition to generally picturing the cross-cell-type variance of such potential candidates like Mageh1 and Bloc1s1 in Figure 4C.

While we agree with the reviewer that it would be extremely interesting to fit our proposed GLM framework to explore cell-type specific regulatory differences between humans and chimpanzees, we specifically chose not to recalculate fold changes to highlight the way proportion *cis* calculation varies with the original fold changes the authors provided. If we had first recalculated cell type specific fold changes using our GLM framework, the comparison of their reported proportion's *cis* and our own would not be valid.

That being said, we are extremely interested in using not only this human-chimp dataset, but the many existing similar datasets (Gokhman *et al.* 2021, Wang *et al.* 2025, Agoglia et al., 2021) for a comprehensive exploration of human-chimpanzee divergence at the resolution of cell type specific effects in future work.

In addition, the paper recommends experimentalists to include enough samples (for example, 6 replicates per group in Ballinger data) in the design for heuristic-free variance estimation. It could be helpful if the paper could offer a power analysis or a recommendation of sample sizes for which their GLM framework would be most reliable.

This is a very insightful comment, and led us to conduct an extensive simulation study with multiple replicates, levels of gene expression, and *cis* and *trans* effect sizes.

We point the reviewer to the added Section "Simulation Study" in Methods (see page 5, line 58 in the tracked manuscript), an added paragraph in Discussion (see page 8 line 46 in the tracked manuscript) and two new supplements, one with a description of the simulation set up and a supplement with figures based on the simulation study. The code for running the simulation study has been added to the github repository for the paper.

Finally, the three trans models (log-additive, dominant and free) could be applicable to previously published datasets where the model choice has a direct biological meaning. For example, a likelihood ratio test comparing dominance versus log-additive fits to known imprinted gene dataset could potentially serve as a validation of the model's ability to distinguish between trans-regulatory mechanisms. Similarly, comparing the free versus dominant model fits to hybrid datasets involving reciprocal crosses could potentially help quantify how much of the trans effect is dependent on the direction of the cross.

Again, we thank the reviewer for suggesting these exciting applications. For a biological study, we agree that such datasets as gene imprinting and reciprocal crosses would be excellent representatives of different *trans* regulatory mechanisms, we chose to use a dataset with replicates and different environments. We are, however, in active conversation with biological labs about generating reciprocal crosses in mouse strains with the requisite number of replicates for a confident application of our developed GLM.

Minor:

1. typo line 83: "this our case"

We thank the reader for catching this typo; we have corrected this in the text.

2. Gmnn in is Fig 3d but Hapln4 is in the matching position of Fig 3e-is this a mistake?

We thank the reviewer for catching this: as we have now updated the genes in response to the reviewer's next two comments, the genes in Fig 3e and d now appear in the correct order.

3. Why is Map3k14 given as an example of environment-specific, when signal is much stronger for organ-env interaction?
We thank the reviewer for careful observation of the expression values, and agree that a different example would show this signal more clearly. We have thus updated Figure 3 with new gene examples that more clearly display the regulatory patterns of organ specific regulation.

4. Likewise why is Mus81 given as an example of environment-specific, when signal is much stronger for organ and for organ-env interaction?
Again, we have updated Figure 3 with clearer examples. We have further added text to describe the classification of the two example genes with temperature/environment specific regulation. As environment specific *trans* regulatory effects with our updated design matrices (see comments on the simulation study) failed to reach significance, we chose genes with temperature specific *cis* regulation, one with overall *cis* in addition to temperature/environment specific *cis* regulatory differences, and another with only *cis* regulatory differences in a specific temperature/environment.

Reviewer 3

This manuscript addresses a central question in regulatory genetics: how to determine whether differences in gene expression between two homozygous strains or species are driven by cis-regulatory effects, trans-regulatory effects, or combinations of the two. The study presents a coordinate-system-based and hypothesis-testing framework for classifying regulatory divergence from parental and hybrid expression data, providing a clearer geometric interpretation of the problem and an explicit classification of genes based on statistical test results.

One of the major strengths of the study is its geometric framework, which offers an intuitive structure for understanding regulatory assignments, while the statistical testing approach provides a more rigorous basis for classification than many currently used strategies. The manuscript is further strengthened by its application across multiple biological systems. The analyses in yeast, mouse, and human-chimpanzee datasets demonstrate that the framework is relevant across diverse experimental settings.

Overall, I view this work as a significant methodological improvement over current approaches for cis/trans inference. It offers a more comprehensive, statistically grounded, and extensible framework for studying regulatory divergence, and it should be a valuable resource for the field. I support publication of this manuscript.

We thank the reviewer for their careful reading and assessment of our manuscript.

June 27, 2026
RE: GENETICS-2026-309262R1

Dr. Ingileif B. Hallgrimsdottir
California Institute of Technology Division of Biology and Biological Engineering
Biology and Biological Engineering
Room 236, Beckman Behavioral Biology Laboratory
Pasadena, California 91125

Dear Dr. Hallgrimsdottir:

Congratulations, your manuscript titled "Estimating cis and trans contributions to differences in gene regulation" is accepted for publication in GENETICS! Many thanks for submitting your research to the journal. Please address the minor suggestion from reviewer 1.

To Proceed to Publication:
1. Format your article according to GENETICS style: https://academic.oup.com/genetics/pages/author-guidelines

2. Ensure that you comply with data and community resource citation guidelines:
https://academic.oup.com/genetics/pages/author-guidelines#section-5-9-2

3. Upload your final files at https://genetics.msubmit.net

4. Add oupsupport@scipris.com and genetics.oup@novatechset.com (or the domains @scipris.com and @novatechset.com) to your email program's "safe senders" list. You will be contacted by both at various points during the production process.

Notes:
- Your currently-accepted manuscript (unedited, as submitted, reviewed, and accepted) will be published at GENETICS and deposited into PubMed as an Advance Access article. Notify sourcefiles@thegsajournals.org before signing your license if you do not wish to publish your article via Advance Access.

- We invite you to submit an original color figure related to your paper for consideration as cover art. Please note that submissions created with AI image generation tools are not eligible. Please email your submission to the editorial office or upload it with your final files. You can submit a small-sized image for evaluation, and if selected, the final image must be a TIFF file 2513px wide by 3263px high (8.375 by 10.875 inches; resolution of 600ppi). Please avoid graphs and small type.

- After files are sent to Oxford University Press we use SciPris to manage article licensing and payment. If you do not have a SciPris account, you will receive an email from no-reply@scipris.com to sign up to use Oxford University Press' author portal. After logging in, follow the online instructions to sign your license and arrange any payment due.

If you have any questions or encounter any problems while uploading your accepted manuscript files, please email the editorial office at sourcefiles@thegsajournals.org.

Sincerely,

Yun Li
Associate Editor
GENETICS

Approved by:
Hongyu Zhao
Senior Editor
GENETICS

Review comments (if applicable):

Reviewer #1 :

The revised manuscript is substantially improved. The authors have responded carefully to the main concerns raised in the previous round. I only have one minor suggestion, please see the attached file for more details.

Reviewer #2 :

The authors have addressed all of my concerns.

Reviewer #3 :

Thanks for improving the manuscript based on the reviewers' comments.

Review of: "Estimating cis and trans contributions to differences in gene regulation"

Assessment of the Revision

The revised manuscript is substantially improved. The authors have responded carefully to the main concerns raised in the previous round. In particular, the newly added simulation study is a meaningful addition rather than a superficial change: it evaluates the framework across replicate number, expression level, dispersion, ASE-informative read fraction, and effect size, and reports power, ROC/AUC, false-positive behavior, and classification accuracy. This directly addresses my main concern about validating the method in settings with known ground truth.

The authors have also clarified the interpretation of statistical regulatory labels, expanded the biological discussion of the *trans* models, revised several examples in response to the other reviewers, and added code/software availability information. Overall, I find that the main scientific concerns from the first round have been addressed, and the remaining point is minor.

Minor Comment

The only remaining point I would suggest clarifying is the scope of the simulation-based practical recommendations. The statement that reliable *cis*/*trans* inference generally requires at least 6 biological replicates per group, with 8 being a more stable practical minimum, is useful, but it should be explicitly framed as conditional on the simulated settings. For example, the simulations fix reference bias at zero and use a fixed beta-binomial concentration, while the manuscript elsewhere notes that ASE mapping uncertainty and technical noise can affect inference. A brief sentence in the main text or simulation supplement noting these assumptions, and possibly clarifying the FDR threshold used in the simulation summaries relative to the empirical analyses, would be sufficient.
