# Supplementary material for: Estimating cis and trans contributions to differences in gene regulation
Source: Genetics. 2026 Sep 11;234(3):iyag228. doi: 10.1093/genetics/iyag228 (PMC13732733; doi:10.1093/genetics/iyag228)
Supplement: Supplementary file 2 [file iyag228_supplementary_data.zip › Supplemental_Material_GENETICS-2026-309262.pdf]

Supplementary Material for:
Estimating cis and trans contributions to differences in gene regulation

Ingileif B. Hallgrímsdóttir[1,*], Maria Carilli[1] and Lior Pachter[1,2]

[1]Division of Biology and Biological Engineering, California Institute of Technology, Pasadena, CA 91125, USA
[2]Department of Computing and Mathematical Sciences, California Institute of Technology, Pasadena, CA 91125, USA

[*]Corresponding author: Ingileif Hallgrimsdottir (ingileifcaltech.edu)

Abstract

We describe a coordinate system and associated hypothesis testing framework for determining whether *cis* or *trans* regulation is responsible for differences in gene expression between two homozygous strains or species. We apply our framework to data from single replicate studies on yeast strains and human-chimpanzee hybrid cells, as well as to data from a mouse study with replicates, showing marked differences between our gene regulatory assignments and those previously reported. We also show how our multi-sample framework can determine the context dependency of *cis* and *trans* effects as well as explicitly model different hypotheses regarding the underlying mechanism of *trans* regulation.

Keywords: gene regulation; statistical method; RNA-seq

Supplementary tables

$R_P - R_H$	R_P	R_H	**Regulatory assignment**
0	0	0	conserved
0	+	+	Δ *cis* +
0	-	-	Δ *cis* -
+	+	0	Δ *trans* +
+	$R_P > R_H$	+	Δ *cis* + *trans* +
+	$R_P > R_H$	-	Δ *cis* × *trans* +
-	-	0	Δ *trans* -
-	$R_P < R_H$	+	Δ *cis* × *trans* -
-	$R_P < R_H$	-	Δ *cis* + *trans* -

Supp. Table 1: Geometric assignments of gene regulatory differences. Assignments of gene expression differences to *cis, trans, cis* + *trans* or *cis* × *trans* regulation can be made based on the sign of values of $R_P - R_H$ (log2 of parental fold change minus log2 of hybrid fold change) and R_H (log2 of hybrid fold change). Possible value pairs and the corresponding regulatory assignments are enumerated above.

Supplementary figures

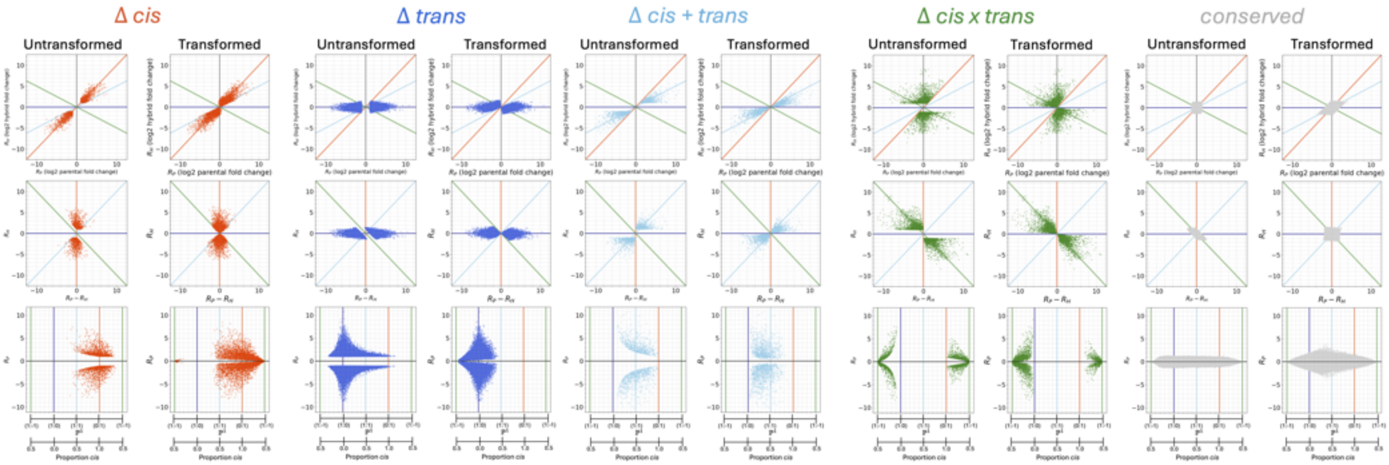

Supp. Fig. 1: The points in Figures 2A and 2C separated by regulatory assignment (color). Across all assignments, n = 285,777 gene-cross combinations from 179 unique yeast parent crosses are shown (gene expression data from Tsouris et al., 2024.)

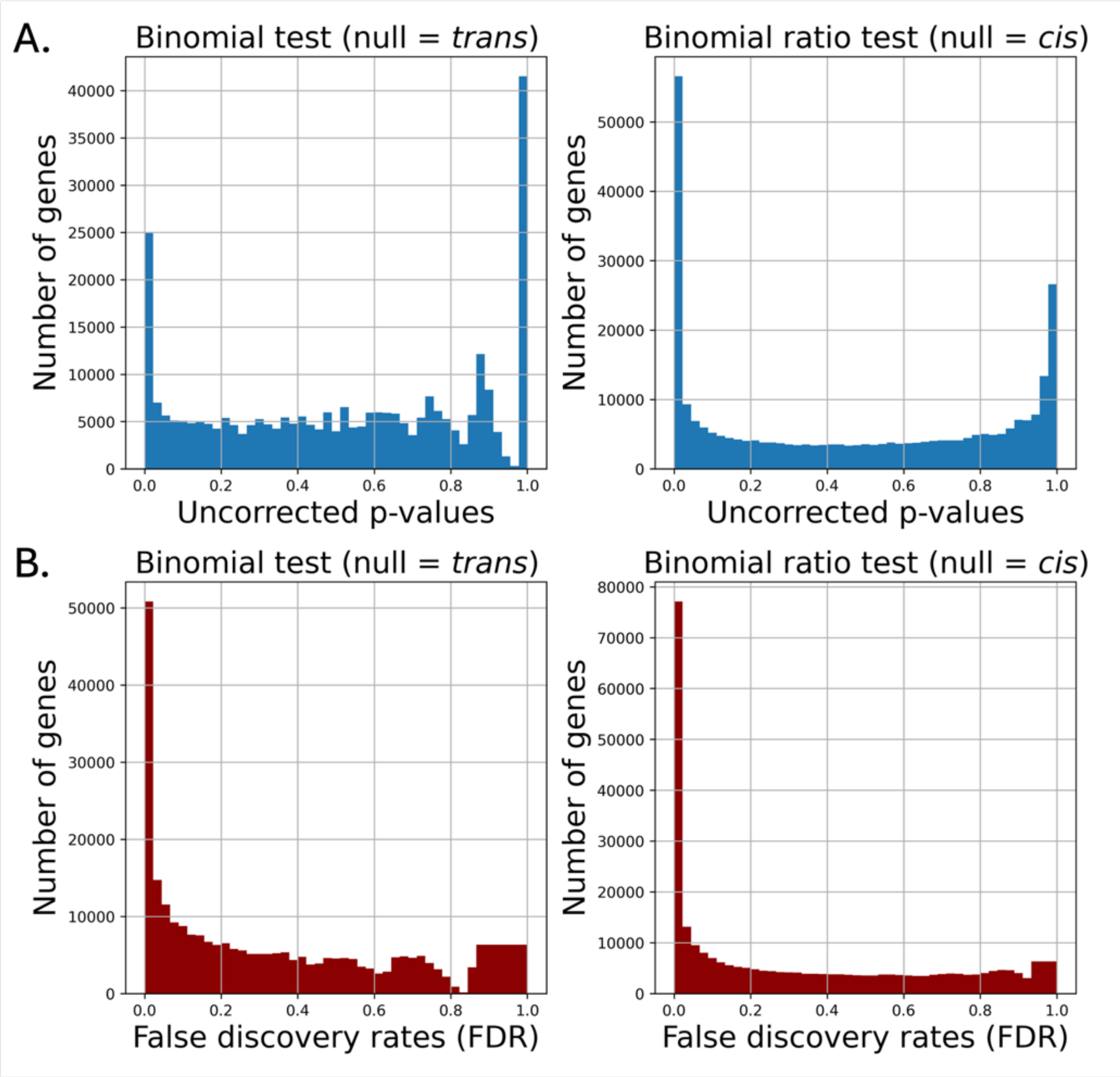

Supp. Fig. 2: A) Distribution of p-values for the n = 285,777 yeast gene-cross combinations (Tsouris et al., 2024). B) Distribution of false discovery rates for the same calculated from raw p-values using the Benjamini-Hochberg procedure (Benjamini and Hochberg, 1995).

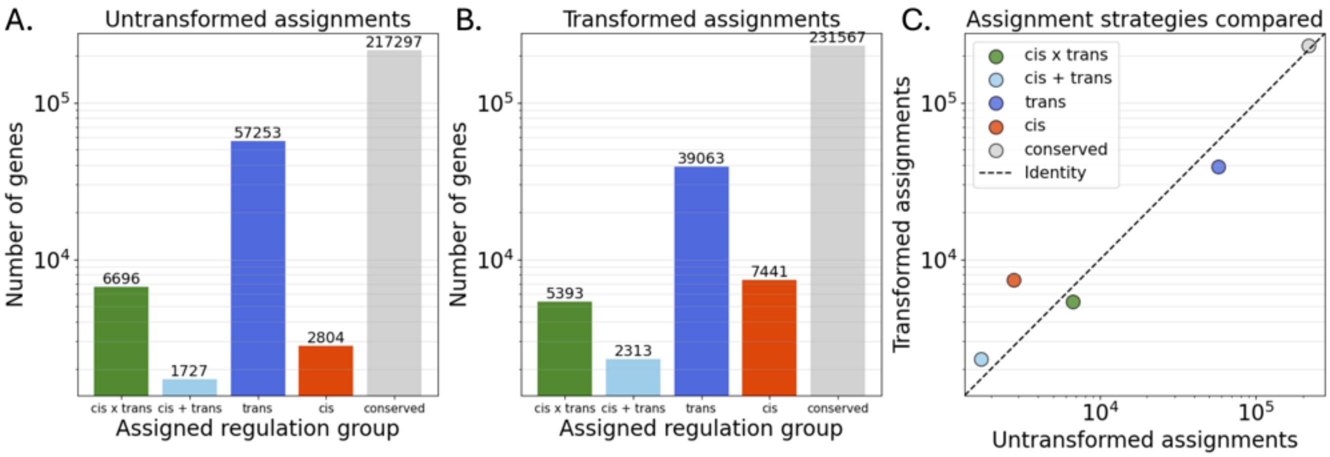

Supp. Fig. 3: Comparison of regulatory assignments for n = 285,777 gene-cross combinations in yeast cross experiments (Tsouris et al., 2024). A) The number of genes assigned to designated regulatory categories from Tsouris et al., 2024, and B) our method's assignments. C) Comparison of the number of genes per regulatory category between the previous method (untransformed assignments, x-axis) and our hypothesis testing framework (transformed assignments, y-axis).

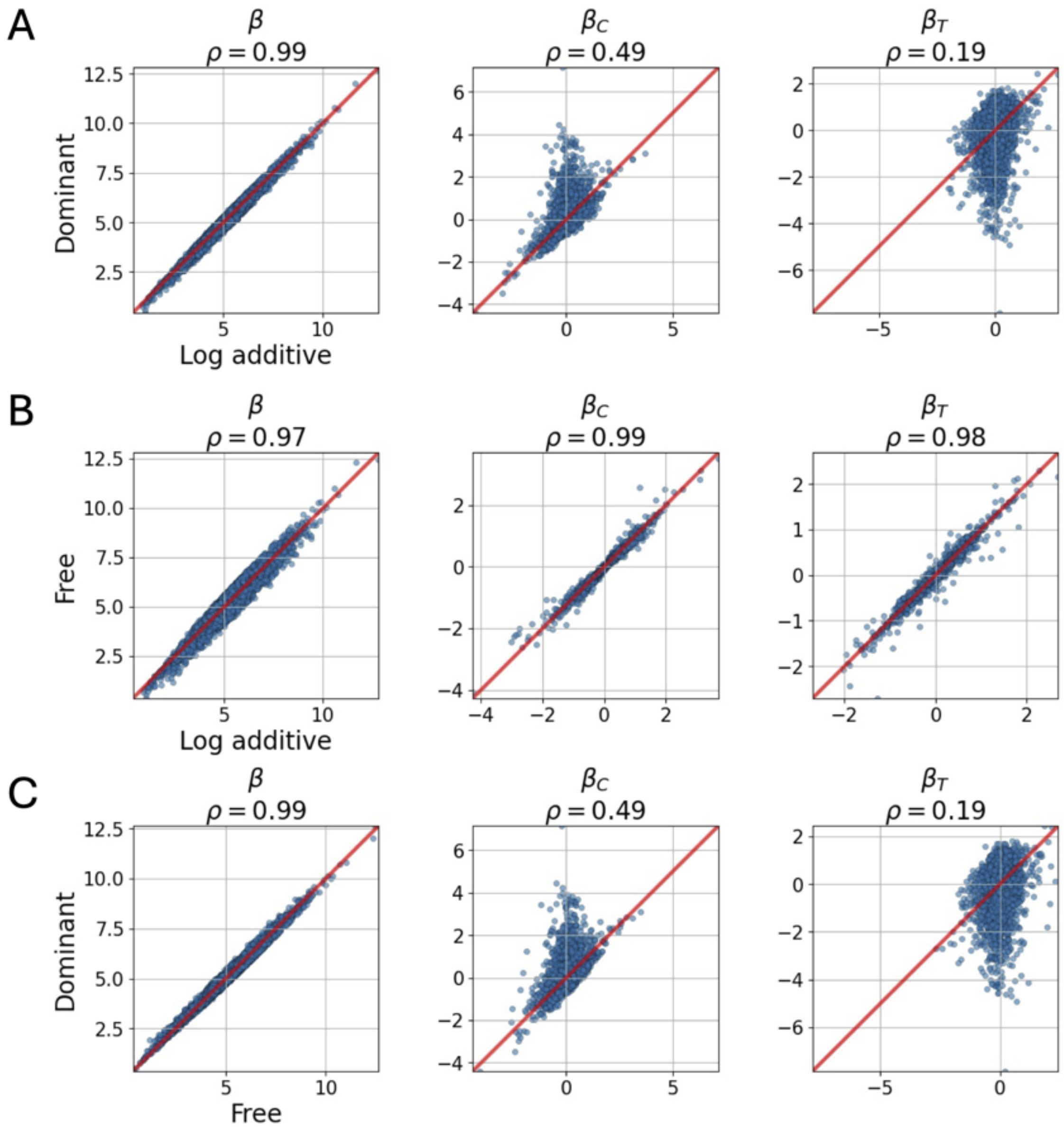

Supp. Fig. 4: Comparison of generalized linear model weights fit to BAT tissue samples from mice reared in cold tissues (n = 6 parental strain one, n = 6 parental strain two, n = 6 hybrid crosses) under different model hypotheses (see Methods). β is the intercept shared between all samples, β_C captures the *cis* effect unique to the non-reference allele, and β_T captures the *trans* effect common to hybrids and the non-reference allele (see Methods). Pearson correlation coefficients (ρ) are displayed for each subplot. A) Dominant vs. log additive model, B) free vs. log additive model, and C) dominant vs. free model. Data from Ballinger et al., 2023.

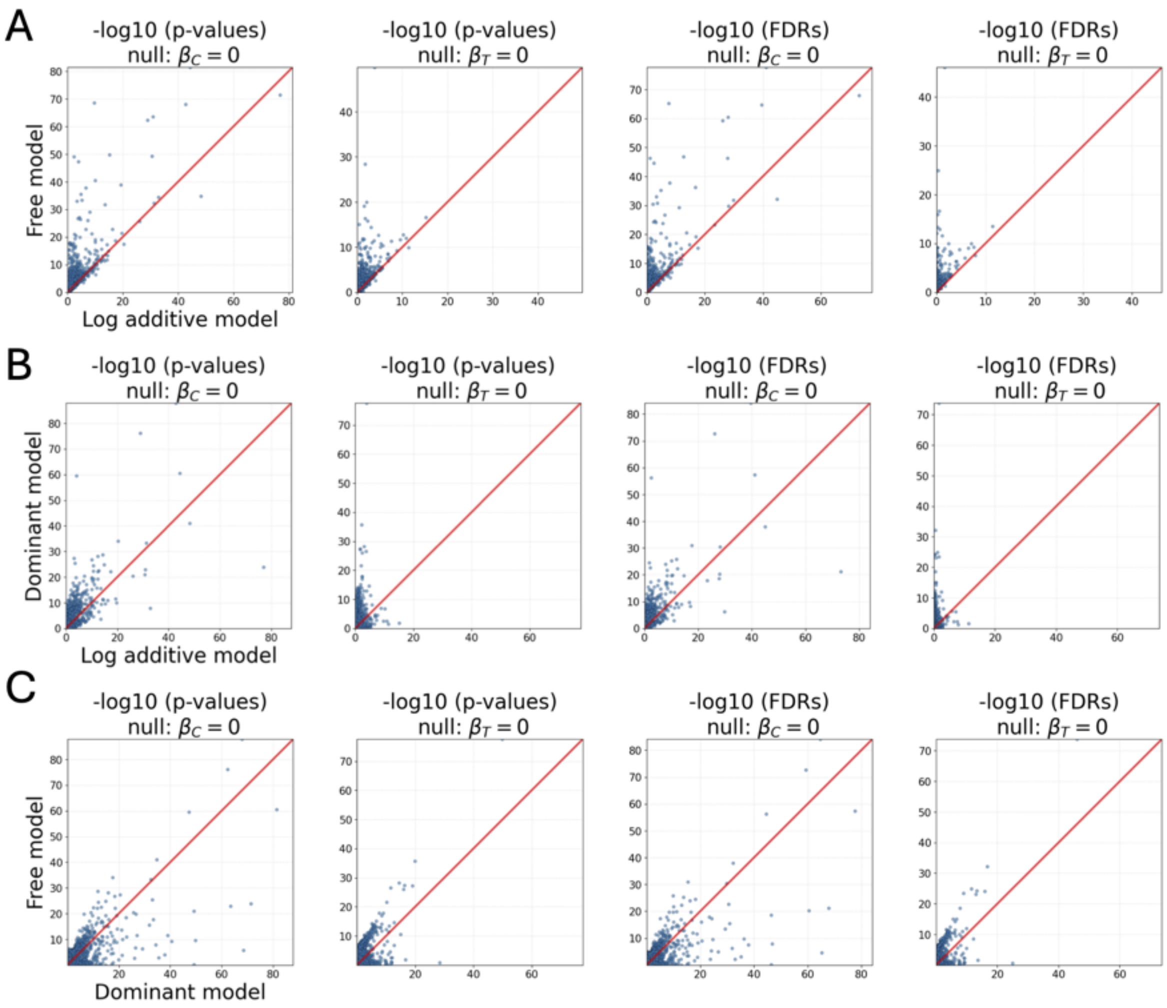

Supp. Fig. 5: Comparison of generalized linear model significance values, both raw p-values and Benjamini Hochberg corrected false discovery rates (Benjamini and Hochberg, 1995) for the null hypotheses of no *cis* ($\beta_C = 0$) and no *trans* ($\beta_T = 0$) regulation (see Methods). Comparisons of A) free and log additive models, B) dominant and log additive models, and C) free and log additive models. The models were fit to BAT tissue samples from male mice in reared in the cold (n = 6 parental strain one, n = 6 two, and n = 6 for hybrid crosses with n = 5,970 genes). Data from Ballinger et al., 2023.

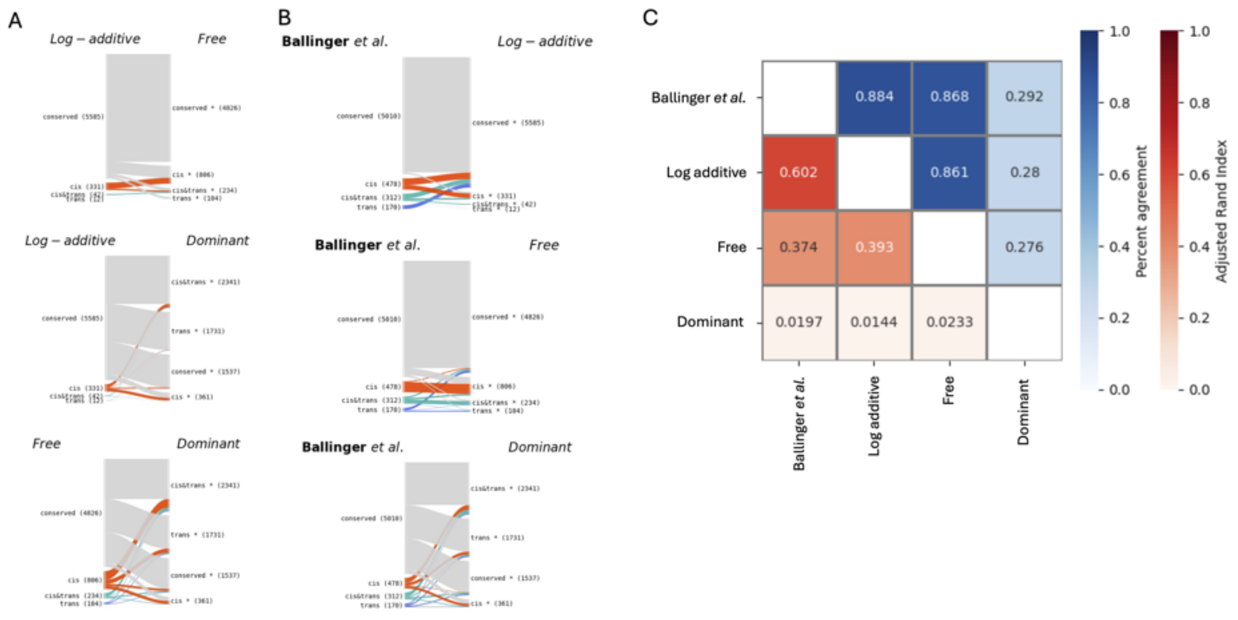

Supp. Fig. 6: Gene regulatory assignments made using three different models (see Methods) fit to BAT tissue samples from male mice in reared in the cold (n = 6 parental strain one, n = 6 two, and n = 6 for hybrid crosses for n = 5,970 genes). Data from Ballinger et al., 2023. A) Alluvial plots showing genes categorized as a particular regulatory group in one generalized linear model versus another. B) Alluvial plots showing genes categorized as a particular regulatory group using the previous published method (Ballinger et al., 2023) versus the generalized linear model approach. C) Percent agreement (number of genes classified the same) between models and previous method (blue) and Adjusted Rand Index between model classification (red).

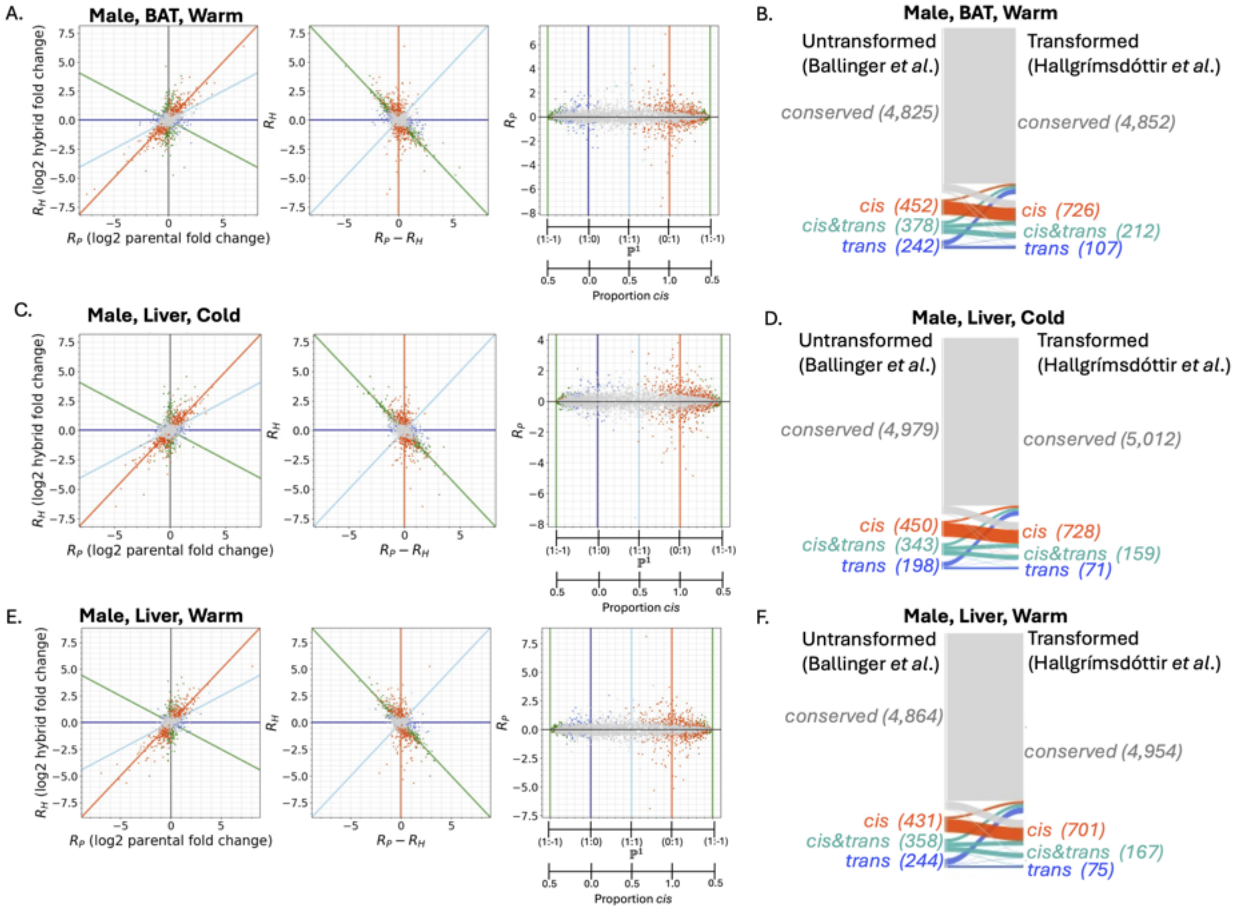

Supp. Fig. 7: A) Predictions using a generalized linear model (free hypothesis, see Methods) fit to brown adipose tissue (BAT) samples taken from 6 male mice each from two wild-derived inbred lines of house mice (one strain from New York, USA, the other from Brazil) and 6 male mice from a cross between the two lines (Ballinger et al. 2023). All mice were raised in a warm environment (21C). Log2 of ratio of parent one to parent two counts (R_P) plotted against the log2 ratio of parent one allele to parent to allele in hybrids (R_H), transformed ratios, and calculated proportion *cis*. The points (N = 5,897) are colored by transformed assigned regulatory category as described in Methods. B) The number in each regulatory assignment from the original study (untransformed), and new assignments (transformed). C) and D) as in A) and B), respectively, but using liver samples taken from mice raised in cold conditions (5C, 5,970 genes). E) and F) as in A), and B) but using liver samples from mice raised in warm conditions (21C, 5,897 genes).

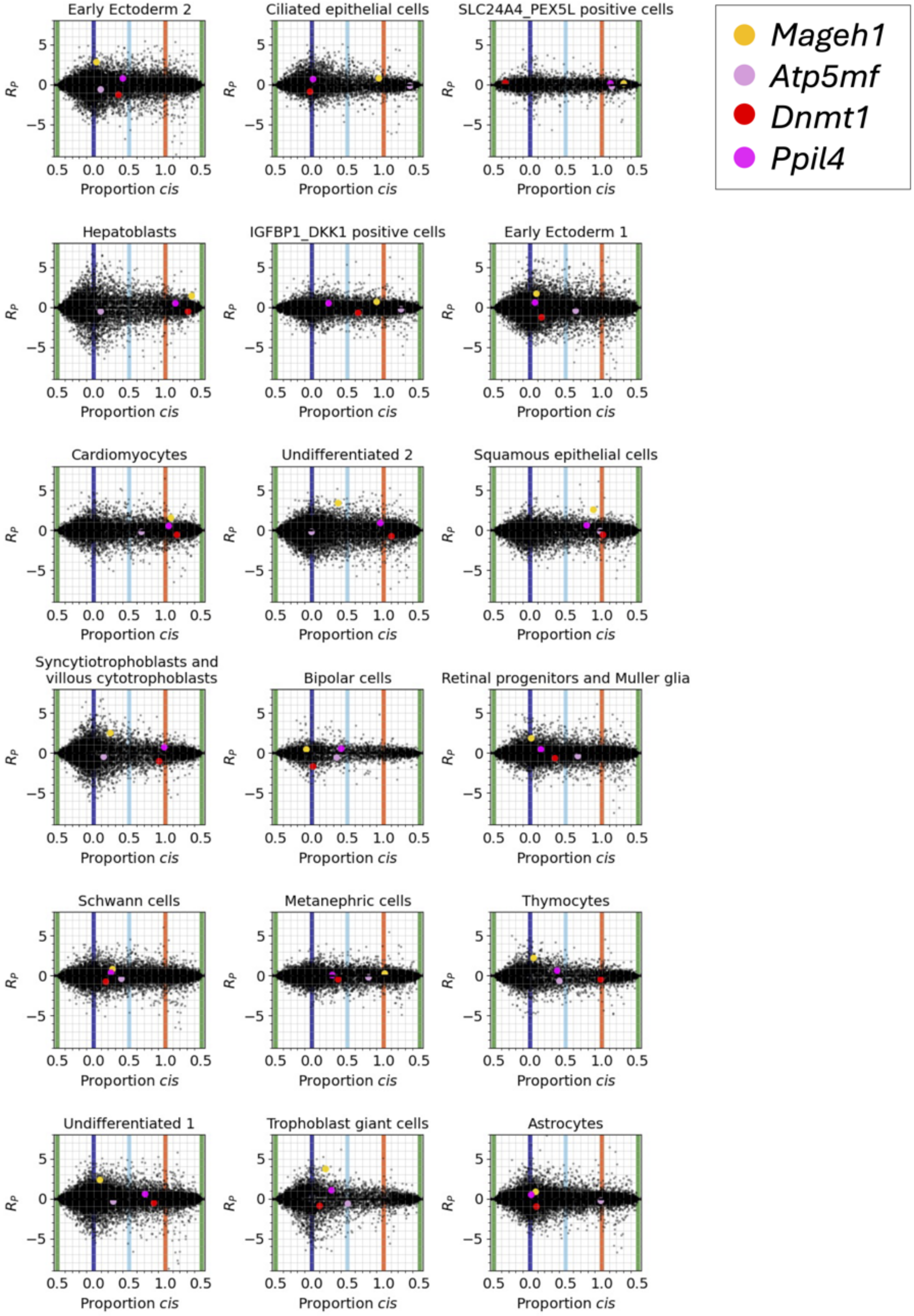

Supp. Fig. 8: Proportion *cis* per gene (n = 14,487) separated by cell type calculated using log2 parent and hybrid ratios from gene expression data from human-chimpanzee cell lines (Barr et al., 2023). Results for 18 of the 72 reported cell types are shown. Genes displayed in Main Fig. 3B are colored as indicated in the legend.

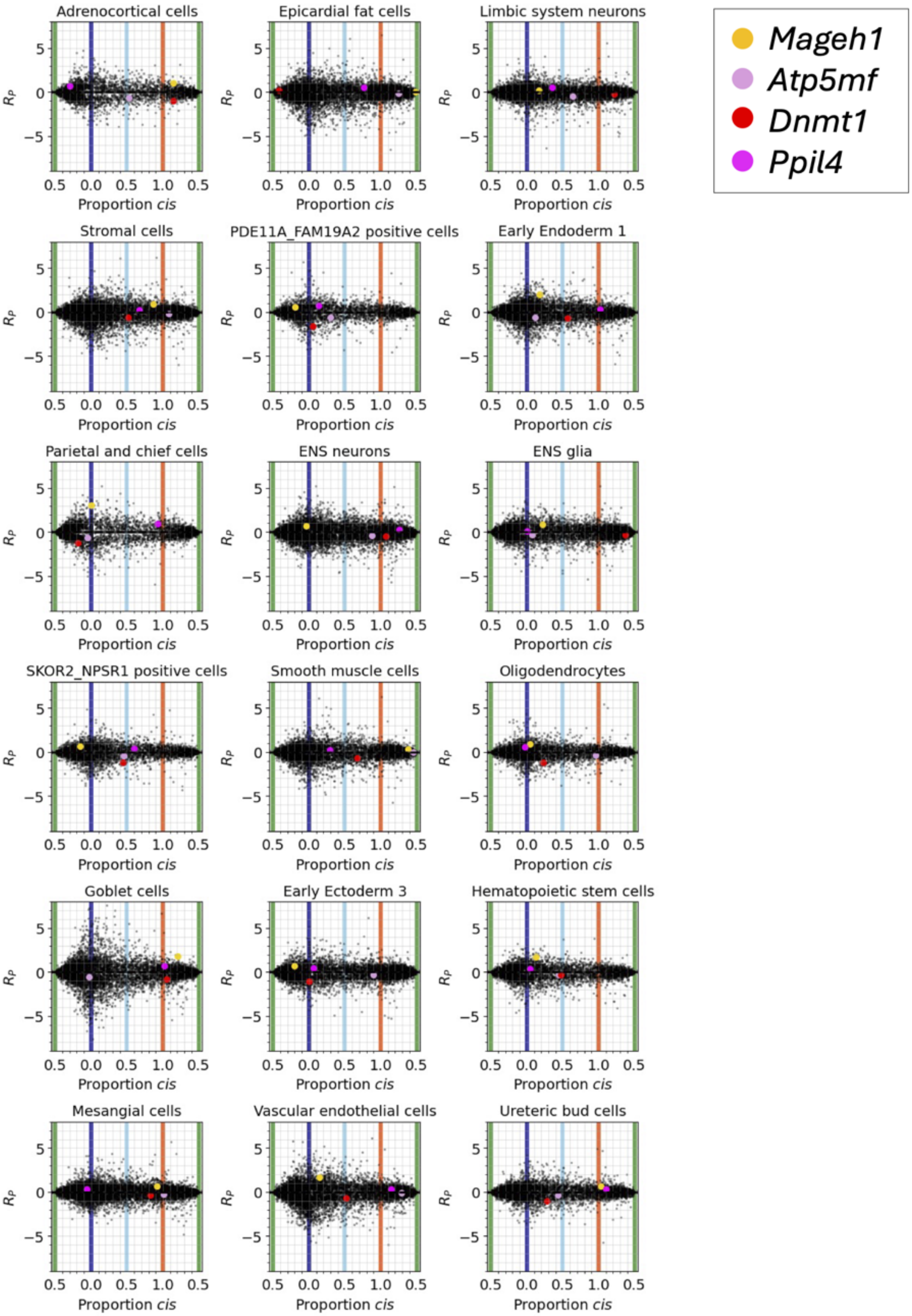

Supp. Fig. 9: Proportion *cis* per gene (n = 14,487) separated by cell type calculated using log2 parent and hybrid ratios from gene expression data from human-chimpanzee cell lines (Barr et al., 2023). Results for 18 of the 72 reported cell types are shown. Genes displayed in Main Fig. 3B are colored as indicated in the legend.

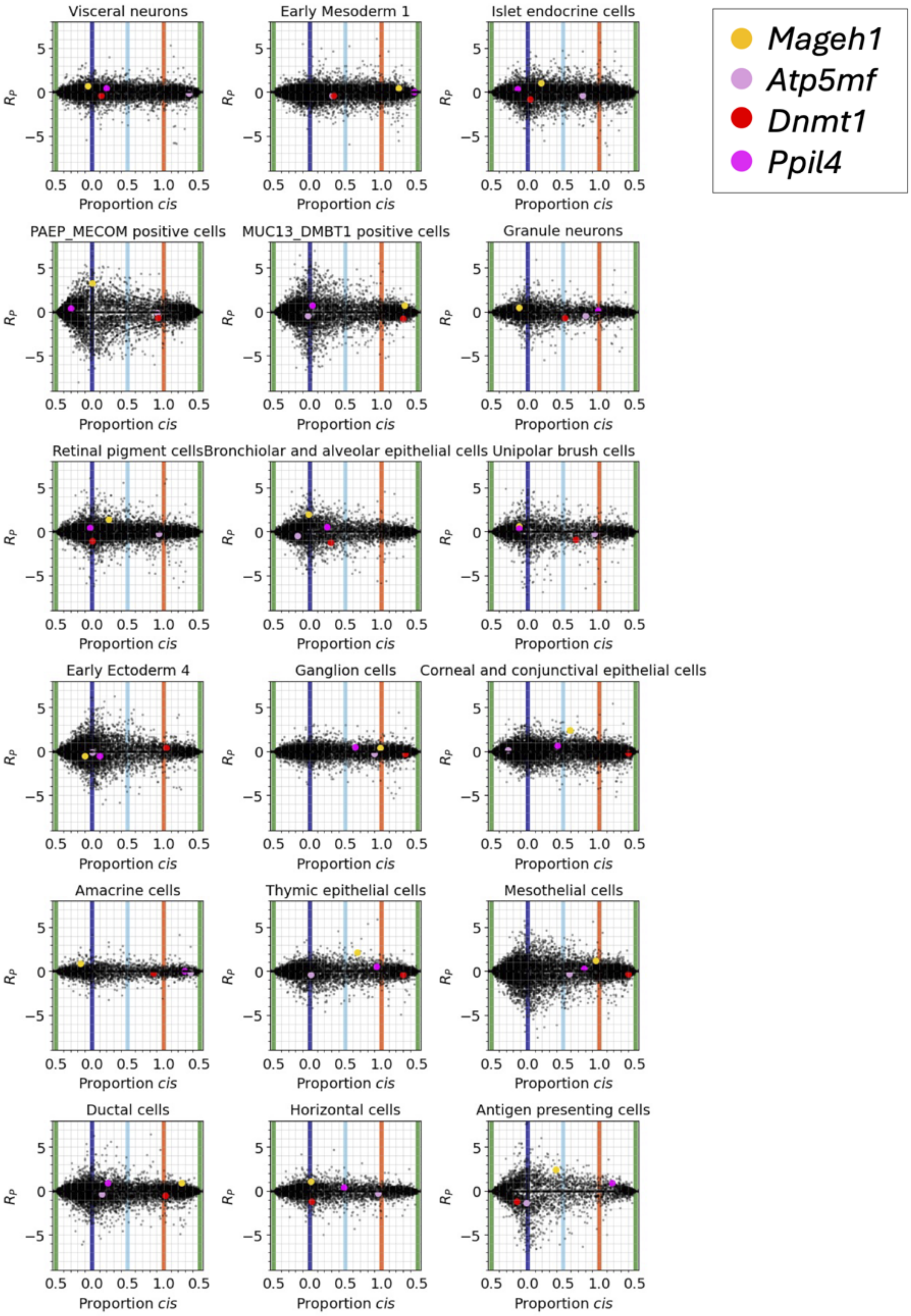

Supp. Fig. 10: Proportion *cis* per gene (n = 14,487) separated by cell type calculated using log2 parent and hybrid ratios from gene expression data from human-chimpanzee cell lines (Barr et al., 2023). Results for 18 of the 72 reported cell types are shown. Genes displayed in Main Fig. 3B are colored as indicated in the legend.

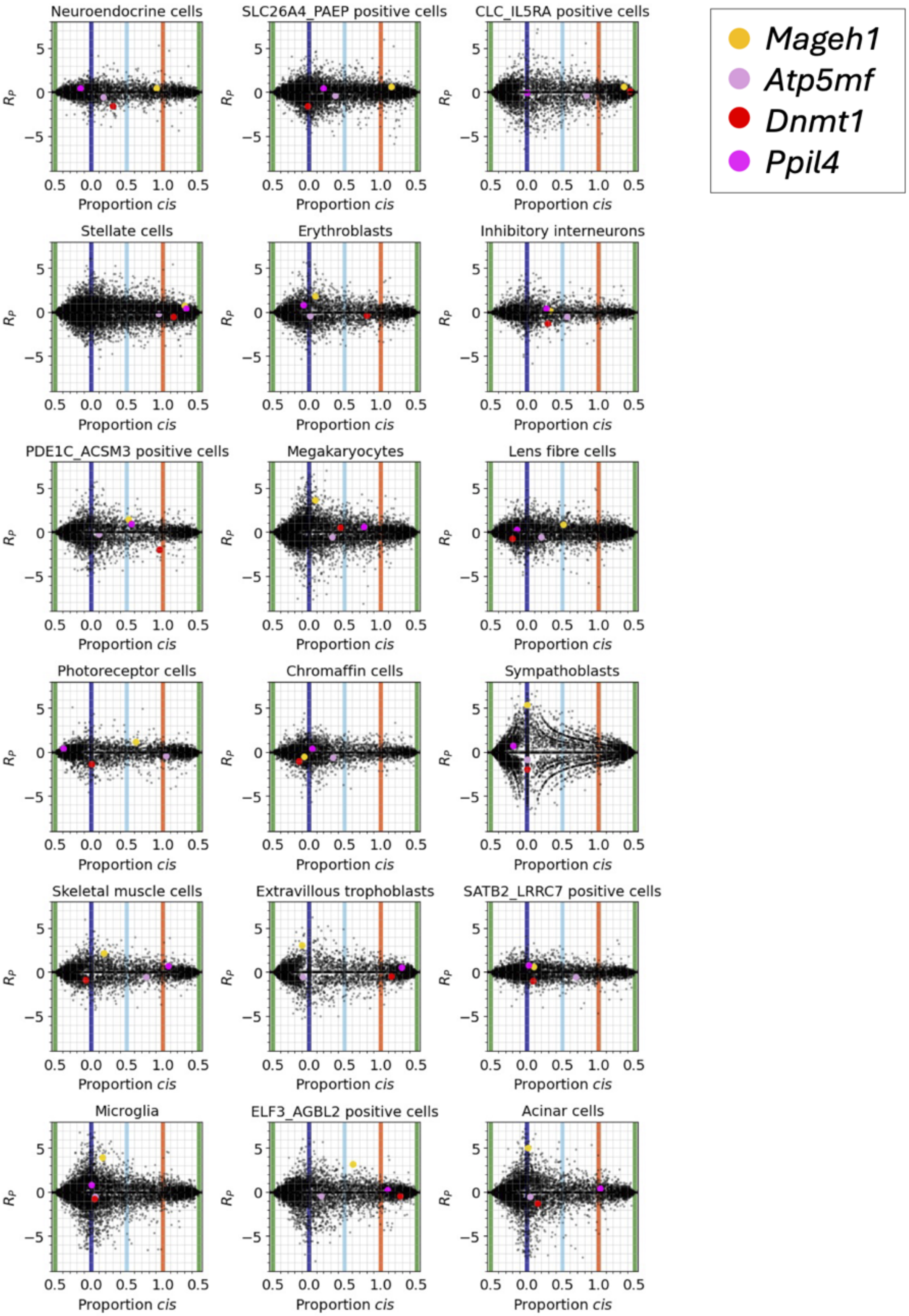

Supp. Fig. 11: Proportion *cis* per gene (n = 14,487) separated by cell type calculated using log2 parent and hybrid ratios from gene expression data from human-chimpanzee cell lines (Barr et al., 2023). Results for 18 of the 72 reported cell types are shown. Genes displayed in Main Fig. 3B are colored as indicated in the legend.

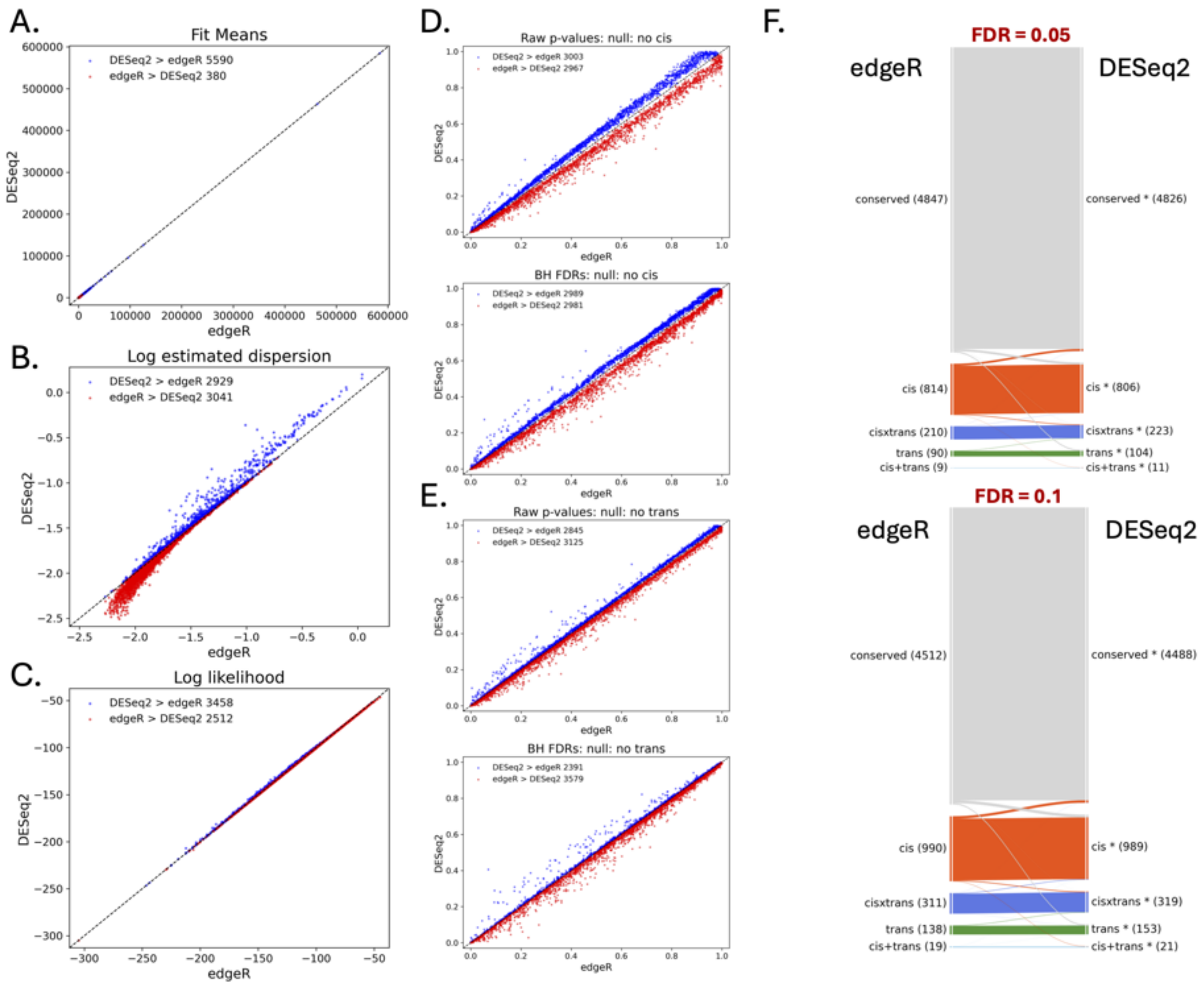

Supp. Fig. 12: A comparison of edgeR (Robinson et al., 2010) and DESeq2 (Love et al., 2014) on brown adipose tissue (BAT) samples taken from mice raised in a cold environment (Ballinger et al., 2023) fit with the free model. In A)-E), values which are larger in DESeq2 are plotted in blue and values larger in edgeR are plotted in red. A) Model fit means for 5,970 genes are nearly identically between edgeR and DESeq2, but B) DESeq2 dispersion estimates per gene are lower for many genes (log of dispersions plotted), which results in C) higher log likelihood values for these genes under DESeq2 fits than edgeR. D) For the null hypothesis of no *cis* regulation (see Methods), log10 of uncorrected p-values (upper) derived from a likelihood ratio test and false discoveries rates (lower) corrected using the Benjamini-Hochberg procedure (Benjamini and Hochberg, 1995) for DESeq2 vs. edgeR. E) As in D), but for the null hypothesis of no *trans* regulation. F) Alluvial plot showing shared and different assigned regulatory groups (see Methods) using edgeR (left) or DESeq2 (right) at different FDR thresholds (0.05, and 0.1).
