# Supplementary material for: Estimating cis and trans contributions to differences in gene regulation
Source: Genetics. 2026 Sep 11;234(3):iyag228. doi: 10.1093/genetics/iyag228 (PMC13732733; doi:10.1093/genetics/iyag228)
Supplement: Supplementary file 2 [file iyag228_supplementary_data.zip › Supplemental_Material_Simulation_Figures_GENETICS-2026-309262.pdf]

# Supplementary simulation figures for: Estimating cis and trans contributions to differences in gene regulation

Ingileif B. Hallgrímsdóttir[1,*], Maria Carilli[1] and Lior Pachter[1,2]

[1]Division of Biology and Biological Engineering, California Institute of Technology, Pasadena, CA 91125, USA
[2]Department of Computing and Mathematical Sciences, California Institute of Technology, Pasadena, CA 91125, USA

[*]Corresponding author: Ingileif Hallgrimsdottir (ingileif@caltech.edu)

## Abstract

This supplement summarizes a genomewide simulation study designed to evaluate cis/trans regulatory inference from parental and hybrid allele-specific count data. Each simulated genomewide data set contained 6000 genes assigned to low-, medium-, or high-expression categories, with simulations varying the number of biological replicates, negative-binomial dispersion, ASE-informative read fraction, and cis/trans effect sizes. We analyzed each simulated data set with XgeneR and with ratio tests based on $R_P$, $R_H$, and $R_P - R_H$ implemented using edgeR, then compared methods using power, type I error, empirical false discovery fraction, ROC/AUC, class agreement, alluvial summaries of regulatory class assignment, parameter recovery, and estimated proportion cis.

**Keywords:** gene regulation; simulation; RNA-seq

## Supplementary simulation figures

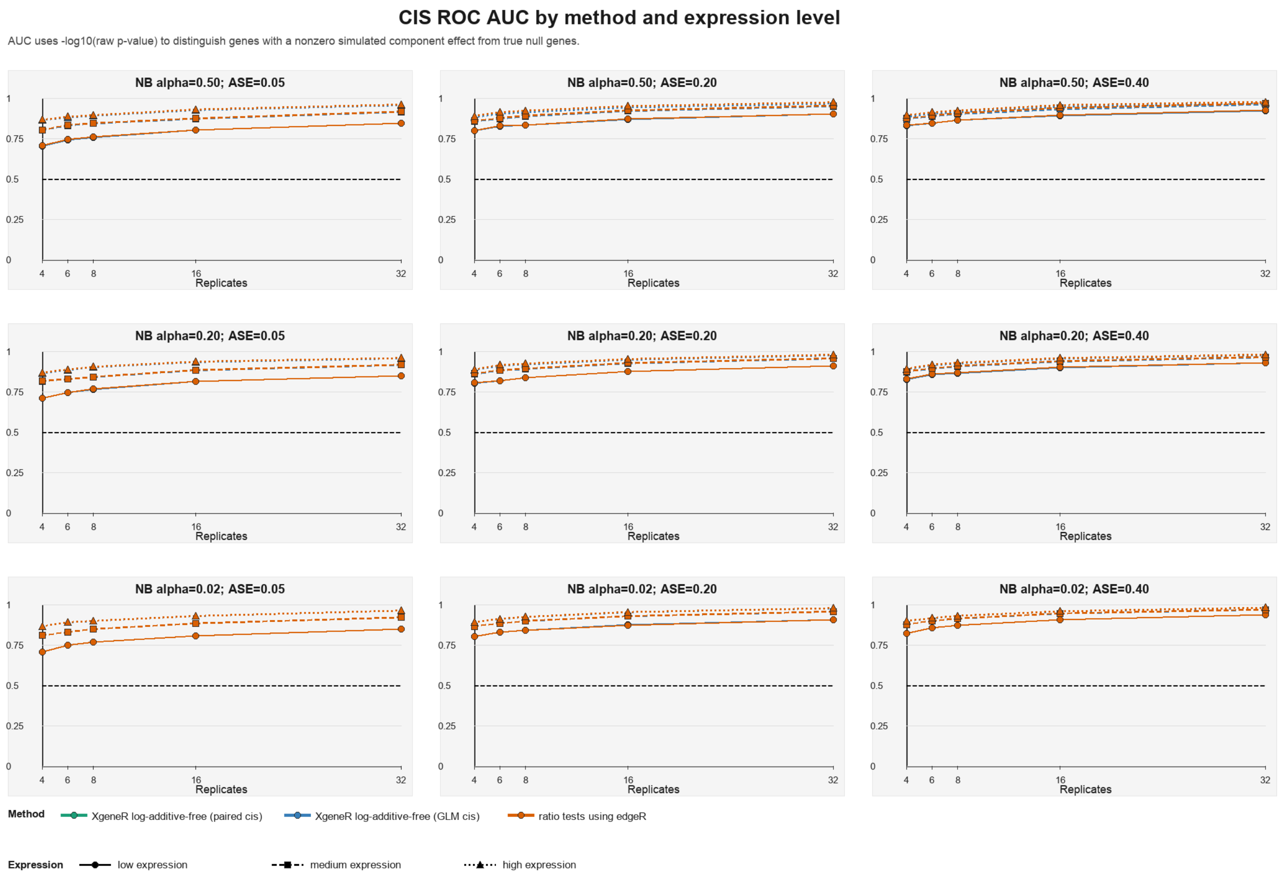


**Figure S1 Cis-test AUC across simulation parameters.** AUC summarizes ranking performance of the cis test across expression levels, dispersion values, ASE-informative fractions, and replicate counts. NB alpha is the negative-binomial dispersion parameter used in count generation, with larger values indicating greater overdispersion; ASE is the simulated allele-specific-expression-informative fraction of hybrid reads. Each genomewide simulation contained 6000 genes assigned to low-, medium-, or high-expression categories; for plots stratified by expression, the reported measure was calculated separately within each expression category. For these summaries, the XgeneR log-additive model and the ratio tests are equivalent, so one curve is plotted directly underneath the other and is not visible.

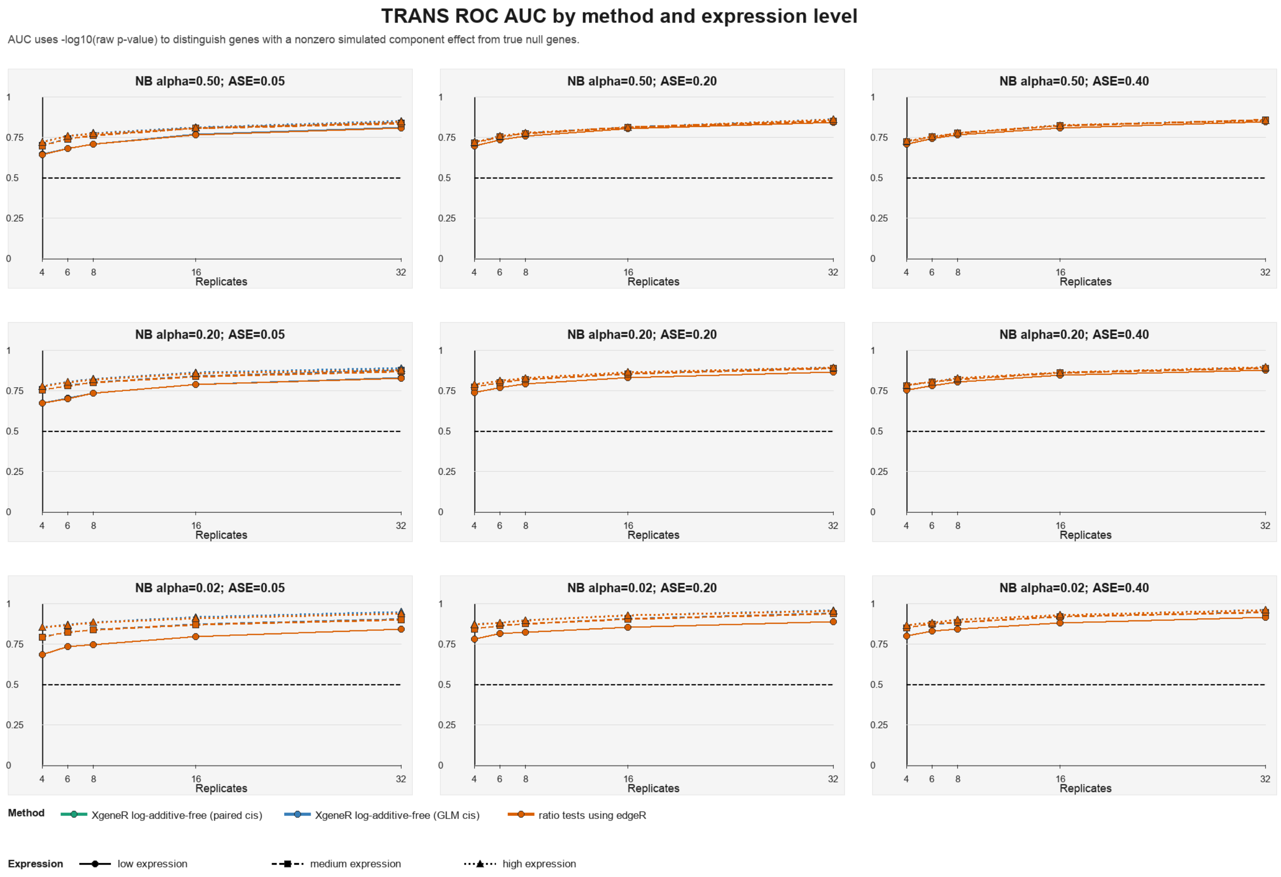


**Figure S2 Trans-test AUC across simulation parameters.** AUC summarizes ranking performance of the trans test across expression levels, dispersion values, ASE-informative fractions, and replicate counts. NB alpha is the negative-binomial dispersion parameter used in count generation, with larger values indicating greater overdispersion; ASE is the simulated allele-specific-expression-informative fraction of hybrid reads. Each genomewide simulation contained 6000 genes assigned to low-, medium-, or high-expression categories; for plots stratified by expression, the reported measure was calculated separately within each expression category. For these summaries, the XgeneR log-additive model and the ratio tests are equivalent, so one curve is plotted directly underneath the other and is not visible.

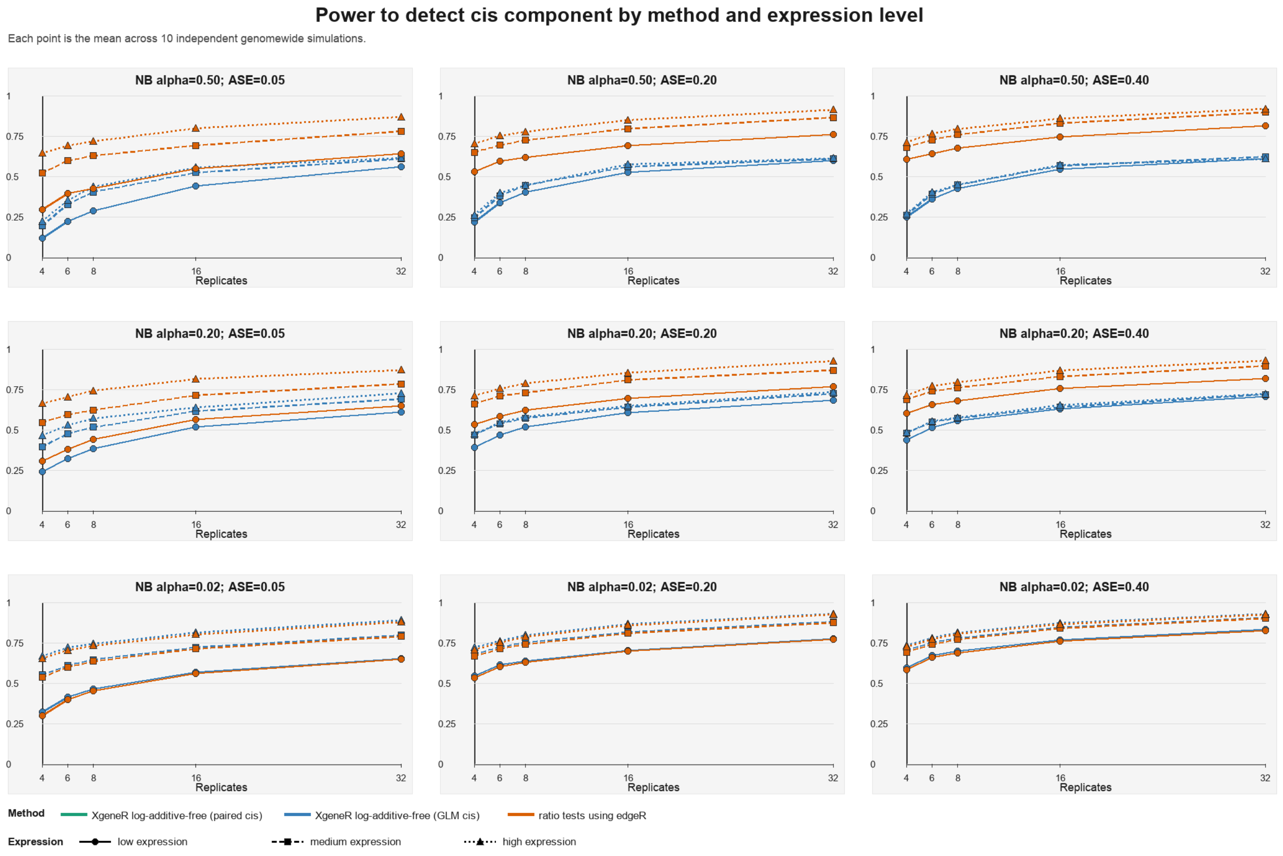


**Figure S3 Power to detect any cis-containing effect.** Cis power is the fraction of genes with a true cis component that were called as any cis-containing class. NB alpha is the negative-binomial dispersion parameter used in count generation, with larger values indicating greater overdispersion; ASE is the simulated allele-specific-expression-informative fraction of hybrid reads. Each genomewide simulation contained 6000 genes assigned to low-, medium-, or high-expression categories; for plots stratified by expression, the reported measure was calculated separately within each expression category. For these summaries, the XgeneR log-additive model and the ratio tests are equivalent, so one curve is plotted directly underneath the other and is not visible.

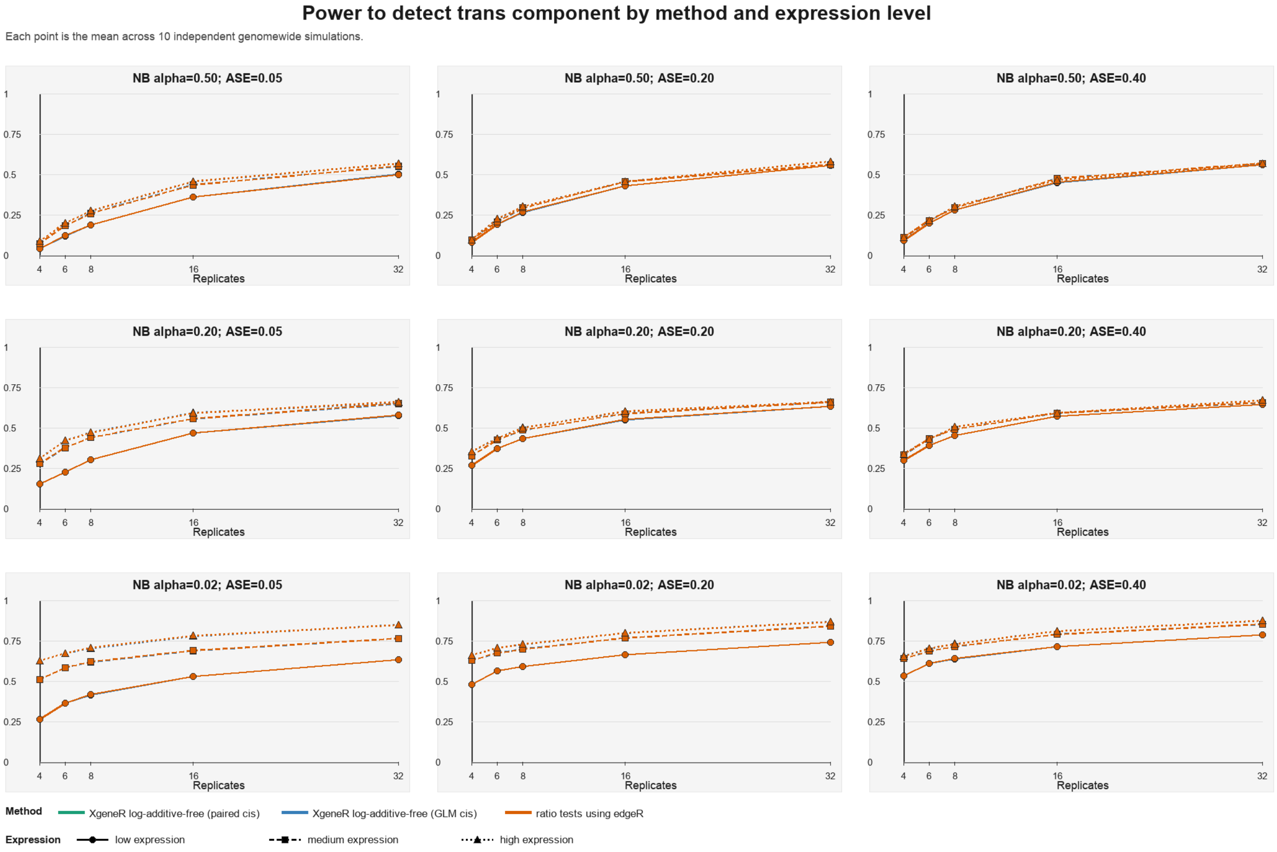


**Figure S4 Power to detect any trans-containing effect.** Trans power is the fraction of genes with a true trans component that were called as any trans-containing class. NB alpha is the negative-binomial dispersion parameter used in count generation, with larger values indicating greater overdispersion; ASE is the simulated allele-specific-expression-informative fraction of hybrid reads. Each genomewide simulation contained 6000 genes assigned to low-, medium-, or high-expression categories; for plots stratified by expression, the reported measure was calculated separately within each expression category. For these summaries, the XgeneR log-additive model and the ratio tests are equivalent, so one curve is plotted directly underneath the other and is not visible.

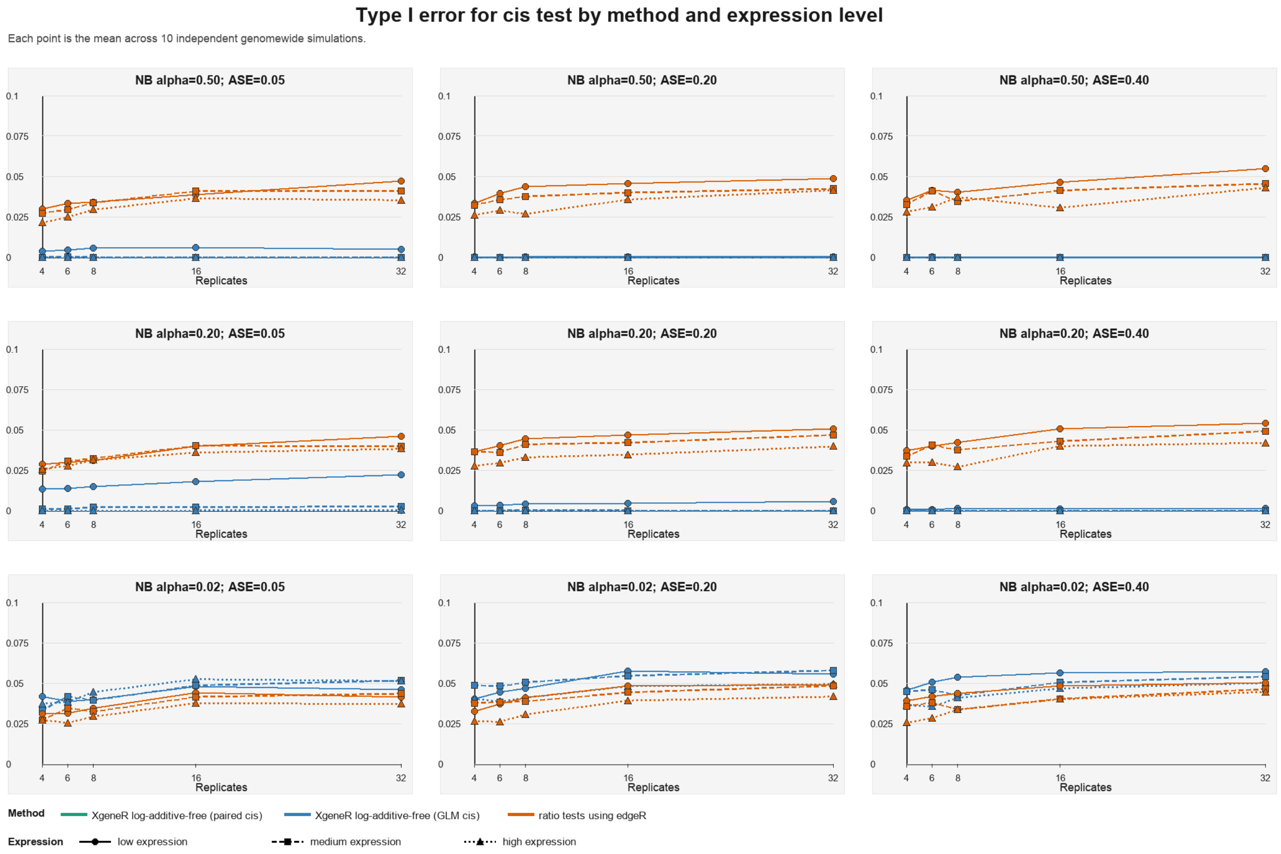


**Figure S5 Cis false positive rate among true cis-null genes.** Type I error is calculated from the FDR-thresholded calls using the known simulation truth. NB alpha is the negative-binomial dispersion parameter used in count generation, with larger values indicating greater overdispersion; ASE is the simulated allele-specific-expression-informative fraction of hybrid reads. Each genomewide simulation contained 6000 genes assigned to low-, medium-, or high-expression categories; for plots stratified by expression, the reported measure was calculated separately within each expression category. For these summaries, the XgeneR log-additive model and the ratio tests are equivalent, so one curve is plotted directly underneath the other and is not visible.

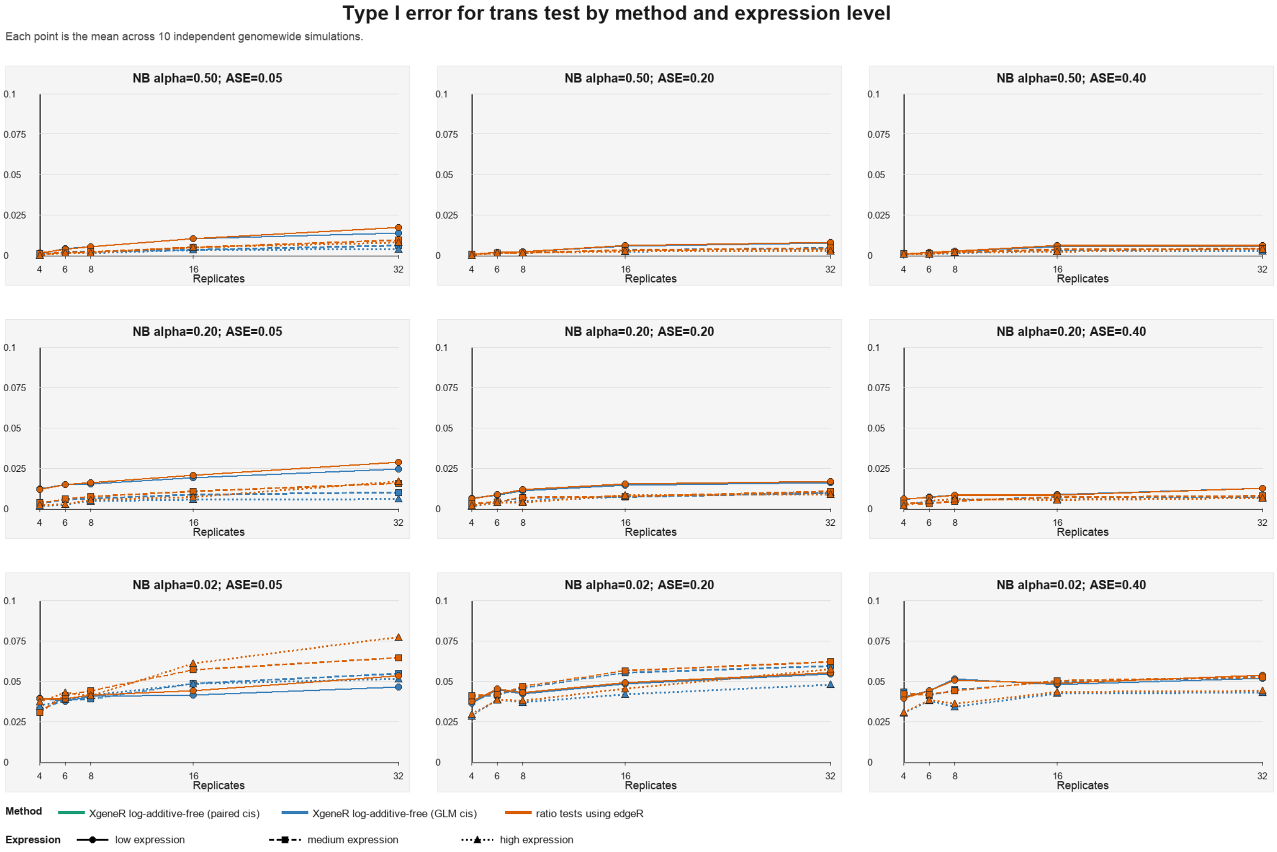


**Figure S6 Trans false positive rate among true trans-null genes.** Type I error is calculated from the FDR-thresholded calls using the known simulation truth. NB alpha is the negative-binomial dispersion parameter used in count generation, with larger values indicating greater overdispersion; ASE is the simulated allele-specific-expression-informative fraction of hybrid reads. Each genomewide simulation contained 6000 genes assigned to low-, medium-, or high-expression categories; for plots stratified by expression, the reported measure was calculated separately within each expression category. For these summaries, the XgeneR log-additive model and the ratio tests are equivalent, so one curve is plotted directly underneath the other and is not visible.

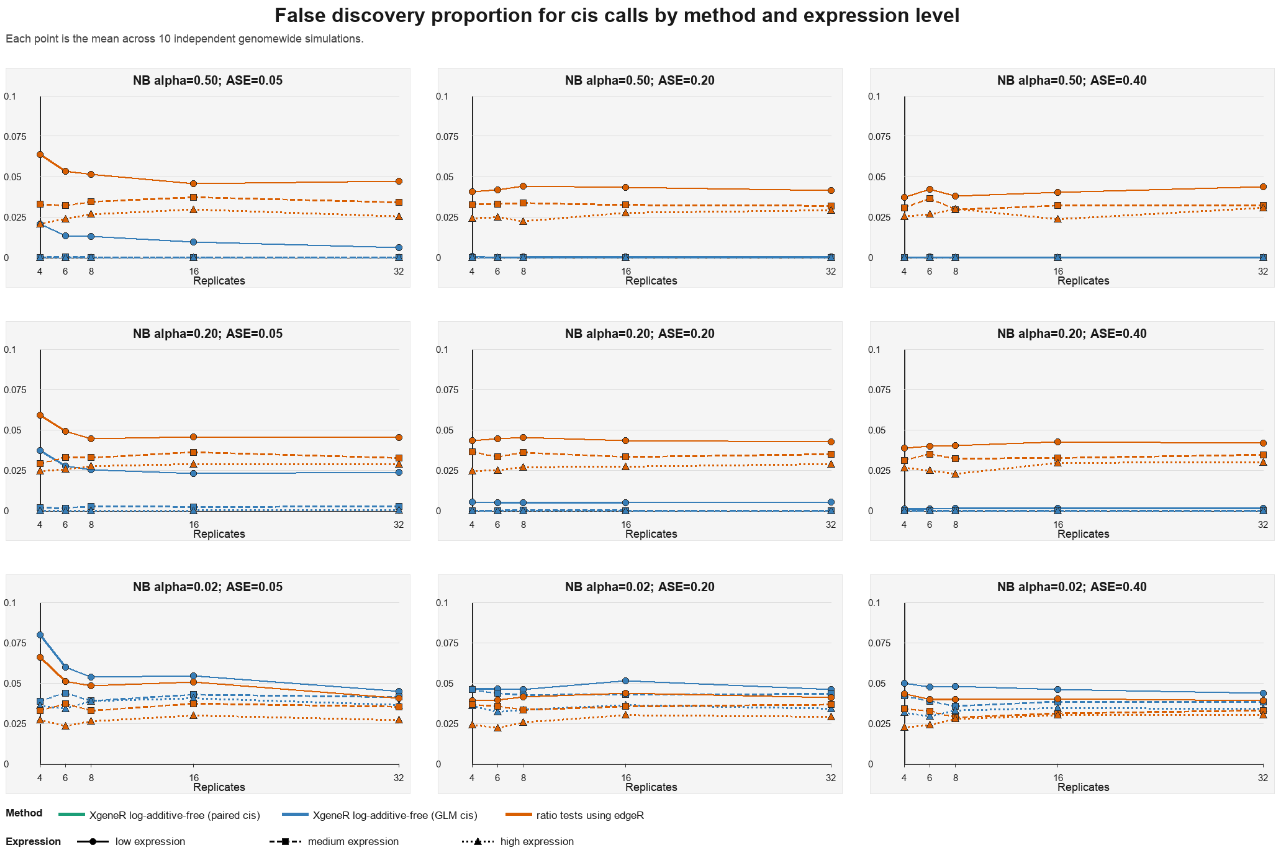


**Figure S7 Empirical false discovery fraction among cis calls.** The empirical FDP is the fraction of cis-containing calls that do not have a true cis component. NB alpha is the negative-binomial dispersion parameter used in count generation, with larger values indicating greater overdispersion; ASE is the simulated allele-specific-expression-informative fraction of hybrid reads. Each genomewide simulation contained 6000 genes assigned to low-, medium-, or high-expression categories; for plots stratified by expression, the reported measure was calculated separately within each expression category. For these summaries, the XgeneR log-additive model and the ratio tests are equivalent, so one curve is plotted directly underneath the other and is not visible.

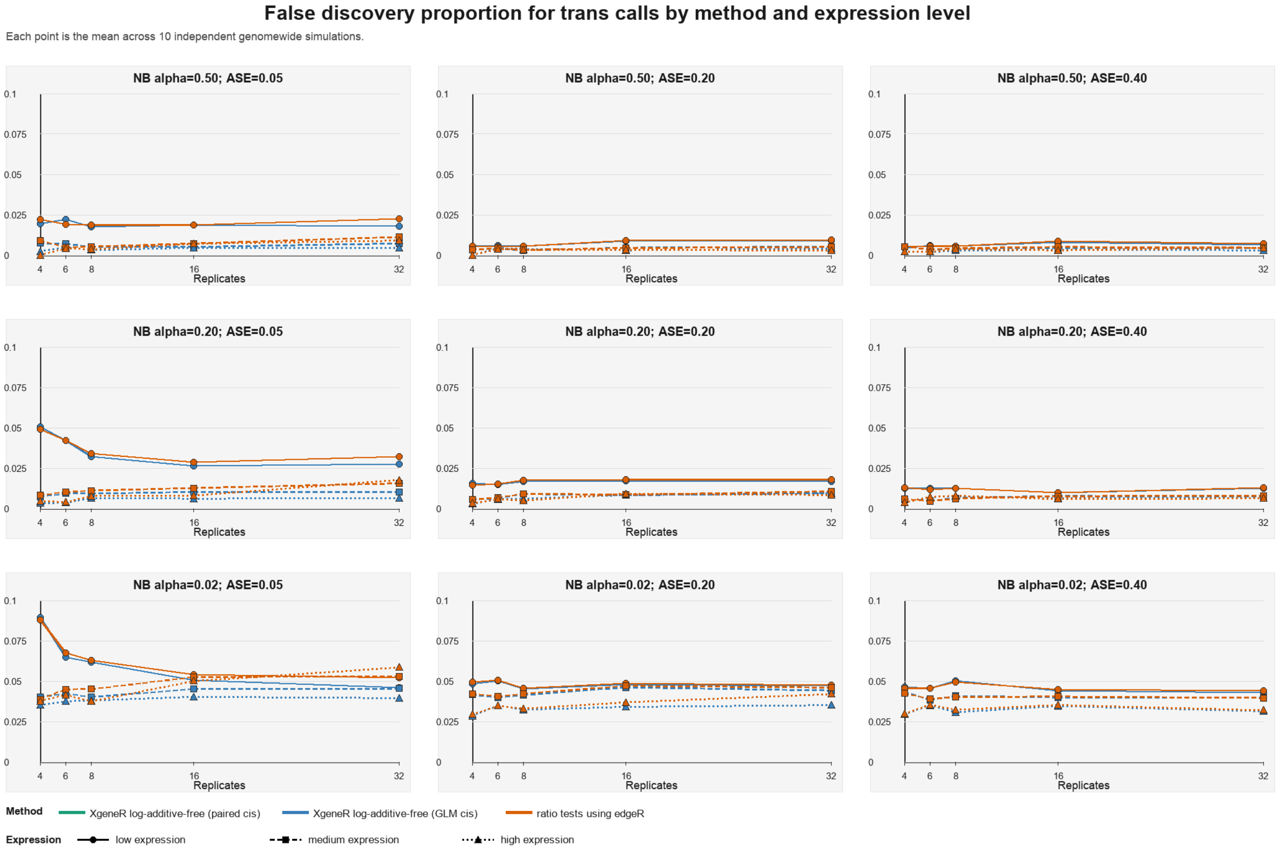


**Figure S8 Empirical false discovery fraction among trans calls.** The empirical FDP is the fraction of trans-containing calls that do not have a true trans component. NB alpha is the negative-binomial dispersion parameter used in count generation, with larger values indicating greater overdispersion; ASE is the simulated allele-specific-expression-informative fraction of hybrid reads. Each genomewide simulation contained 6000 genes assigned to low-, medium-, or high-expression categories; for plots stratified by expression, the reported measure was calculated separately within each expression category. For these summaries, the XgeneR log-additive model and the ratio tests are equivalent, so one curve is plotted directly underneath the other and is not visible.

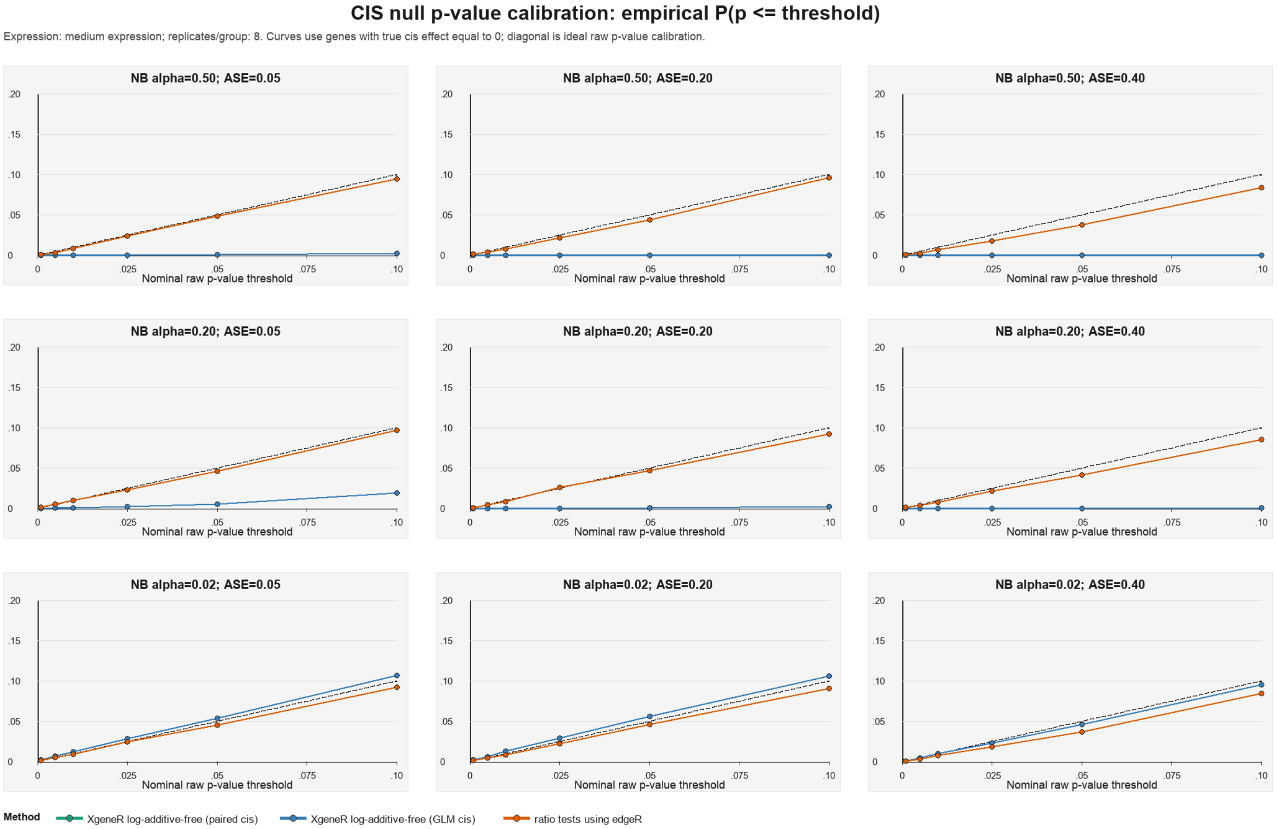


**Figure S9 Null p-value calibration for cis tests in medium-expression genes with 8 replicates.** The curves summarize the distribution of raw p-values among genes with no true cis effect. NB alpha is the negative-binomial dispersion parameter used in count generation, with larger values indicating greater overdispersion; ASE is the simulated allele-specific-expression-informative fraction of hybrid reads. Each genomewide simulation contained 6000 genes assigned to low-, medium-, or high-expression categories; for plots stratified by expression, the reported measure was calculated separately within each expression category. For these summaries, the XgeneR log-additive model and the ratio tests are equivalent, so one curve is plotted directly underneath the other and is not visible.

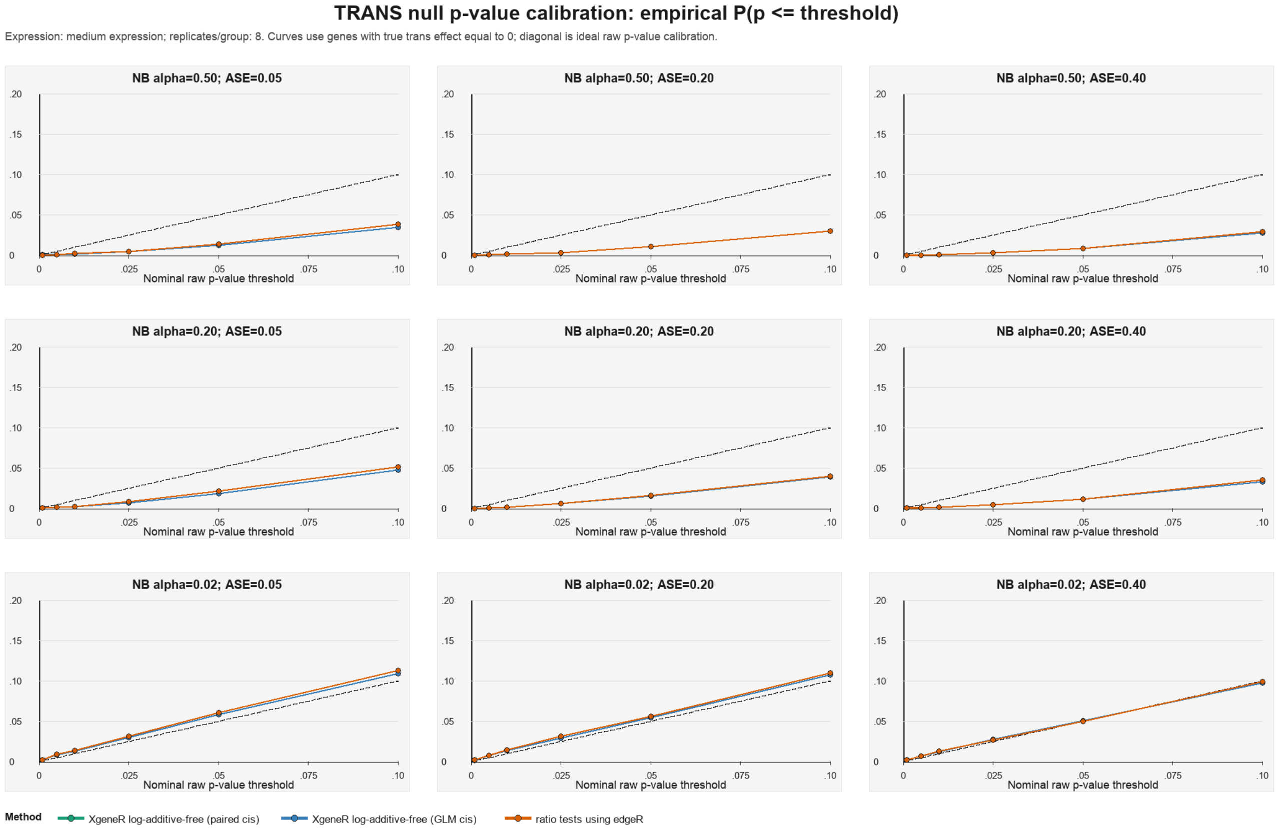


**Figure S10 Null p-value calibration for trans tests in medium-expression genes with 8 replicates.** The curves summarize the distribution of raw p-values among genes with no true trans effect. NB alpha is the negative-binomial dispersion parameter used in count generation, with larger values indicating greater overdispersion; ASE is the simulated allele-specific-expression-informative fraction of hybrid reads. Each genomewide simulation contained 6000 genes assigned to low-, medium-, or high-expression categories; for plots stratified by expression, the reported measure was calculated separately within each expression category. For these summaries, the XgeneR log-additive model and the ratio tests are equivalent, so one curve is plotted directly underneath the other and is not visible.

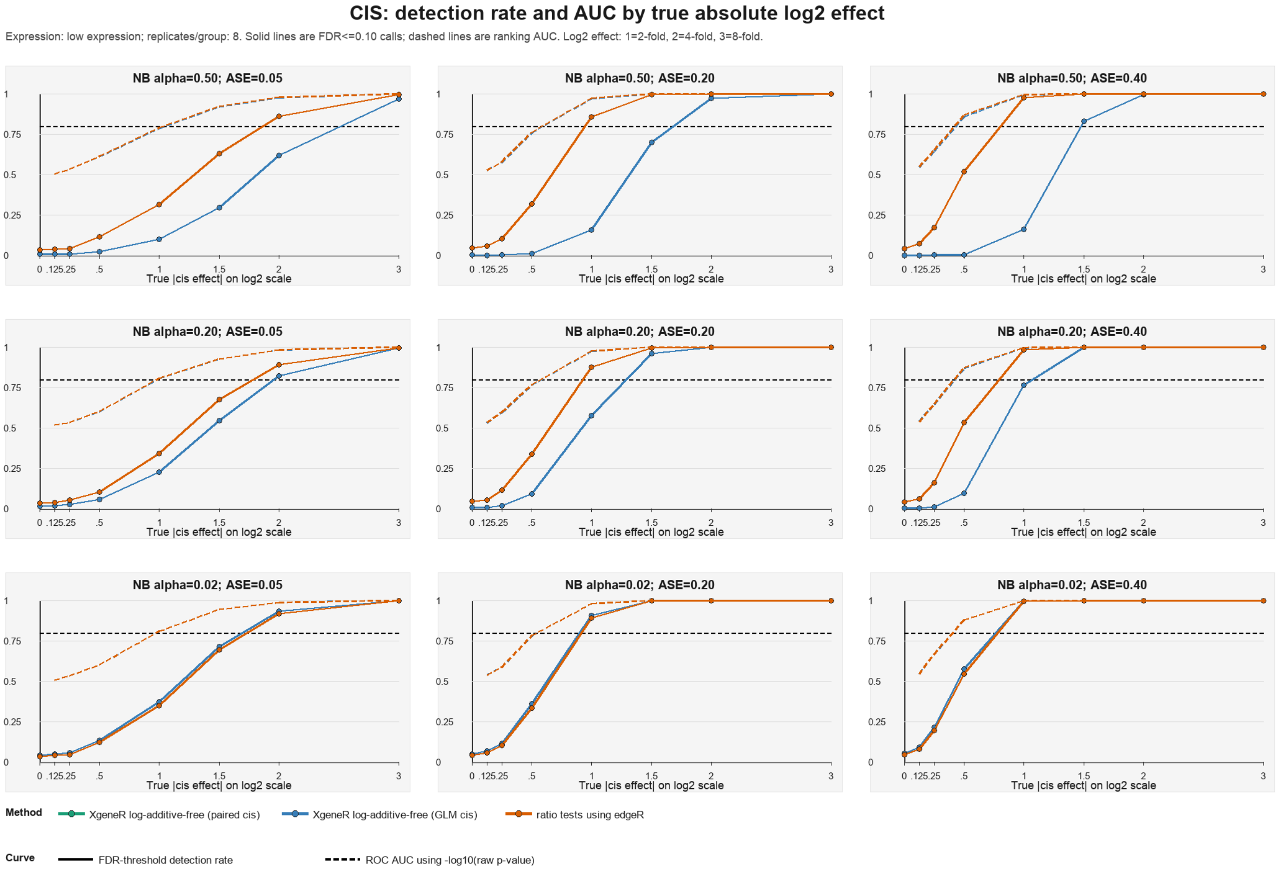


**Figure S11 Cis detection and AUC by true effect size for low-expression genes with 8 replicates.** Panels stratify NB dispersion and ASE-informative fraction. NB alpha is the negative-binomial dispersion parameter used in count generation, with larger values indicating greater overdispersion; ASE is the simulated allele-specific-expression-informative fraction of hybrid reads. Each genomewide simulation contained 6000 genes assigned to low-, medium-, or high-expression categories; for plots stratified by expression, the reported measure was calculated separately within each expression category. For these summaries, the XgeneR log-additive model and the ratio tests are equivalent, so one curve is plotted directly underneath the other and is not visible.

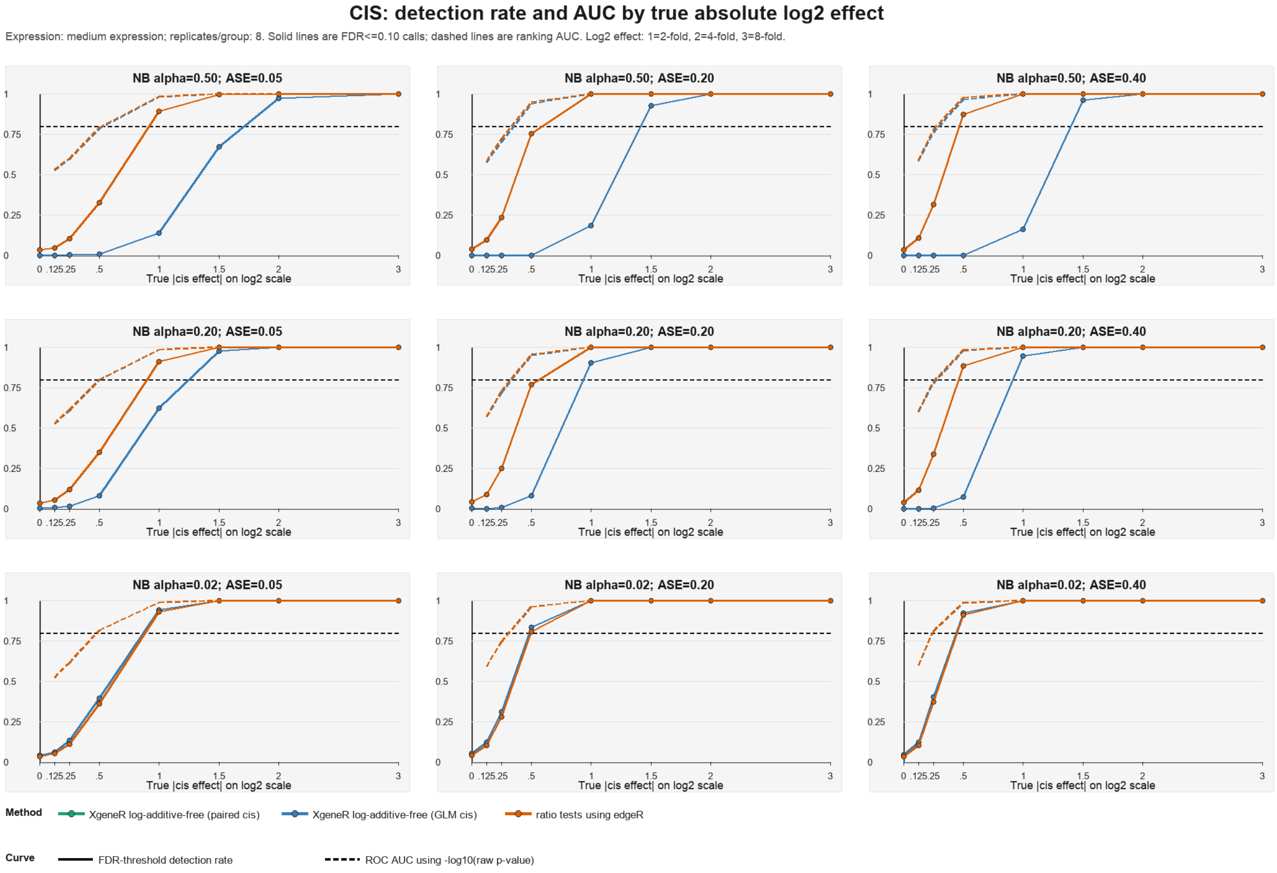


**Figure S12 Cis detection and AUC by true effect size for medium-expression genes with 8 replicates.** Panels stratify NB dispersion and ASE-informative fraction. NB alpha is the negative-binomial dispersion parameter used in count generation, with larger values indicating greater overdispersion; ASE is the simulated allele-specific-expression-informative fraction of hybrid reads. Each genomewide simulation contained 6000 genes assigned to low-, medium-, or high-expression categories; for plots stratified by expression, the reported measure was calculated separately within each expression category. For these summaries, the XgeneR log-additive model and the ratio tests are equivalent, so one curve is plotted directly underneath the other and is not visible.

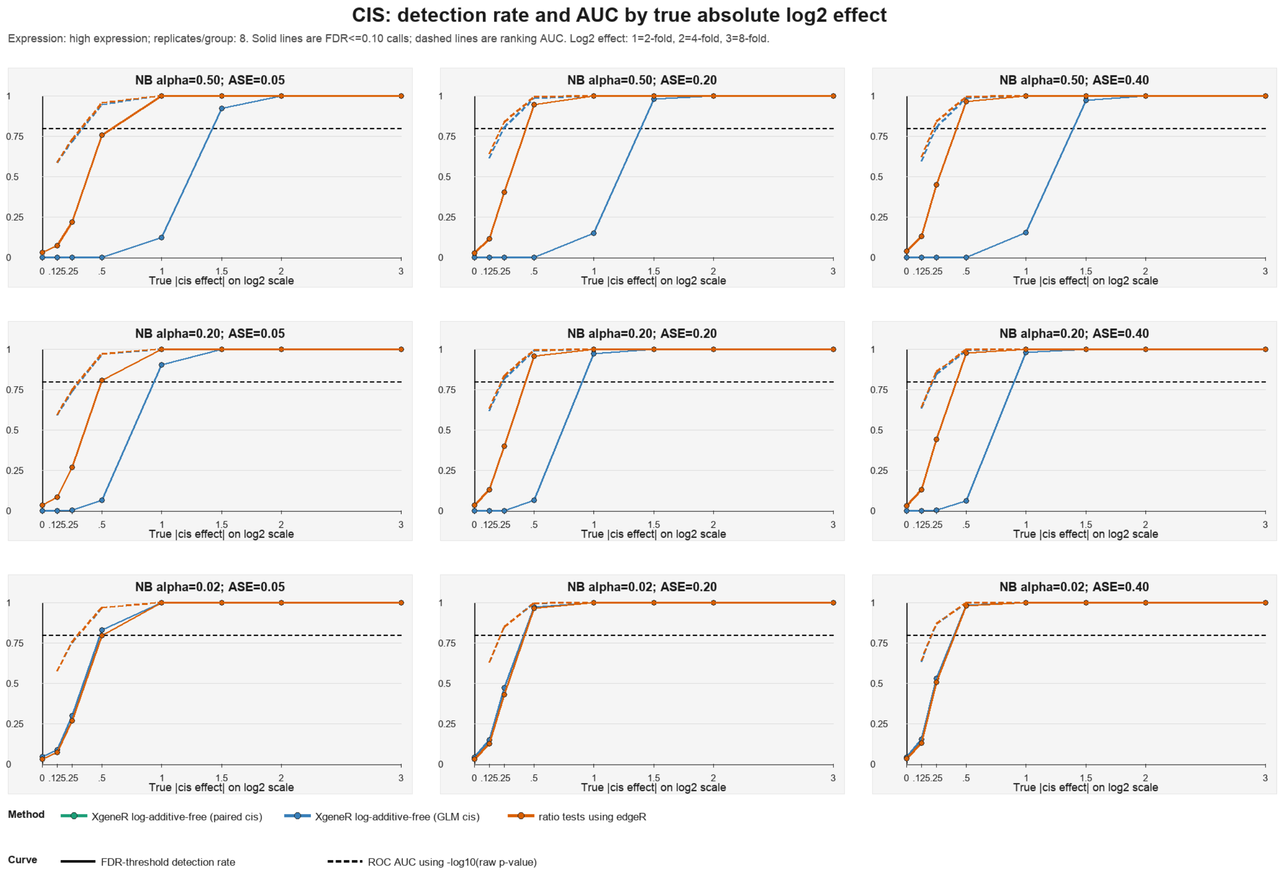


**Figure S13 Cis detection and AUC by true effect size for high-expression genes with 8 replicates.** Panels stratify NB dispersion and ASE-informative fraction. NB alpha is the negative-binomial dispersion parameter used in count generation, with larger values indicating greater overdispersion; ASE is the simulated allele-specific-expression-informative fraction of hybrid reads. Each genomewide simulation contained 6000 genes assigned to low-, medium-, or high-expression categories; for plots stratified by expression, the reported measure was calculated separately within each expression category. For these summaries, the XgeneR log-additive model and the ratio tests are equivalent, so one curve is plotted directly underneath the other and is not visible.

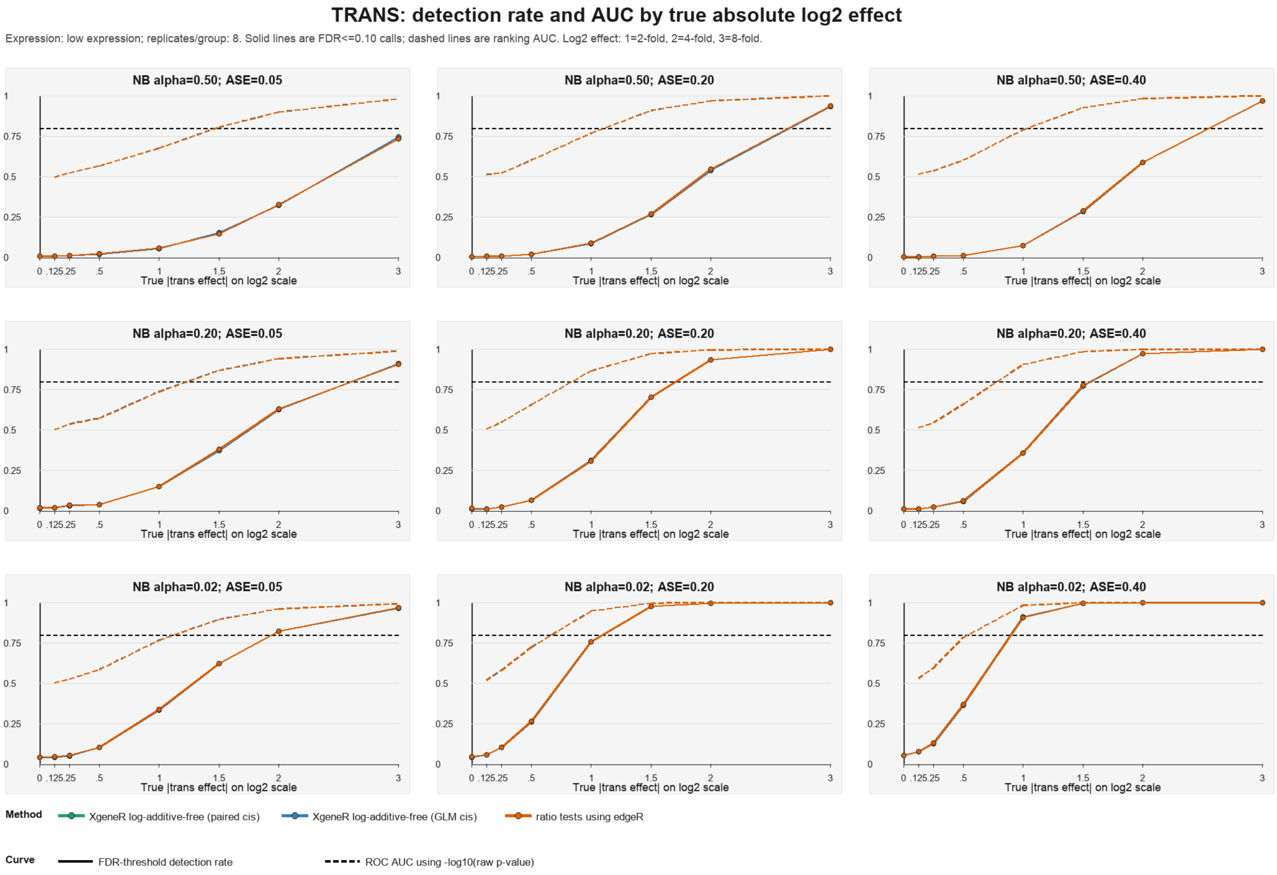


**Figure S14 Trans detection and AUC by true effect size for low-expression genes with 8 replicates.** Panels stratify NB dispersion and ASE-informative fraction. NB alpha is the negative-binomial dispersion parameter used in count generation, with larger values indicating greater overdispersion; ASE is the simulated allele-specific-expression-informative fraction of hybrid reads. Each genomewide simulation contained 6000 genes assigned to low-, medium-, or high-expression categories; for plots stratified by expression, the reported measure was calculated separately within each expression category. For these summaries, the XgeneR log-additive model and the ratio tests are equivalent, so one curve is plotted directly underneath the other and is not visible.

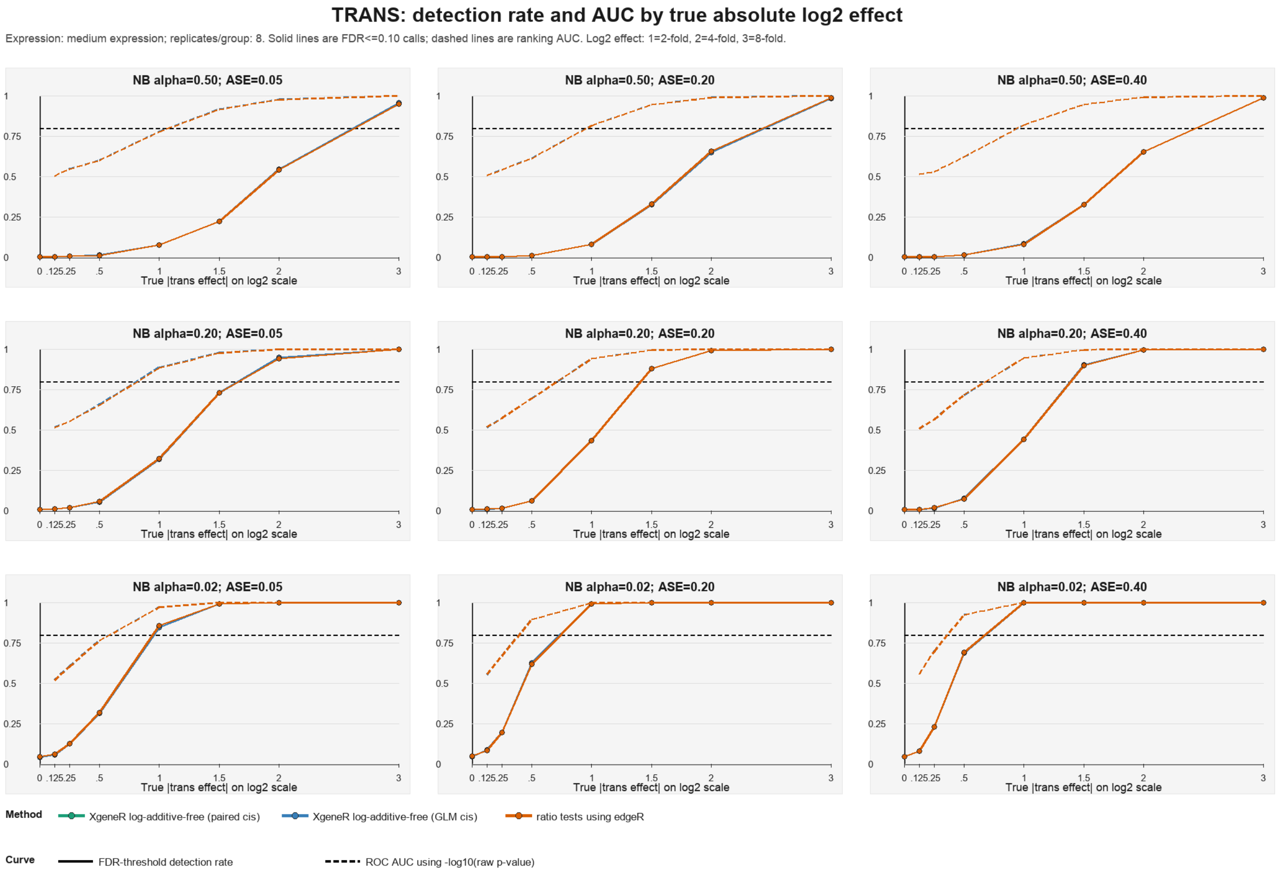


**Figure S15 Trans detection and AUC by true effect size for medium-expression genes with 8 replicates.** Panels stratify NB dispersion and ASE-informative fraction. NB alpha is the negative-binomial dispersion parameter used in count generation, with larger values indicating greater overdispersion; ASE is the simulated allele-specific-expression-informative fraction of hybrid reads. Each genomewide simulation contained 6000 genes assigned to low-, medium-, or high-expression categories; for plots stratified by expression, the reported measure was calculated separately within each expression category. For these summaries, the XgeneR log-additive model and the ratio tests are equivalent, so one curve is plotted directly underneath the other and is not visible.

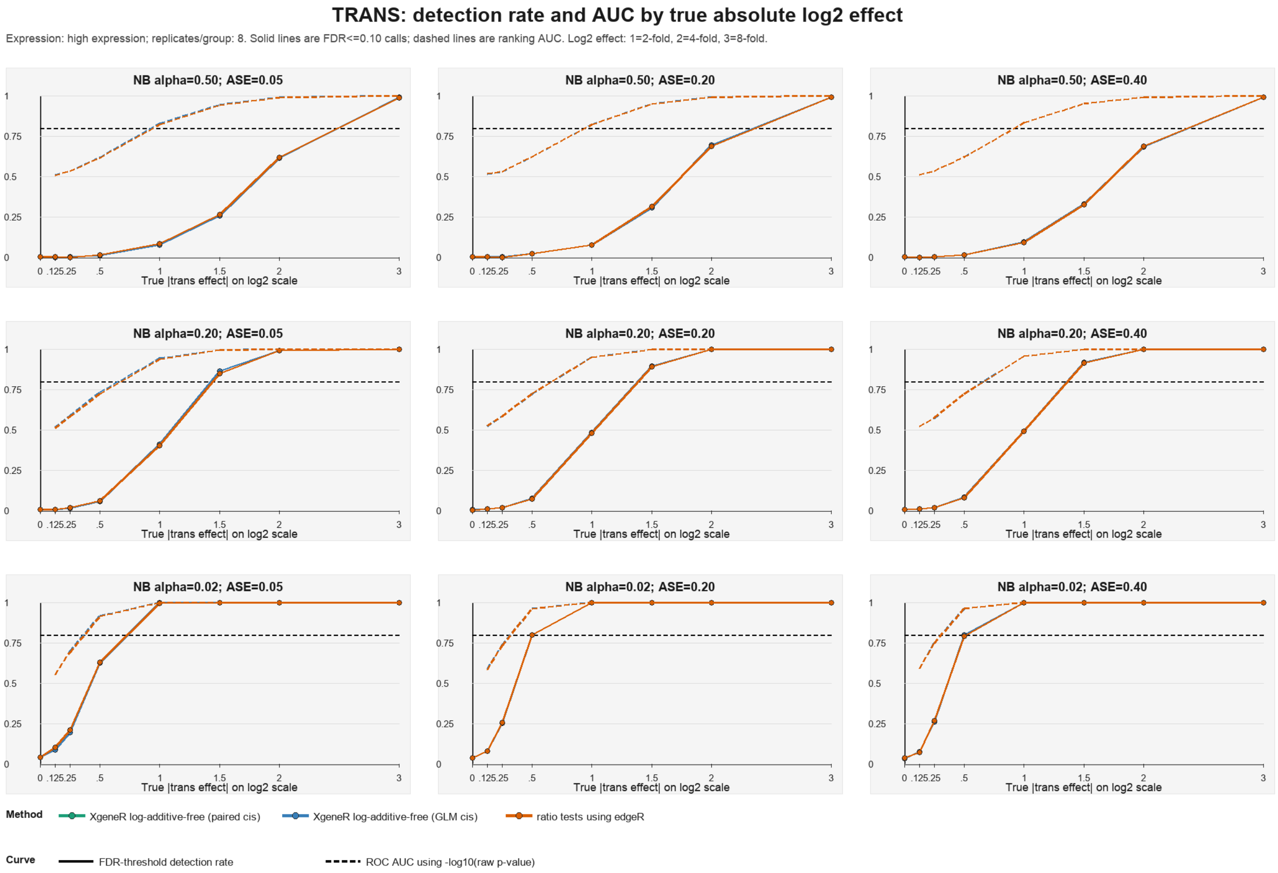


**Figure S16 Trans detection and AUC by true effect size for high-expression genes with 8 replicates.** Panels stratify NB dispersion and ASE-informative fraction. NB alpha is the negative-binomial dispersion parameter used in count generation, with larger values indicating greater overdispersion; ASE is the simulated allele-specific-expression-informative fraction of hybrid reads. Each genomewide simulation contained 6000 genes assigned to low-, medium-, or high-expression categories; for plots stratified by expression, the reported measure was calculated separately within each expression category. For these summaries, the XgeneR log-additive model and the ratio tests are equivalent, so one curve is plotted directly underneath the other and is not visible.

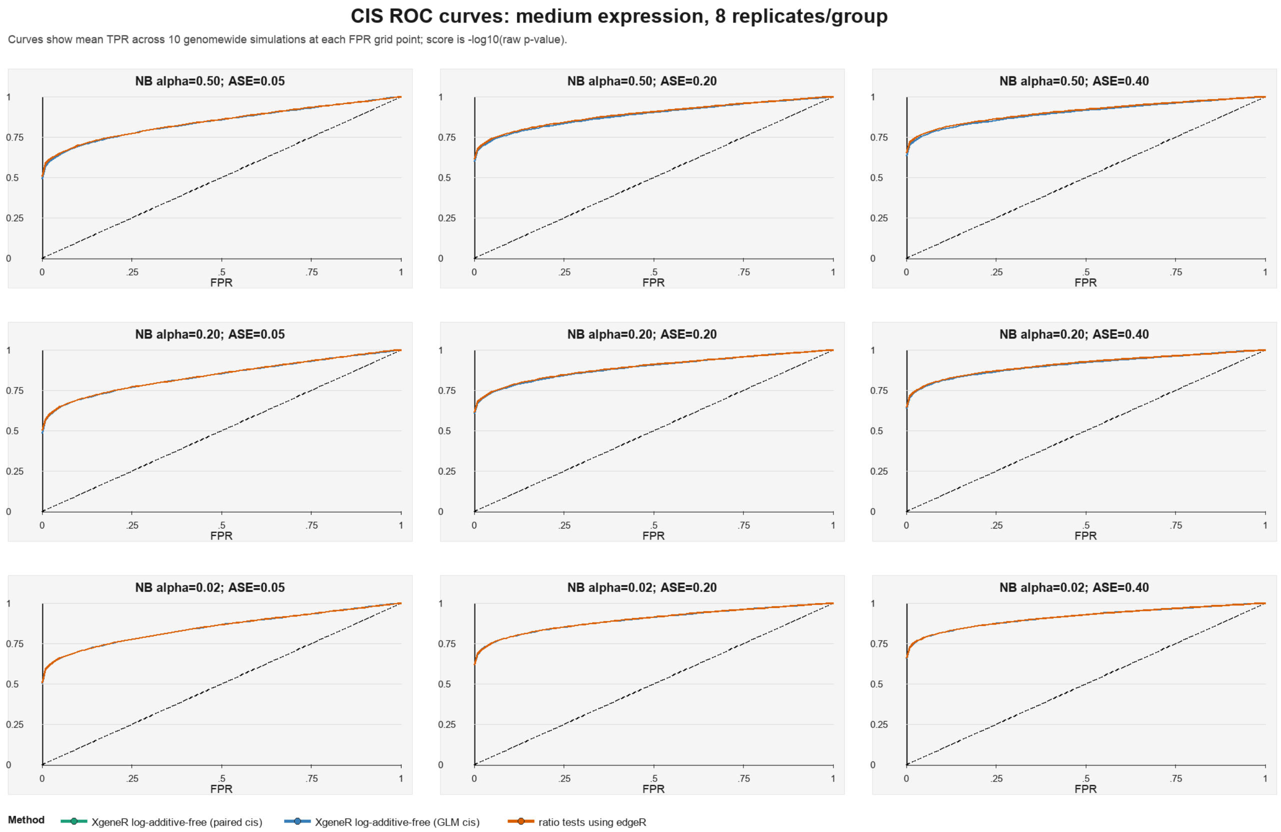


**Figure S17 ROC curves for cis detection in medium-expression genes with 8 replicates.** Curves compare XgeneR with ratio tests using edgeR across NB dispersion and ASE-informative fraction panels. NB alpha is the negative-binomial dispersion parameter used in count generation, with larger values indicating greater overdispersion; ASE is the simulated allele-specific-expression-informative fraction of hybrid reads. Each genomewide simulation contained 6000 genes assigned to low-, medium-, or high-expression categories; for plots stratified by expression, the reported measure was calculated separately within each expression category. For these summaries, the XgeneR log-additive model and the ratio tests are equivalent, so one curve is plotted directly underneath the other and is not visible.

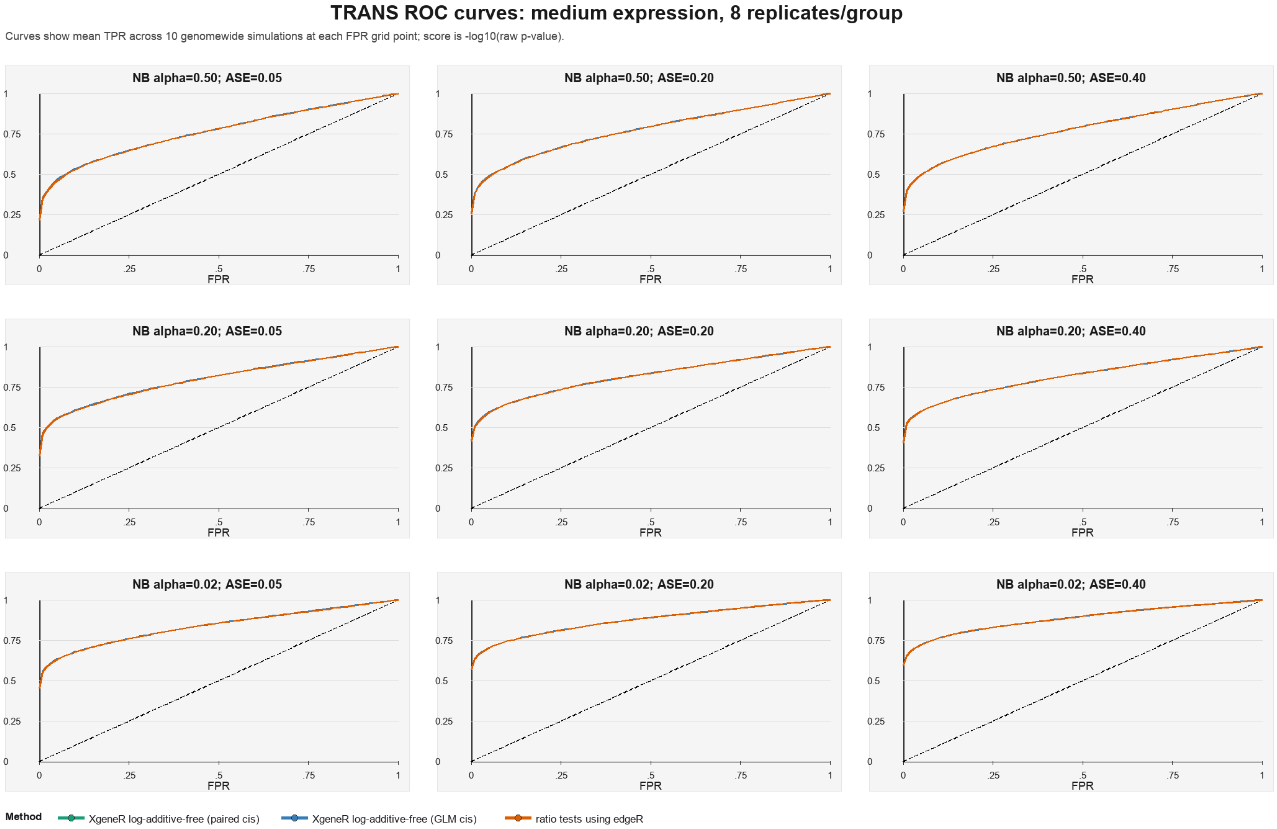


**Figure S18 ROC curves for trans detection in medium-expression genes with 8 replicates.** Curves compare XgeneR with ratio tests using edgeR across NB dispersion and ASE-informative fraction panels. NB alpha is the negative-binomial dispersion parameter used in count generation, with larger values indicating greater overdispersion; ASE is the simulated allele-specific-expression-informative fraction of hybrid reads. Each genomewide simulation contained 6000 genes assigned to low-, medium-, or high-expression categories; for plots stratified by expression, the reported measure was calculated separately within each expression category. For these summaries, the XgeneR log-additive model and the ratio tests are equivalent, so one curve is plotted directly underneath the other and is not visible.

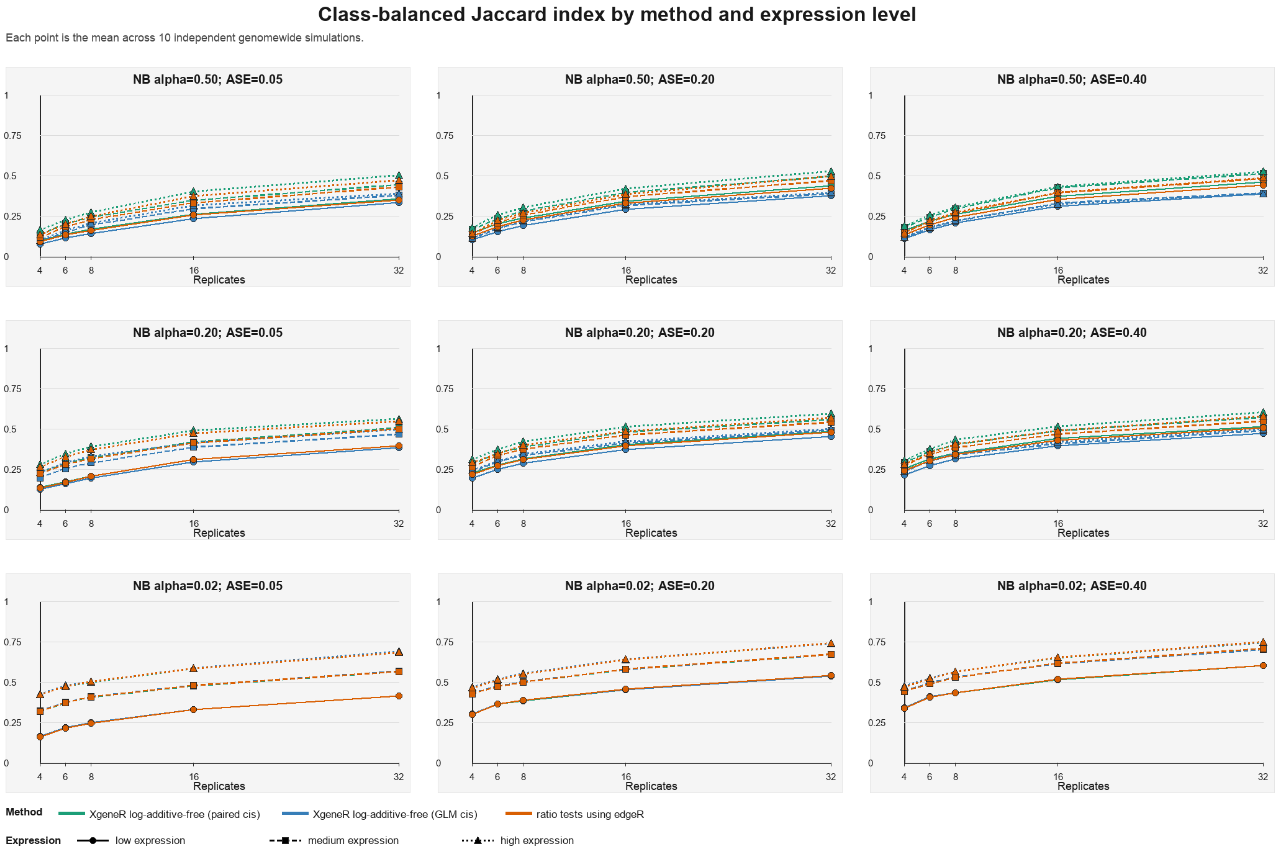


**Figure S19 Class-balanced Jaccard index across simulation parameters.** The class-balanced Jaccard index averages category-specific Jaccard values across the five true regulatory classes. NB alpha is the negative-binomial dispersion parameter used in count generation, with larger values indicating greater overdispersion; ASE is the simulated allele-specific-expression-informative fraction of hybrid reads. Each genomewide simulation contained 6000 genes assigned to low-, medium-, or high-expression categories; for plots stratified by expression, the reported measure was calculated separately within each expression category. For these summaries, the XgeneR log-additive model and the ratio tests are equivalent, so one curve is plotted directly underneath the other and is not visible.

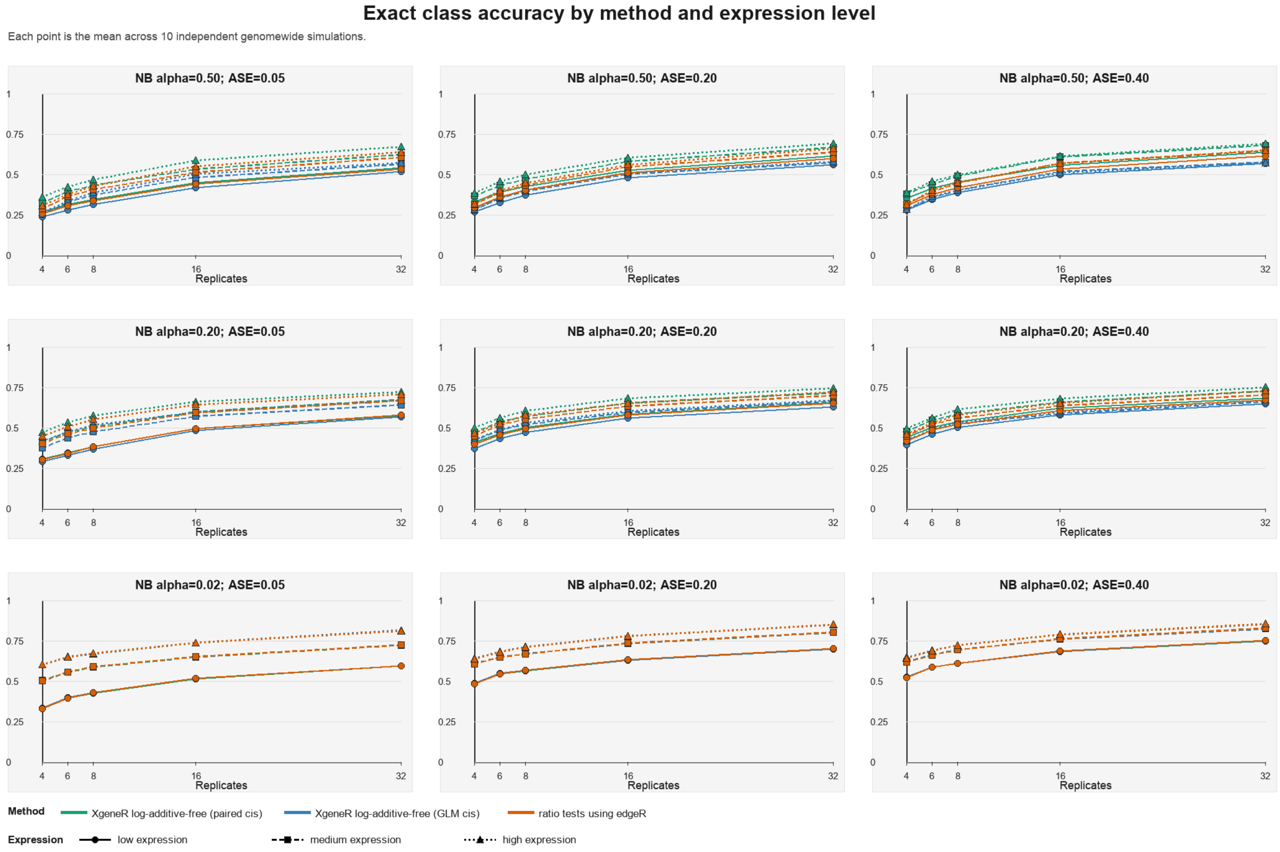


**Figure S20 Exact five-class accuracy across simulation parameters.** Exact accuracy is the fraction of genes whose inferred regulatory class exactly matched the simulated truth. NB alpha is the negative-binomial dispersion parameter used in count generation, with larger values indicating greater overdispersion; ASE is the simulated allele-specific-expression-informative fraction of hybrid reads. Each genomewide simulation contained 6000 genes assigned to low-, medium-, or high-expression categories; for plots stratified by expression, the reported measure was calculated separately within each expression category.

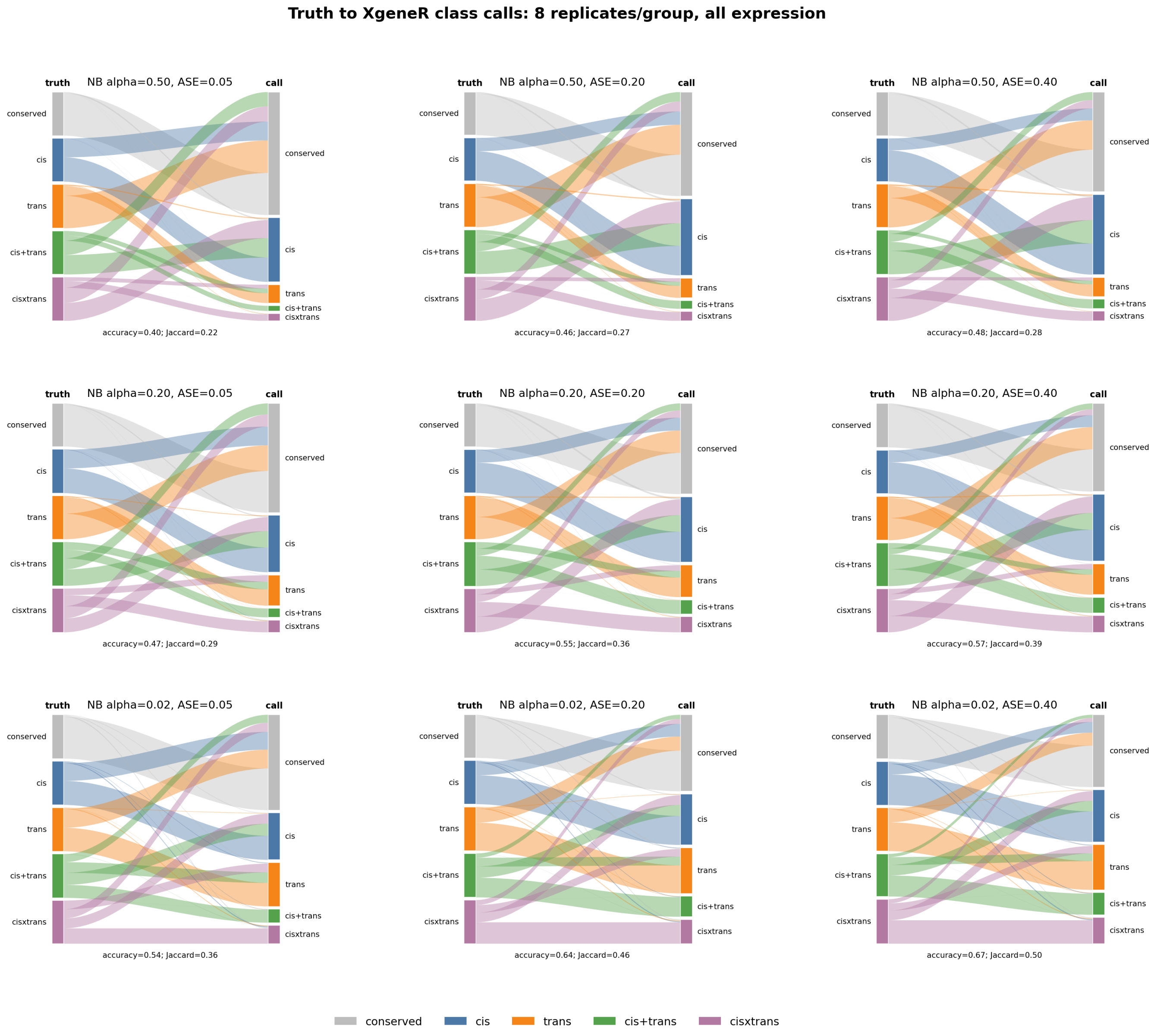


**Figure S21 Alluvial plot from true class to XgeneR class with 8 replicates.** Each panel represents a combination of NB dispersion and ASE-informative fraction; flows show how simulated truth maps to XgeneR calls. NB alpha is the negative-binomial dispersion parameter used in count generation, with larger values indicating greater overdispersion; ASE is the simulated allele-specific-expression-informative fraction of hybrid reads. Each genomewide simulation contained 6000 genes assigned to low-, medium-, or high-expression categories; for plots stratified by expression, the reported measure was calculated separately within each expression category.

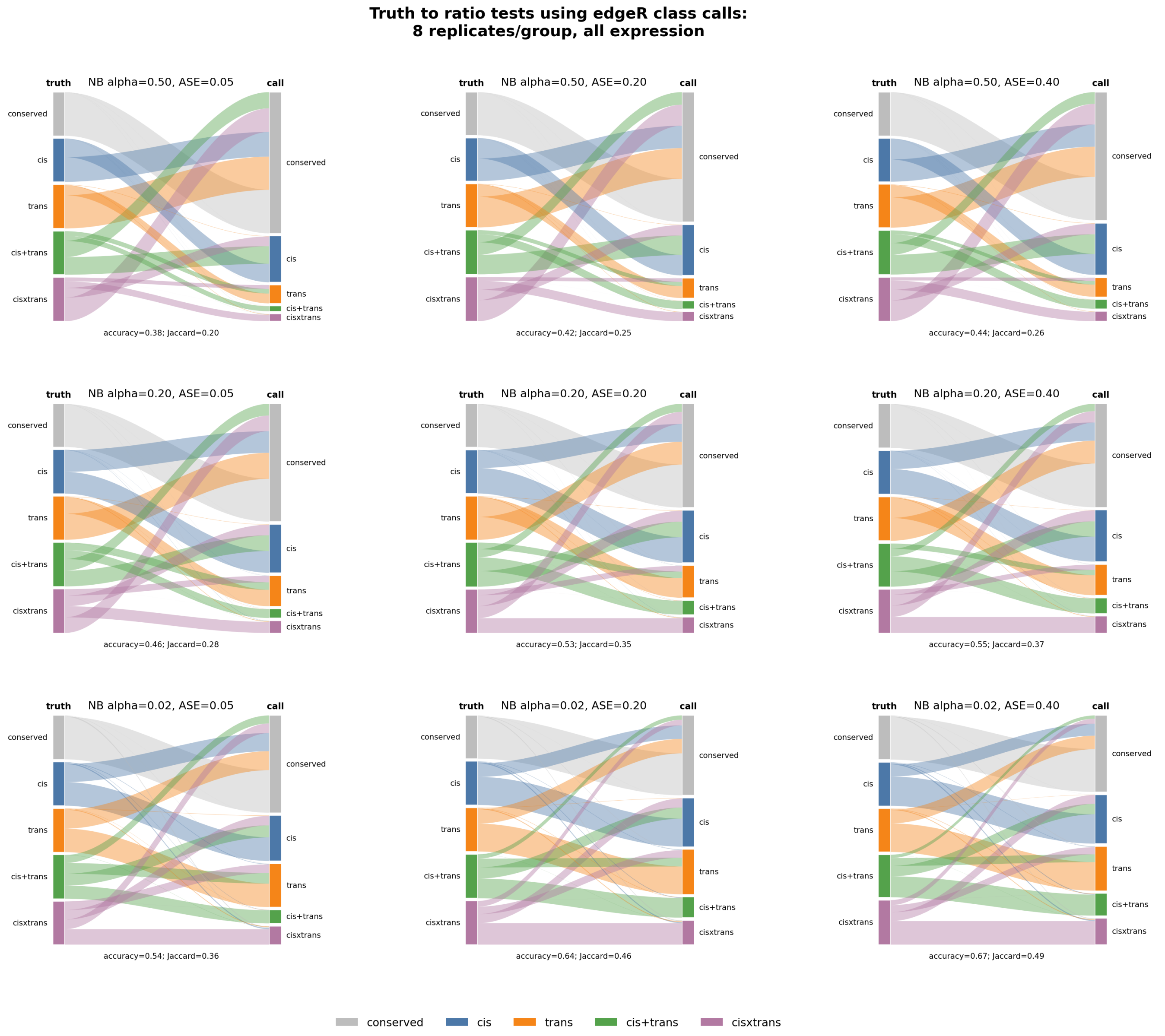


**Figure S22 Alluvial plot from true class to classes assigned by ratio tests using edgeR with 8 replicates.** Each panel represents a combination of NB dispersion and ASE-informative fraction; flows show how simulated truth maps to calls from ratio tests using edgeR. NB alpha is the negative-binomial dispersion parameter used in count generation, with larger values indicating greater overdispersion; ASE is the simulated allele-specific-expression-informative fraction of hybrid reads. Each genomewide simulation contained 6000 genes assigned to low-, medium-, or high-expression categories; for plots stratified by expression, the reported measure was calculated separately within each expression category.

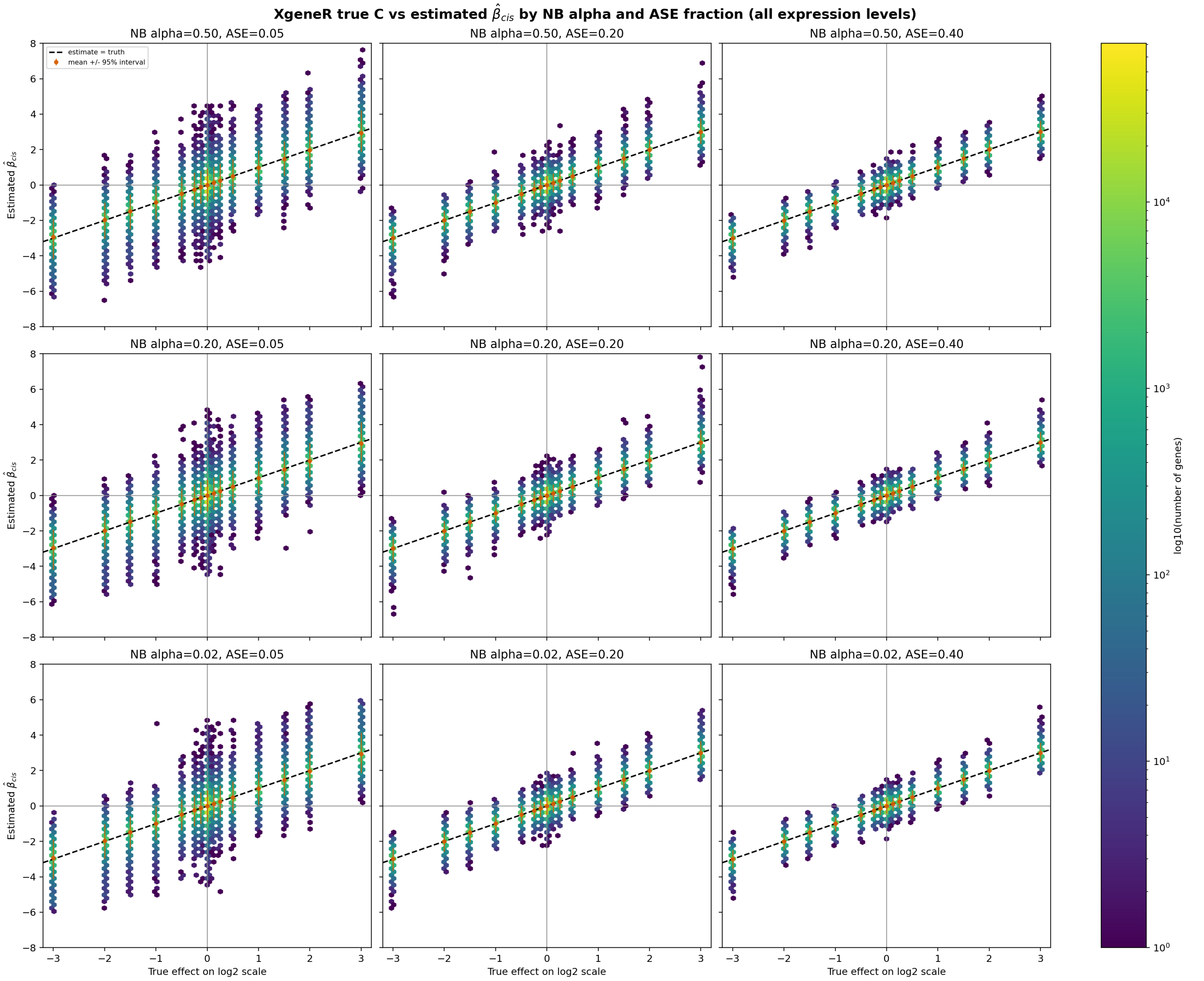


**Figure S23 XgeneR true-versus-estimated cis contrast.** Scatterplots compare true simulated $C$ values with XgeneR estimated cis coefficients, stratified by NB dispersion and ASE-informative fraction. NB alpha is the negative-binomial dispersion parameter used in count generation, with larger values indicating greater overdispersion; ASE is the simulated allele-specific-expression-informative fraction of hybrid reads. Each genomewide simulation contained 6000 genes assigned to low-, medium-, or high-expression categories; for plots stratified by expression, the reported measure was calculated separately within each expression category.

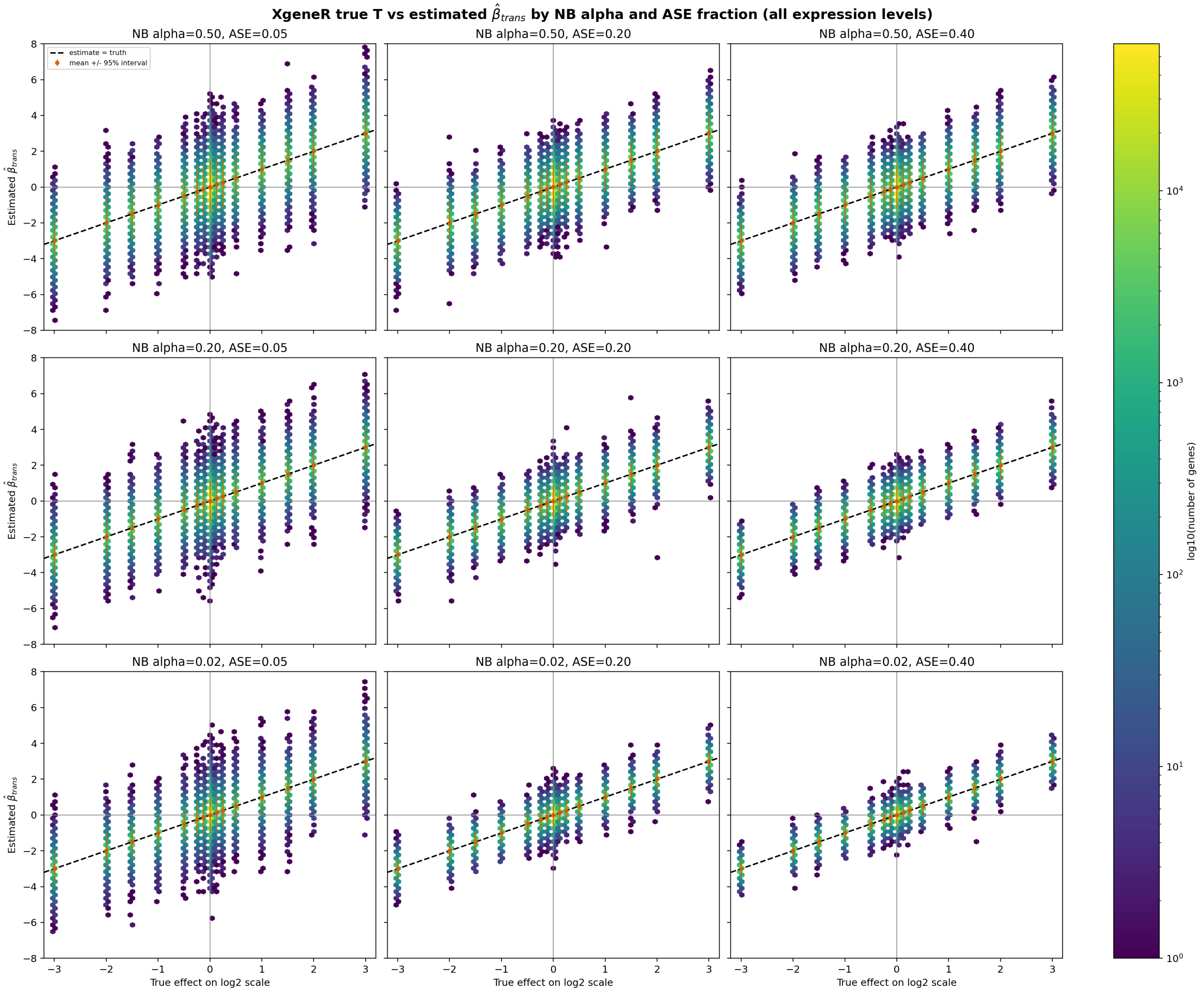


**Figure S24 XgeneR true-versus-estimated trans contrast.** Scatterplots compare true simulated $T$ values with XgeneR estimated trans coefficients, stratified by NB dispersion and ASE-informative fraction. NB alpha is the negative-binomial dispersion parameter used in count generation, with larger values indicating greater overdispersion; ASE is the simulated allele-specific-expression-informative fraction of hybrid reads. Each genomewide simulation contained 6000 genes assigned to low-, medium-, or high-expression categories; for plots stratified by expression, the reported measure was calculated separately within each expression category.

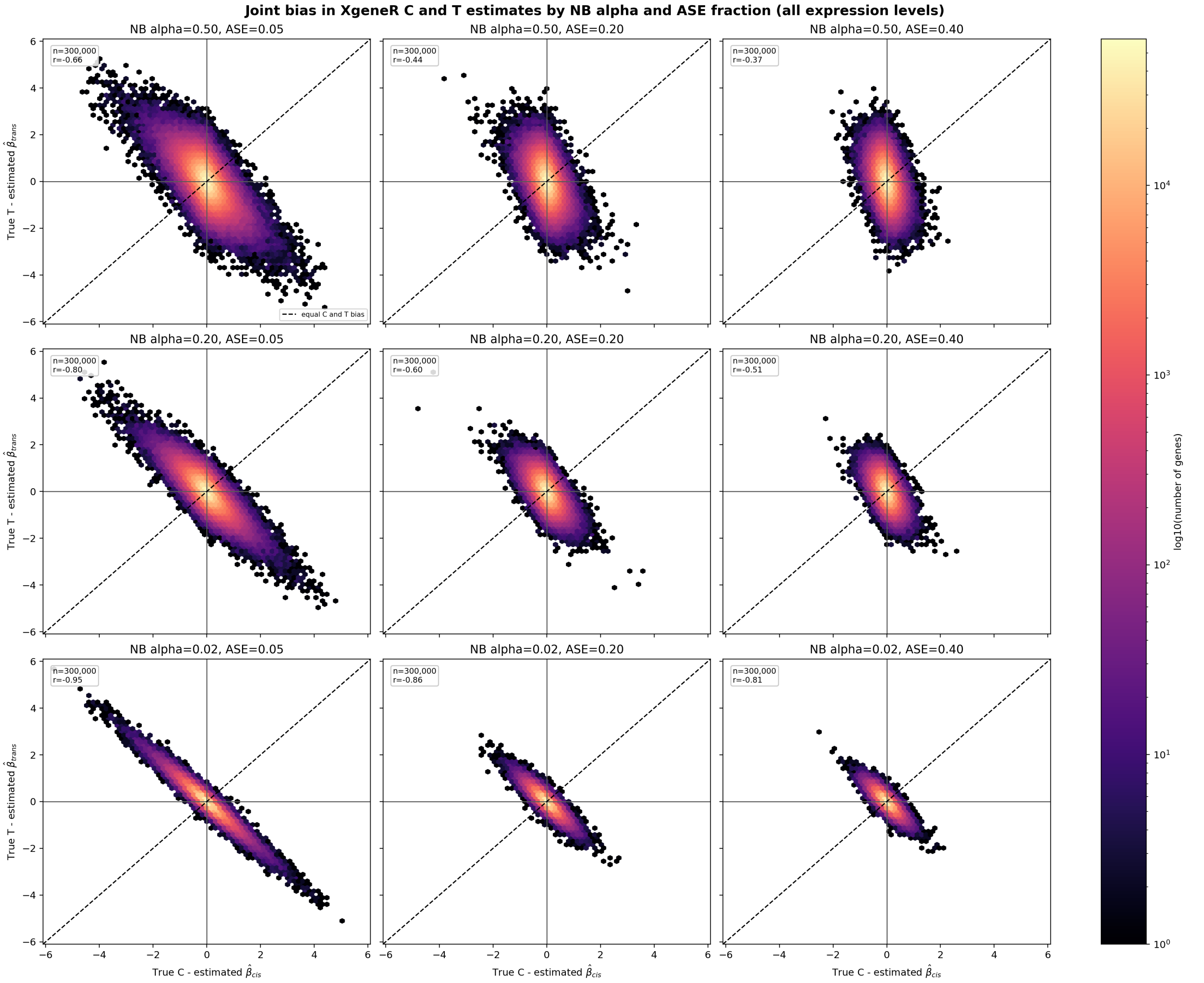


**Figure S25 Joint bias in XgeneR cis and trans estimates.** The x-axis is true $C$ minus estimated cis effect and the y-axis is true $T$ minus estimated trans effect, stratified by NB dispersion and ASE-informative fraction. NB alpha is the negative-binomial dispersion parameter used in count generation, with larger values indicating greater overdispersion; ASE is the simulated allele-specific-expression-informative fraction of hybrid reads. Each genomewide simulation contained 6000 genes assigned to low-, medium-, or high-expression categories; for plots stratified by expression, the reported measure was calculated separately within each expression category.

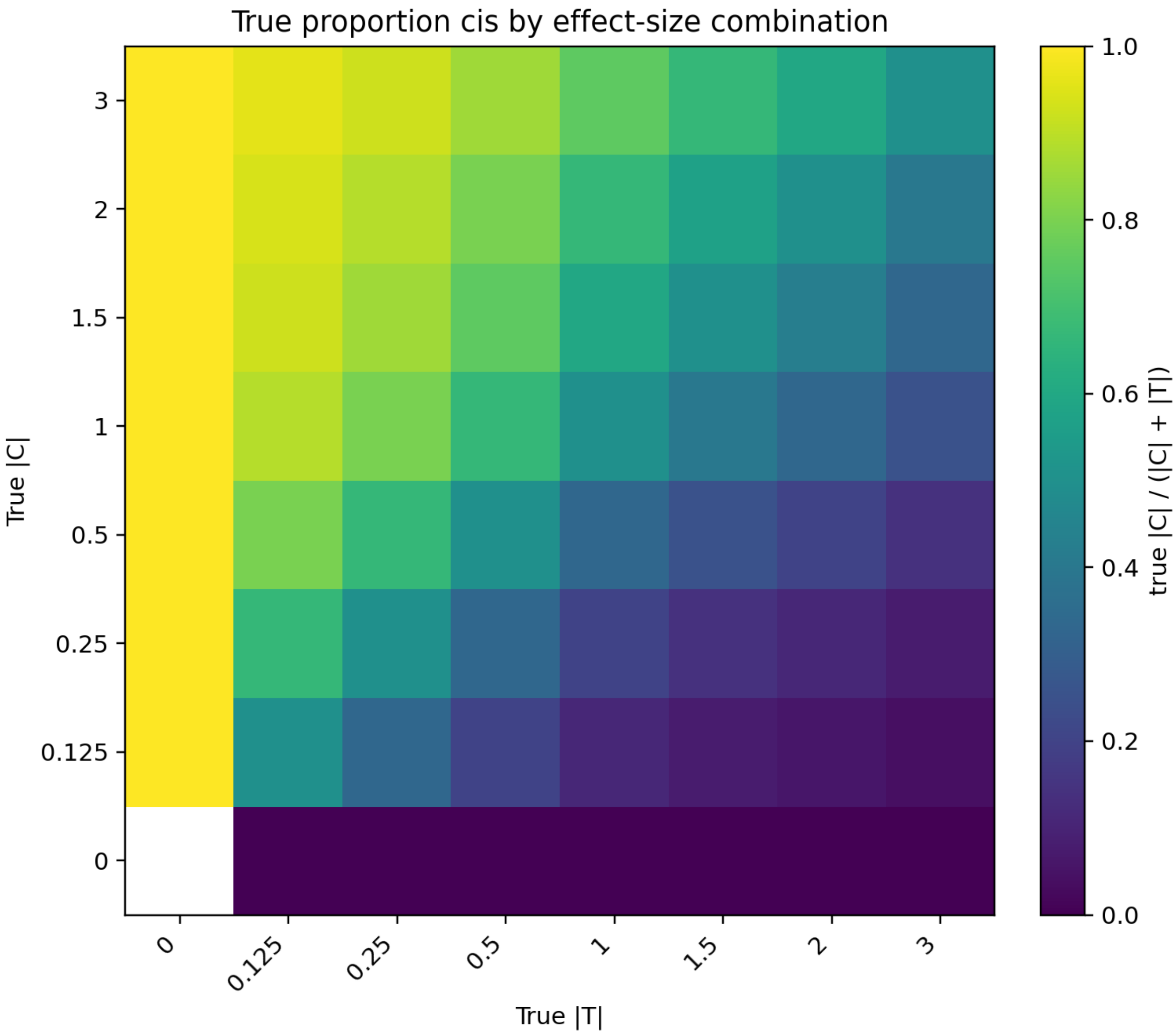


**Figure S26 True proportion-cis values implied by simulated effect combinations.** Proportion cis is defined as $|C|/(|C|+|T|)$ for genes with at least one nonzero regulatory effect.

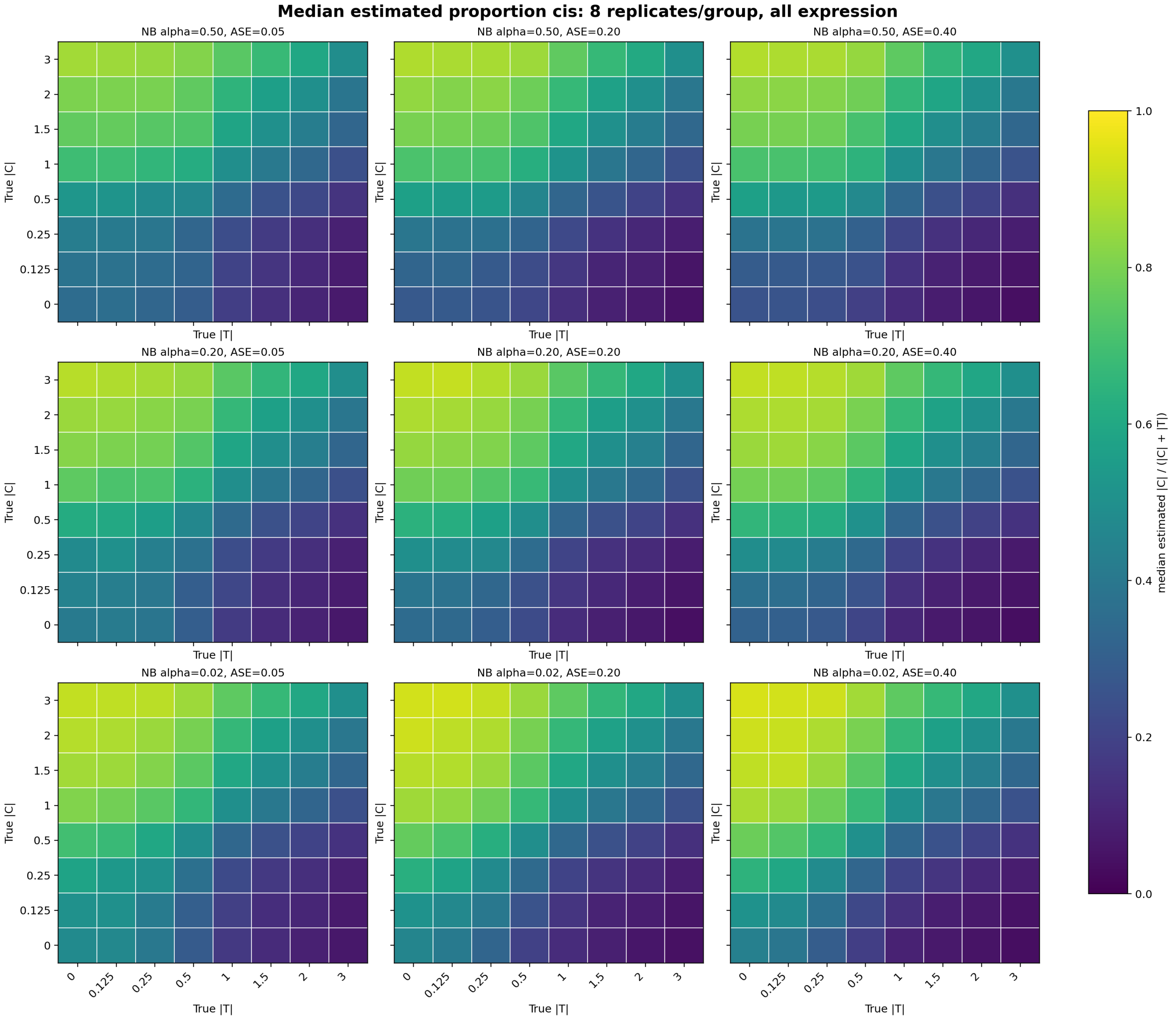


**Figure S27 Median estimated proportion cis with 8 replicates.** The plot summarizes the median XgeneR estimated proportion cis across true cis/trans effect-size combinations and simulation settings. NB alpha is the negative-binomial dispersion parameter used in count generation, with larger values indicating greater overdispersion; ASE is the simulated allele-specific-expression-informative fraction of hybrid reads. Each genomewide simulation contained 6000 genes assigned to low-, medium-, or high-expression categories; for plots stratified by expression, the reported measure was calculated separately within each expression category.

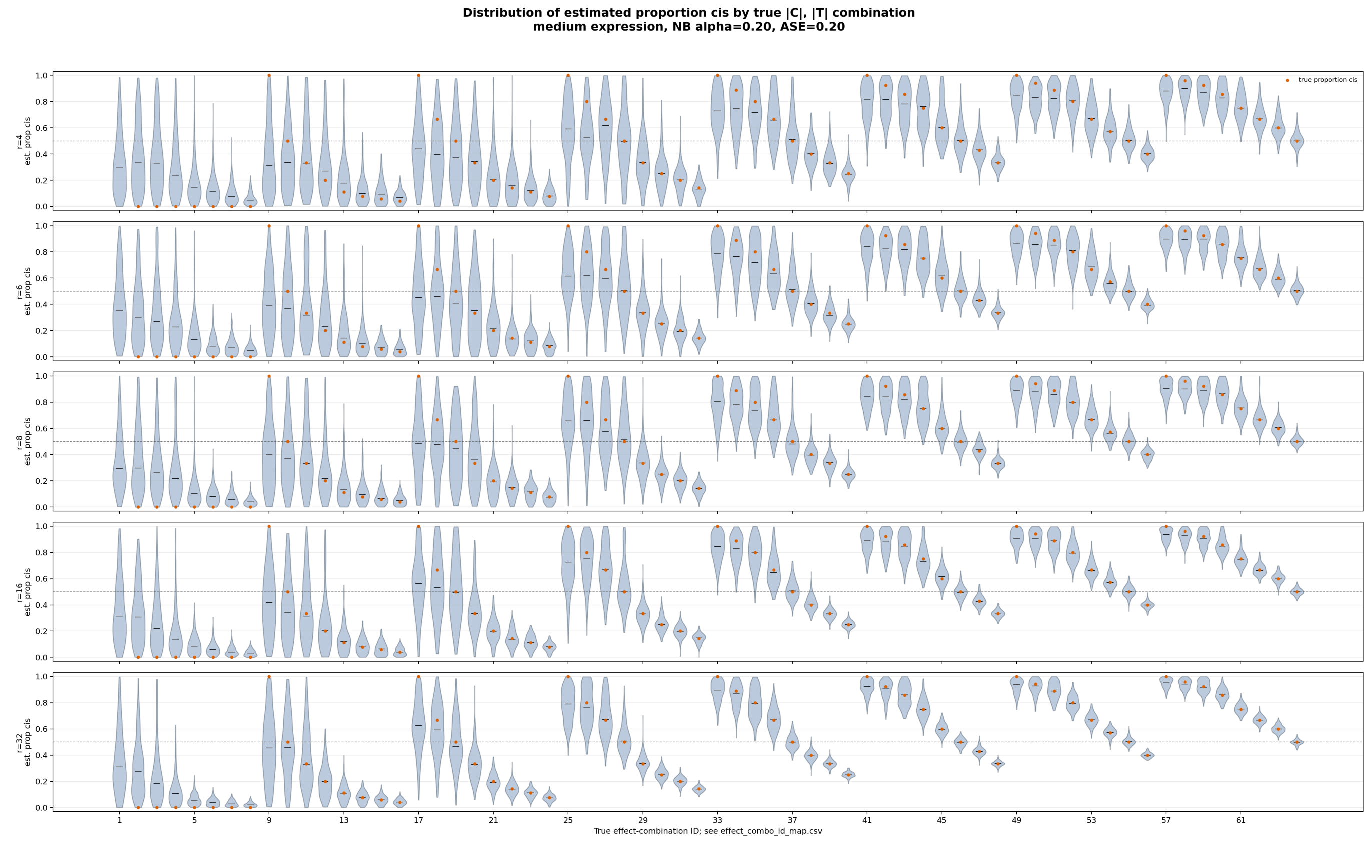


**Figure S28 Distribution of estimated proportion cis for medium-expression genes, NB alpha 0.20, and ASE fraction 0.20.** Violin plots show variation across genes and genomewide repeats for each true effect-size combination. NB alpha is the negative-binomial dispersion parameter used in count generation, with larger values indicating greater overdispersion; ASE is the simulated allele-specific-expression-informative fraction of hybrid reads. Each genomewide simulation contained 6000 genes assigned to low-, medium-, or high-expression categories; for plots stratified by expression, the reported measure was calculated separately within each expression category.
