# Supplementary material for: Estimating cis and trans contributions to differences in gene regulation
Source: Genetics. 2026 Sep 11;234(3):iyag228. doi: 10.1093/genetics/iyag228 (PMC13732733; doi:10.1093/genetics/iyag228)
Supplement: Supplementary file 2 [file iyag228_supplementary_data.zip › Supplemental_Material_Simulation_Methods_GENETICS-2026-309262.pdf]

Supplementary material, simulation methods, for: Estimating cis and trans contributions to differences in gene regulation

Ingileif B. Hallgrímsdóttir[1,*], Maria Carilli[1] and Lior Pachter[1,2]

[1]Division of Biology and Biological Engineering, California Institute of Technology, Pasadena, CA 91125, USA
[2]Department of Computing and Mathematical Sciences, California Institute of Technology, Pasadena, CA 91125, USA

*Corresponding author: Ingileif Hallgrimsdottir (ingileif@caltech.edu)

Abstract

This supplement summarizes a genomewide simulation study designed to evaluate cis/trans regulatory inference from parental and hybrid allele-specific count data. Each simulated genomewide data set contained 6000 genes assigned to low-, medium-, or high-expression categories, with simulations varying the number of biological replicates, negative-binomial dispersion, ASE-informative read fraction, and cis/trans effect sizes. We analyzed each simulated data set with XgeneR and with ratio tests based on R_P, R_H, and $R_P - R_H$ implemented using edgeR, then compared methods using power, type I error, empirical false discovery fraction, ROC/AUC, class agreement, alluvial summaries of regulatory class assignment, parameter recovery, and estimated proportion cis.

Keywords: gene regulation; simulation; RNA-seq

Methods

Simulation study of *cis*/*trans* regulatory classification

We performed a genome-scale simulation study to evaluate recovery of known *cis* and *trans* regulatory effects from replicated parental and F1 hybrid RNA-seq counts. The simulation was designed as a controlled diagnostic study and mimics a typical mouse dataset with two parental strains. It preserves several features that are important for parental/hybrid expression studies: overdispersed gene counts, heterogeneous expression levels, unequal sequencing depth, limited allele-specific-expression (ASE) informative reads in hybrids, and regulatory categories with known truth.

The simulation grid contains 45 experimental scenarios. Each scenario was simulated 10 independent times, and each genomewide replicate contained 6,000 genes. Thus, each method was evaluated on 450 genomewide datasets and 2,700,000 gene-level truth/call records. The complete grid varied biological replication, negative-binomial count dispersion, and the fraction of hybrid reads informative for ASE.

Regulatory classes and true effects. Each simulated gene was assigned to one of five true regulatory categories:

$$\text{conserved}, \quad \textit{cis}, \quad \textit{trans}, \quad \textit{cis} + \textit{trans}, \quad \textit{cis} \times \textit{trans}.$$

The class probabilities were equal, 0.20 for each category. Let C_g denote the identifiable *cis* contrast and T_g the identifiable *trans* contrast for gene g, both on the $\log_2$ expression scale. Conserved genes had $C_g = T_g = 0$. Genes in the *cis* class had $C_g \neq 0$ and $T_g = 0$; genes in the *trans* class had $C_g = 0$ and $T_g \neq 0$; genes in the *cis*+*trans* class had both effects nonzero and in the same direction; genes in the *cis*×*trans* class had both effects nonzero and in opposite directions.

For nonzero effects, absolute effect sizes were drawn uniformly from

$$\{0.125, 0.25, 0.5, 1.0, 1.5, 2.0, 3.0\}$$

on the $\log_2$ scale. These values correspond to fold changes ranging from approximately 1.09-fold to 8-fold. Effect signs were balanced within regulatory class and effect-size combination so that positive and negative effects were represented symmetrically. This avoids systematic library-size normalization artifacts that can occur if most simulated effects point in the same direction.

Full latent data-generating model. Counts were generated from the full latent model

$$\begin{aligned}
\eta_{P1,g} &= \mu_g + c_{1g} + t_{1g}, \\
\eta_{P2,g} &= \mu_g + c_{2g} + t_{2g}, \\
\eta_{H1,g} &= \mu_g + c_{1g} + \frac{1}{2} t_{1g} + \frac{1}{2} t_{2g}, \\
\eta_{H2,g} &= \mu_g + c_{2g} + \frac{1}{2} t_{1g} + \frac{1}{2} t_{2g},
\end{aligned}$$

where η is the $\log_2$ expected expression before library-size scaling. Because the absolute latent terms $c_{1g}, c_{2g}, t_{1g}, t_{2g}$ are not individually identifiable in a two-parent, one-hybrid design, the simulator used the centered convention

$$c_{1g} = -\frac{1}{2}C_g, \qquad c_{2g} = \frac{1}{2}C_g, \qquad t_{1g} = -\frac{1}{2}T_g, \qquad t_{2g} = \frac{1}{2}T_g.$$

Under this convention $C_g = c_{2g} - c_{1g}$ and $T_g = t_{2g} - t_{1g}$. The expected parent difference is $C_g + T_g$, and the expected hybrid allele difference is C_g.

Expression levels and library-size factors. Each gene was assigned a baseline mean count by sampling from

$$\{20, 100, 500\}$$

with probabilities 0.40, 0.40, and 0.20, respectively. These levels represent low, medium, and high expression after filtering. For every biological replicate, a library-size multiplier was sampled from

$$\{1.0, 1.25, 1.5, 2.0, 2.5\}.$$

The same set of possible library-size factors was used for parental and hybrid samples. Hybrid allele-specific counts were made smaller not by assigning a smaller hybrid library-size distribution, but by explicitly modeling the limited fraction of hybrid reads that are informative for ASE.

Negative-binomial count generation. For each parental sample, expected counts were obtained by exponentiating the corresponding latent linear predictor and multiplying by the sampled library-size factor L:

$$E(Y_{P1,g}) = 2^{\eta_{P1,g}}L, \qquad E(Y_{P2,g}) = 2^{\eta_{P2,g}}L.$$

Counts were then sampled from a negative-binomial distribution parameterized as

$$\mathrm{Var}(Y_g) = \mu_g + \alpha\mu_g^2.$$

The simulation varied

$$\alpha \in \{0.02, 0.20, 0.50\}.$$

These values represent low, moderate, and high count overdispersion. The high-dispersion setting was retained because preliminary runs showed that dispersion was one of the main limits on power, especially for trans-effect detection.

Hybrid count generation and ASE thinning. Hybrid counts were generated in a way that preserves the dependence between the two allele-specific counts from the same hybrid sample. For each hybrid replicate, the simulator first computed the full expected H1 and H2 counts from the latent model:

$$E(Y_{H1,g}^{\mathrm{full}}) = 2^{\eta_{H1,g}}L, \qquad E(Y_{H2,g}^{\mathrm{full}}) = 2^{\eta_{H2,g}}L.$$

The expected full hybrid total was the sum of these two expectations, and one full hybrid total count was drawn from the negative-binomial model:

$$Y_{H,g}^{\mathrm{full}} \sim \mathrm{NB}\left(E(Y_{H1,g}^{\mathrm{full}}) + E(Y_{H2,g}^{\mathrm{full}}), \alpha\right).$$

Conditional on this full hybrid total, the H1 count was generated from a beta-binomial distribution,

$$Y_{H1,g}^{\mathrm{full}} \mid Y_{H,g}^{\mathrm{full}} \sim \mathrm{BetaBinomial}\left(Y_{H,g}^{\mathrm{full}}, p_g, \kappa\right),$$

where

$$p_g = \frac{E(Y_{H1,g}^{\mathrm{full}})}{E(Y_{H1,g}^{\mathrm{full}}) + E(Y_{H2,g}^{\mathrm{full}})} + b.$$

The H2 count was set to $Y_{H,g}^{\mathrm{full}} - Y_{H1,g}^{\mathrm{full}}$. The reference-bias parameter was fixed at $b = 0$, and the beta-binomial concentration parameter was fixed at $\kappa = 80$.

Finally, the observed ASE-informative counts were generated by binomial thinning of the full allele-specific hybrid counts. We varied the ASE-informative fraction over

$$f_{\mathrm{ASE}} \in \{0.05, 0.20, 0.40\}.$$

Thus, the observed H1 and H2 counts are complementary allele-specific counts from the same full hybrid sample, but only a fraction of each allele's reads are retained as ASE-informative. This order of generation matches the biological intuition that one hybrid RNA-seq library has a total depth, the reads are divided between the two parental haplotypes, and only some reads are informative for allele-specific assignment.

Replication grid. We varied the number of biological replicates per parental strain and per hybrid cross over

$$n \in \{4, 6, 8, 16, 32\}.$$

The 4, 6, 8, 16, and 32 replicate settings provide a broad design grid. Each point in the grid had 10 independent genomewide repeats.

XgeneR analysis. Each simulated genomewide dataset was written as a gene-by-sample count matrix with one tissue (BAT), one environment (Control), two parental count groups (P1 and P2), and two hybrid allele-specific count groups (H1 and H2). The primary XgeneR analysis used the local XgeneR implementation with the `log_additive_free` trans model, `hybrid_total_tmm_half` normalization, and an FDR threshold of $\alpha = 0.10$. The `hybrid_total_tmm_half` normalization uses the simulated full hybrid total counts to estimate one hybrid library-size factor and then assigns half of that factor to each hybrid allele column, rather than normalizing H1 and H2 independently.

For the primary XgeneR classification, the cis p-value was obtained from a paired hybrid-allele test comparing H1 and H2 within hybrid replicate. This paired cis test is closely aligned with the ASE test in the analysis based on three ratio tests. We also ran a diagnostic XgeneR analysis using the GLM cis coefficient directly; those outputs were retained separately for method comparison but are not the primary classification used in the main simulation summaries.

Comparison to analysis based on ratio tests. For every simulated count matrix, we also ran classification analysis based on the three ratio tests. The tests were implemented with edgeR-style negative-binomial GLM tests. The three tests are: a parent test comparing P1 and P2 ($R_P = 0$), an ASE test comparing H1 and H2 in hybrids ($R_H = 0$), and a ratio/trans test comparing the parent difference with the hybrid allele difference ($R_P - R_H = 0$). The purpose of this comparison was not to exactly reproduce any one published implementation, but to compare XgeneR with the same three-test regulatory logic while using a count-modeling framework comparable to XgeneR.

Under the log-additive model, the three contrasts $R_P, R_H, R_P - R_H$ correspond to

$$\eta_{P2,g} - \eta_{P1,g} = C_g + T_g,$$

$$\eta_{H2,g} - \eta_{H1,g} = C_g,$$

$$(\eta_{P2,g} - \eta_{P1,g}) - (\eta_{H2,g} - \eta_{H1,g}) = T_g.$$

Thus, the ASE test targets the cis contrast, the ratio/trans test targets the trans contrast, and the parent test asks whether the total parental divergence is nonzero.

Performance summaries. We assessed each method using the known simulated truth. The main summaries included exact five-class accuracy, class-balanced F-score, class-balanced Jaccard index, cis-component power, trans-component power, cis and trans false positive rates among true-null genes, empirical false discovery fractions among calls, ROC curves, and AUC. Cis-component power was defined as the fraction of genes with a true cis component that were called as any cis-containing class. Trans-component power was defined analogously. We also stratified power and AUC by expression level, NB dispersion, ASE-informative fraction, replicate count, and true absolute effect size.

For category-specific classification agreement, the Jaccard index for class k was

$$J_k = \frac{\mathrm{TP}_k}{\mathrm{TP}_k + \mathrm{FP}_k + \mathrm{FN}_k}.$$

The class-balanced Jaccard index is the unweighted mean across the five regulatory classes, giving conserved, *cis*, *trans*, *cis+trans*, and *cis*×*trans* equal importance. We also generated alluvial plots showing the flow from true simulated category to inferred category for XgeneR and for the ratio test analysis.

Parameter grid. The simulation grid was the Cartesian product

$$n \in \{4, 6, 8, 16, 32\}, \qquad \alpha \in \{0.02, 0.20, 0.50\}, \qquad f_{\mathrm{ASE}} \in \{0.05, 0.20, 0.40\}.$$

The number of genomewide repeats per scenario was 10, reference bias was fixed at 0, beta-binomial concentration was fixed at 80, and each simulated genomewide dataset contained 6,000 genes.
